# Cooperative C–H activation of pyridine by PBP complexes of Rh and Ir can lead to bridging 2-pyridyls with different connectivity to the B–M unit†

**DOI:** 10.1039/d1sc01850g

**Published:** 2021-10-05

**Authors:** Yihan Cao, Wei-Chun Shih, Nattamai Bhuvanesh, Jia Zhou, Oleg V. Ozerov

**Affiliations:** Department of Chemistry, Texas A&M University 3255 TAMU College Station Texas 77842 USA ozerov@chem.tamu.edu; State Key Laboratory of Urban Water Resource and Environment, Harbin Institute of Technology Harbin 150090 China jiazhou@hit.edu.cn

## Abstract

Pyridine and quinoline undergo selective C–H activation in the 2-position with Rh and Ir complexes of a boryl/bis(phosphine) PBP pincer ligand, resulting in a 2-pyridyl bridging the transition metal and the boron center. Examination of this reactivity with Rh and Ir complexes carrying different non-pincer ligands on the transition metal led to the realization of the possible isomerism derived from the 2-pyridyl fragment connecting either *via* B–N/C–M bonds or *via* B–C/N–M bonds. This M–C/M–N isomerism was systematically examined for four structural types. Each of these types has a defined set of ligands on Rh/Ir besides 2-pyridyl and PBP. A pair of M–C/M–N isomers for each type was computationally examined for Rh and for Ir, totaling 16 compounds. Several of these compounds were isolated or observed in solution by experimental methods, in addition to a few 2-quinolyl variants. The DFT predictions concerning the thermodynamic preference within each M–C/M–N isomeric match the experimental findings very well. In two cases where DFT predicts <2 kcal mol^−1^ difference in free energy, both isomers were experimentally observed in solution. Analysis of the structural data, of the relevant Wiberg bond indices, and of the ETS-NOCV partitioning of the interaction of the 2-pyridyl fragment with the rest of the molecule points to the strength of the M–C(pyridyl) bond as the dominant parameter determining the relative M–C/M–N isomer favorability. This M–C bond is always stronger for the analogous Ir *vs.* Rh compounds, but the nature of the ligand *trans* to it has a significant influence, as well. DFT calculations were used to evaluate the mechanism of isomerization for one of the molecule types.

## Abstract 2

The thermodynamic preference between two isomeric products of C–H activation of pyridine, with 2-pyridyl bridging boron and iridium or rhodium, primarily depends on the M–C bond strength.

## Graphical abstract

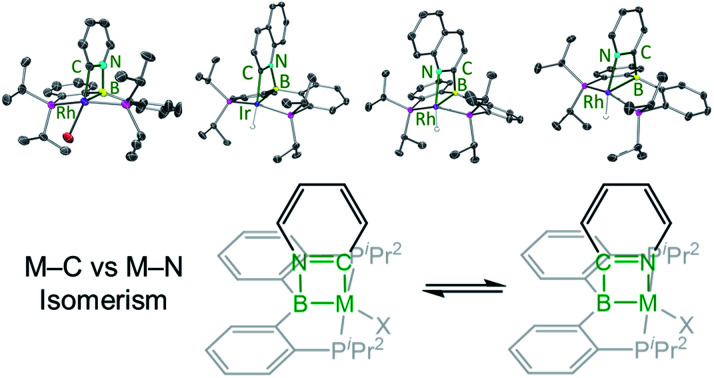

## Introduction

Selective C–H activation and functionalization of pyridines and other azines presents special challenges, in part because these heterocycles can function as good ligands towards many transition metals.^1,2^ Selectivity for the 3- (or *meta*-) position is more common with transition metals,^3–5^ but studies of selective 2-position functionalization are also known.^4,6–12^ In many specific cases, the scope may be limited, and a particular substitution pattern on the azine is often required for selectivity.

**Fig. 1:**
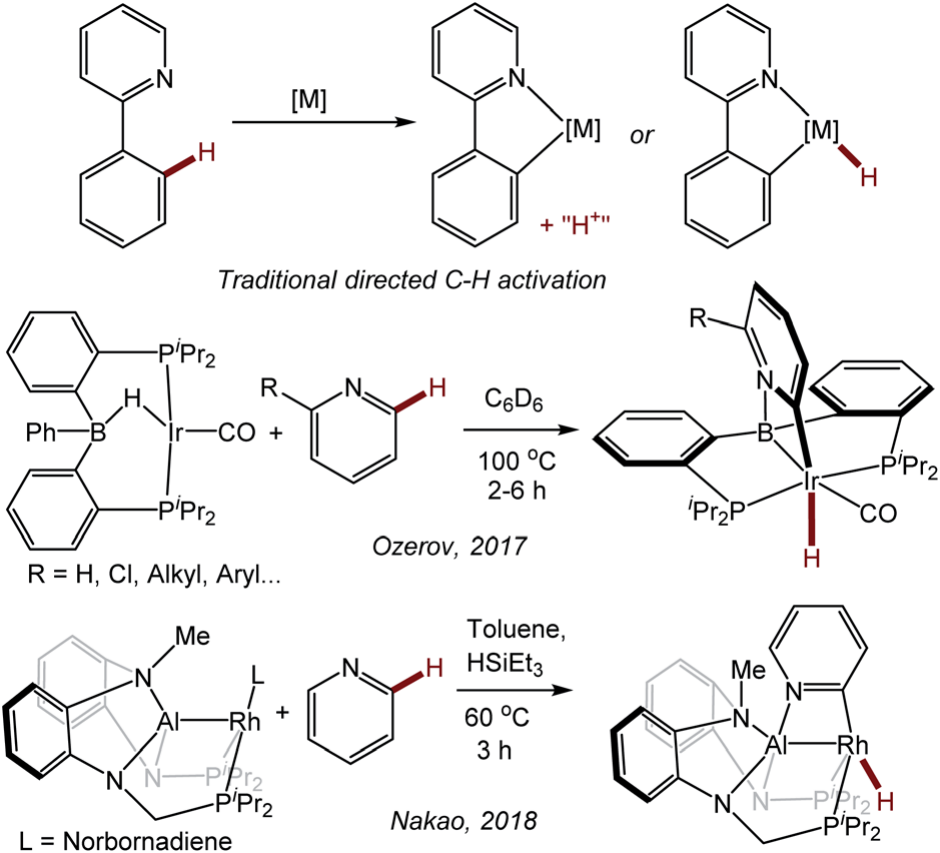
Traditional directed C–H activation of 2-phenylpyridine (top) and boryl- or aluminyl-directed C–H activation of the 2-position of a pyridine ring.

In 2017, we reported a new approach to the directed activation of C–H bonds in pyridine derivatives using an Ir system supported by a boryl/bis(phosphine) PBP^13^ pincer^14,15^ ligand.^16^ The binding of the pyridine (or quinoline) nitrogen to the Lewis acidic boryl site directs Ir to the 2-position in the heterocycle. This approach is distinct from the more classical directed C–H activation, where the directing group donor binds to the same atom (transition metal) which effects C–H cleavage (Fig. 1).^17–21^ Pyridine derivatives have played a prominent role in the development of classical directed C–H activation,^19,20^ but they typically direct the metal not to the C–H bonds of the pyridine ring itself, but to the more remote C–H bonds in a substituent, such as in the 2-phenyl group. We reasoned that the (PBP)Ir system preferred the C–H activation of the pyridine ring because of the favorability of the Ir/C/N/B trapezoidal four-membered ring formation.^16^ Some of the aspects of the mechanism of pyridine activation in our PBP system were recently studied computationally by Ke and coworkers.^22^ A similar selectivity was observed by Nakao *et al.* in the C–H activation of pyridines with a Rh complex^23^ supported by a closely related aluminyl/bis(phosphine) PAlP pincer (Fig. 1).^24,25^

Given Nakao's precedent with Rh, we wished to explore the reactivity with pyridine using the (PBP)Rh system,^26,27^ as well as the variations of the Rh and Ir systems with and without the carbonyl ligand. While exploring the analogous reactivity with (PBP)Rh, we came across an unexpected finding. As with Ir, C–H activation of pyridine resulted in the formation of a 2-pyridyl that is bridging the B–Rh bond. However, the connectivity was reversed, with C of the pyridyl attached to B and the N atom of the pyridyl attached to Rh. This prompted us to explore this M–C/M–N isomerism in more systematic detail, as it does not appear to have been considered in the literature. This report describes our analysis of the isomeric preference of the 2-pyridyl (or 2-quinolyl) fragment bridging the B–Ir or B–Rh bond in a series of compounds supported by the PBP pincer.

## Results and discussion

### Compounds under consideration and nomenclature

**Fig. 2:**
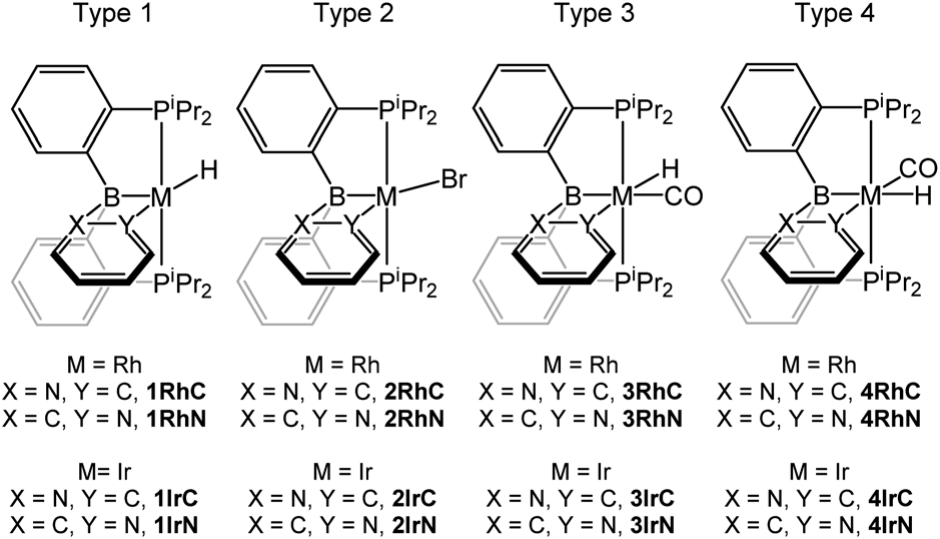
The four structural types under study in this work.

**Scheme 1:**
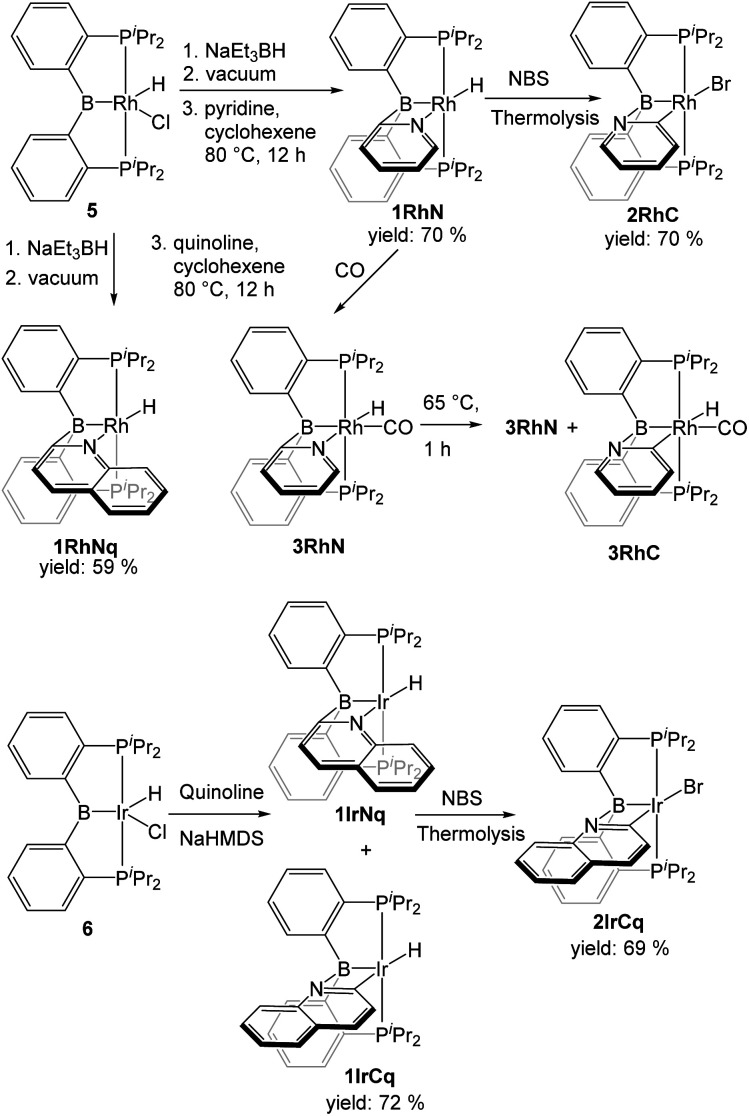
Synthesis of (PBP)Rh/Ir pyridyl complexes.

We selected four structural types for analysis (Fig. 2). For each type, we considered M–C/M–N isomerism for the Rh and for the Ir version, resulting in sixteen 2-pyridyl compounds whose structures were optimized computationally. The compound labels (Fig. 2) are derived from the general type (numeral) and the bond present between the metal (Rh or Ir) and C or N. Some of these compounds were isolated or observed experimentally in this (Scheme 1) or the previous report.^16^ In addition, we synthesized a few 2-quinolyl analogs of the 2-pyridyl compounds (Scheme 1); they are denoted by adding a “**q**” to the compound label. The type 3 and type 4 compounds are isomeric. We did not attempt the syntheses of the type 4 compounds because DFT calculations indicated that they are considerably higher in energy than the corresponding type 3 isomers (*vide infra*).

### Synthesis of Rh and Ir complexes

In order to access a Rh species capable of C–H activation, the previously reported 5 was treated with NaBEt_3_H followed by the removal of volatiles. Although the stoichiometry suggests the formation of “(PBP)RhH_2_”, we have not established the nature of the resultant species; from the *in situ* NMR observations, it appears that a mixture of a few complexes forms (Fig. S1†). Nonetheless, thermolysis of this mixture in the presence of cyclohexene and either pyridine or quinoline led to the formation of complexes 1RhN and 1RhNq, with an isolated yield of 70% and 59% respectively. The corresponding type 1 Ir compound 1IrCq was prepared by the treatment of (PBP)IrHCl with NaN(SiMe_3_)_2_ in the presence of quinoline. Compound 1IrCq exists in equilibrium with the minor isomer 1IrNq (1.00 : 0.055 ratio at 25 °C and 1.00 : 0.095 at 65 °C). Attempts to prepare 1IrC in a pure form were not successful. Unlike 1RhN, 1IrC appeared to bind an extra equivalent of pyridine, which resulted in a mixture of products when one equiv. of pyridine was used. Utilization of 3 equiv. of pyridine permitted observation of the pyridine adduct of 1IrC as the dominant product by NMR spectroscopy, but we did not pursue its isolation in a pure solid form (compound 7, Fig. S4†).

The conversion of the hydride complexes 1RhN and 1IrCq to the bromide derivatives 2RhC and 2IrCq was effected by thermolysis with NBS. Good isolated yields (70% and 69% respectively) were obtained after workup. No evidence of the presence of 2RhN or 2IrNq was noted.

The carbonyl adduct 3RhN was prepared by exposure of 1RhN to carbon monoxide and characterized *in situ* in solution after 10 min. After removing the excess carbon monoxide under vacuum, thermolysis of the solution of 3RhN in C_6_D_6_ for 1 h at 65 °C resulted in the formation of a mixture of 3RhN and 3RhC in a 1.0 : 0.08 ratio. Extended thermolysis for 24 h at 65 °C led to the formation of multiple complexes along with 3RhN and 3RhC, but in that mixture 3RhN was still present in a much higher concentration than 3RhC. The synthesis of the analogous Ir complex 3IrC was previously reported. The synthesis involved extended thermolysis at 100 °C and no evidence of the presence of 3IrN was noted.

### Spectroscopic characterization

The compounds explored in this study are rich in NMR active nuclei (^1^H, ^13^C, ^31^P, ^11^B, and ^103^Rh) (Table 1). All of the compounds possess *C*_s_-symmetry on the NMR time scale. The M–C/M–N isomers can be distinguished based on the relative ^1^H NMR chemical shift of the Rh/Ir–H signal. Since N of pyridyl is less *trans*-influencing than C of 2-pyridyl, a hydride *trans* to N appears at a more upfield frequency *vs.* a hydride *trans* to C. For the Rh compounds 1RhN, 1RhNq, and 3RhN with a hydride *trans* to N, its ^1^H NMR chemical shift falls into a narrow range of −15.7 to −17.3 ppm, but for 3RhC, the hydride resonates considerably upfield at *δ* −11.04 ppm. The contrast is even greater for the Ir pair 1IrCq (*δ* −0.20 ppm) and 1IrNq (*δ* −17.10 ppm).

The shape of the ^13^C{^1^H} NMR resonance corresponding to the boron- or metal-bound carbon of the 2-pyridyl or 2-quinolyl unit is also telling. In compounds 1RhN, 1RhNq, and 3RhN, this carbon is bound to boron and the corresponding ^13^C NMR resonances in these compounds possess some broadness. In compounds RhBr-C and IrH-Cq, this carbon is bound do the metal and displays coupling to the two equivalent ^31^P nuclei, as well as to ^103^Rh in RhBr-C.

**Table 1:** Selected NMR chemical shift data (in ppm, C_6_D_6_, solvent) for the experimentally observed complexes of types 1–3

Complexes	Rh/Ir–H^a^	^11^B{^1^H}	Ir/Rh–C^b^	B–C^c^
1RhN	−17.25	3.5	—	188.2
1RhNq	−16.81	4.5	—	189.5
1IrCq	−0.20	−8.5	201.3	—
1IrNq	−17.10	—d	—	—d
2RhC	—	1.7	178.0	—
2IrCq^e^	—	−6.8	176.9	—
3RhN	−15.69	2	—	193.5
3RhC	−11.04	—d	—d	—
3IrC	−14.15		164.9	

a ^1^H NMR chemical shift of the metal-bound hydride.

b ^13^C NMR chemical shift of the metal-bound carbon.

c ^13^C NMR chemical shift of the boron-bound carbon in the bridging pyridyl or quinolyl.

d Resonance was not observed due to low concentration.

e Spectra of 2IrCq were recorded in CDCl_3_.

### XRD structural characterization

**Fig. 3:**
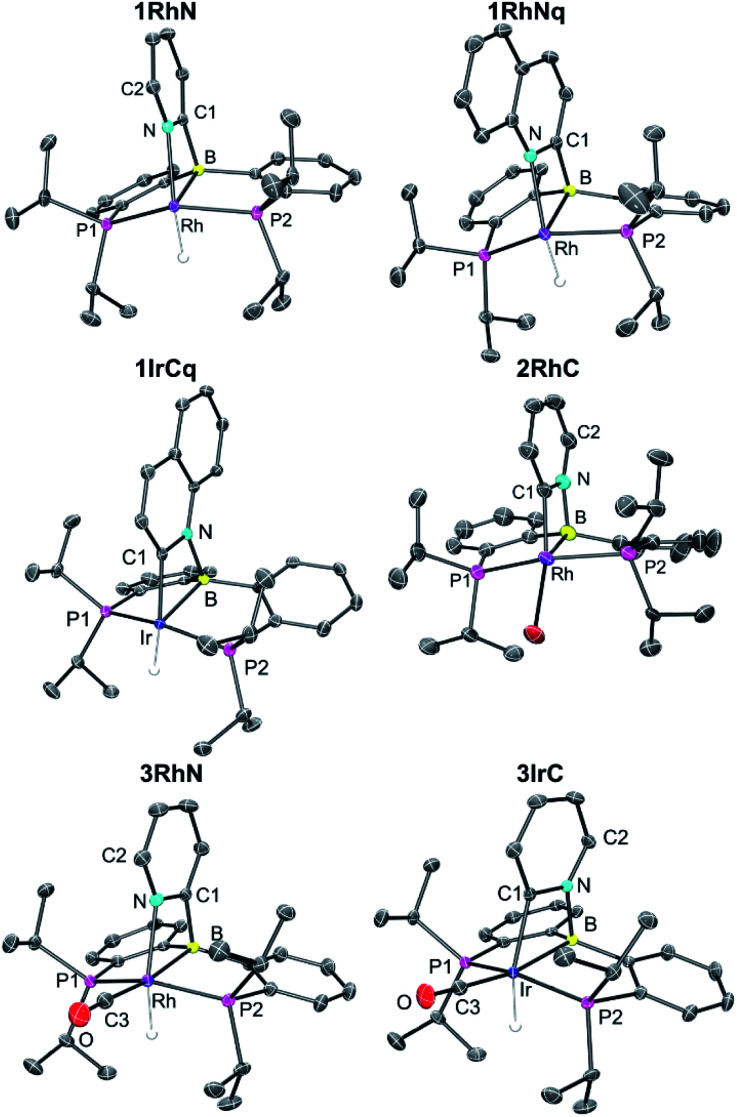
POV-Ray rendition of the ORTEP drawing (50% thermal ellipsoids) of 1RhN, 1RhNq, 1IrCq, 2RhC, and 3RhN showing selected atom labelling. Hydrogen atoms, disorders of ^i^Pr groups in 1IrCq and 2RhC crystals, and solvent molecules (toluene) in 1RhNq and 1IrCq crystals are omitted for clarity. Only one of the 1IrCq in the asymmetric unit is shown in the ORTEP drawing above.

Single crystal X-ray diffractometry permitted the determination of the solid-state structures of 1RhN, 1RhNq, 1IrCq, 2RhC, and 3RhN. The solid-state structure of 3IrC was reported in 2017 (Fig. 3).

The Ir–B (2.209(2) and 2.195(2) Å) and the Ir–C distances (2.034(3) and 2.029(3) Å) in the two crystallographically independent molecules of 1IrCq are slightly shorter than the Ir–B distance of 2.285(2) Å and the Ir–C distance of 2.079(2) Å in the previously reported 3IrC. The B–N and N–C distances in these molecules are very similar. Comparing the Rh–B distances in 1RhN (2.229(2) Å) and 3RhN (2.319(1) Å) also shows that the presence of CO is correlated with the elongation of the M–B bond (*trans* to CO) by almost 0.1 Å. However, the Rh–N distances (2.163(1) Å in 1RhN and 2.158(1) Å in 3RhN) seem to be unaffected by the presence of the CO ligand.

The values for the sum of angles that exclude the pyridyl/quinolyl nitrogen about the boron atom in 1RhN, 1RhNq and 3RhN are in the *ca.* 339.4°–343.8° range. The range of the corresponding values (excluding the pyridyl/quinolyl carbon) in 1IrCq, 2RhC and 3IrC is *ca.* 335.6°–341.9°. Likewise, the P–M–P angles in the six structures in Fig. 3 all fall within the *ca.* 151–161° range. Thus. while there are significant differences in the metrics of the M–C/N–B cycle among the six structures, the conformation of the (PBP)M fragment is close to constant.

The hydride ligand in 1RhN, 1RhNq, 1IrCq, 3RhN, and 3IrC is close to being *trans* to either C or N of the pyridyl (161°–174° angle range). In the structure of 2RhC, the C–Rh–Br angle deviates from linearity to a greater extent (149.57(11)°) and 2RhC can be viewed as adopting a Y-shaped geometry as opposed to square-pyramidal for the five-coordinate hydride complexes 1RhN, 1RhNq, 1IrCq.^28^

### DFT studies

**Fig. 4:**
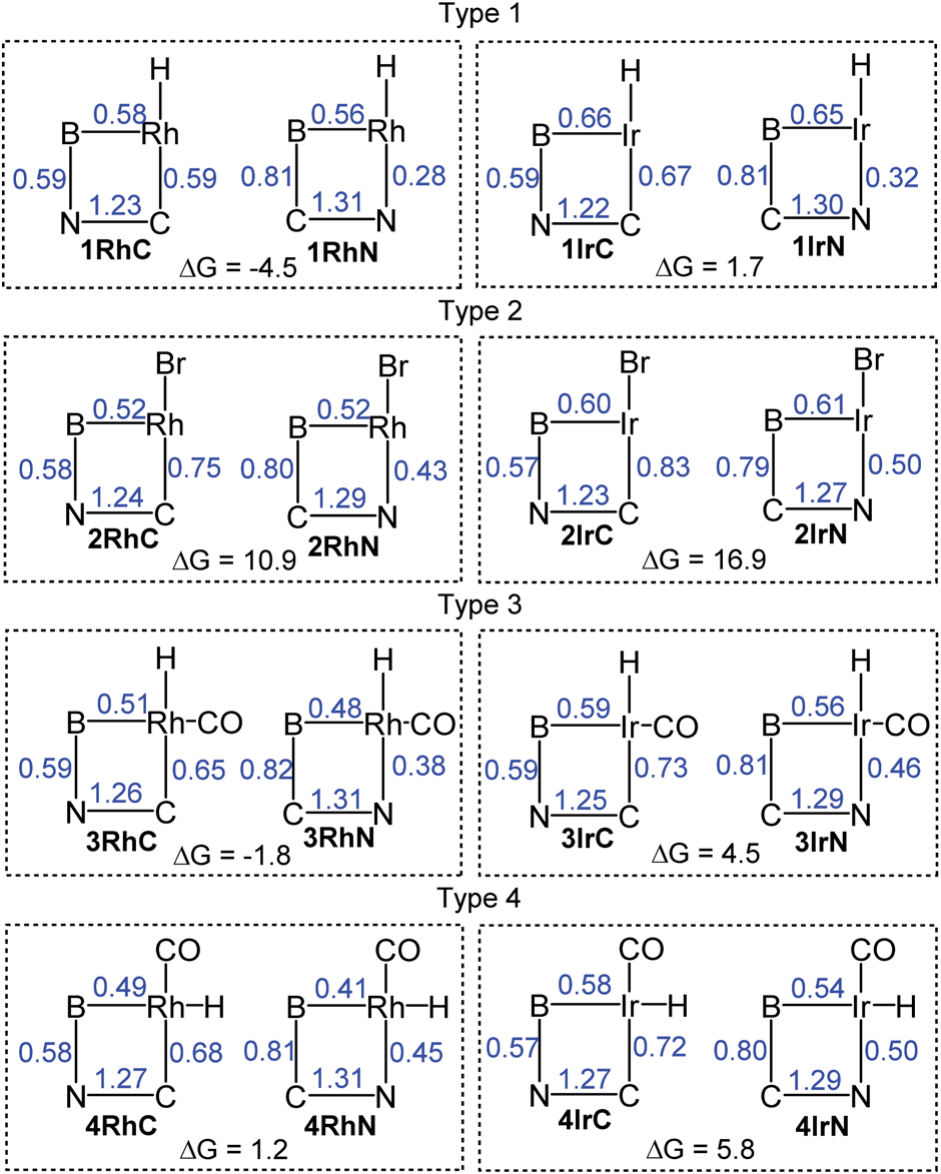
DFT-calculated Wiberg bond indices shown in blue for the bonds within the four-membered metallacycle. Δ*G*_298_ values (in kcal mol^−1^) are given for the isomerization from left to right within each box (negative Δ*G* value favors the isomer with the M–N bond).

The structures of the 16 molecules shown in Fig. 2 were optimized using the B97D3/LANL2DZ/6-31G(d) method (see details in the ESI†). Fig. 4 summarizes the results of the calculations, showing the Wiberg bond indices (WBI) within the four-membered rings, as well as the calculated free energies of the isomerization from the M–C to the M–N isomer. The metric details of the DFT-optimized geometries matched those from the XRD structures reasonably well.

Calculations indicate that the isomers with the carbon bound to the transition metal are more favorable for all Ir complexes and for the Rh complexes of types 2 and 4. For the other Rh complexes, the isomer with the nitrogen bound to Rh is preferred. Across all four types, the relative free energy preference of Ir for the metal–carbon bonded isomer is very consistently 5–7 kcal mol^−1^ higher than that of Rh.

Overall, the calculated thermodynamic parameters are consistent with the experimental observations we have for the Rh and Ir compounds of types 1–3. Moreover, the calculated free energy preferences for 1IrC (over 1IrN) and for 3RhN (over 3RhC) are <2 kcal mol^−1^, suggesting that both isomers in these two pairs should be present at observable concentrations. This is precisely what we observed for 1IrCq/1IrNq and for 3RhN/3RhC (*vide supra*), with the isomer predicted to be more favorable by DFT present in a higher proportion.

Type 3 (CO *trans* to B) compounds are isomeric to type 4 (H *trans* to B), and DFT calculations predict that any of the four type 3 compounds (3IrC, 3IrN, 3RhC, 3RhN) is lower in free energy than their corresponding type 4 analog (4IrC, 4IrN, 4RhC, 4RhN, respectively) by 13–19 kcal mol^−1^. This is consistent with the lack of observation of 4RhC or 4RhN in the thermolysis of the 3RhC/3RhN mixture.

The calculated Wiberg bond indices (WBI) provide a way to analyze the changes in the nature of the bonds in the four-membered cycle for the pairs of isomers. The WBI for the M–B bond in any Ir compound is 0.08–0.13 higher than for the exact Rh analog. Higher WBI values in Ir (*vs.* Rh) compounds are also notable for the M–C and M–N bonds (by 0.04–0.08). This is in general expected for a 5d metal (Ir) compared to its 4d congener (Rh).

Within each M–C/M–N isomeric pair with the same metal, the M–B bond WBI values differ only by 0.04 or less, except for the 4RhC/4RhN pair (0.08 difference). The WBI vary even less for the C–B bonds (0.79–0.82 range) and for the N–B bonds (0.57–0.59) throughout the whole array of compounds. It can be concluded that the changes in the M–B, C–B, and N–B bonding contribute little to the thermodynamic preferences for the M–C *vs.* M–N isomers.

The WBI values for the CN bond vary within a range of 1.22–1.31 for all 16 compounds. Within every M–C/M–N isomeric pair, this value is higher for the N–M bound isomer, by 0.02–0.08, suggesting that coordination to Ir or Rh strengthens the C–N bond slightly, but to a similar degree across all four types of compounds.

**Fig. 5:**
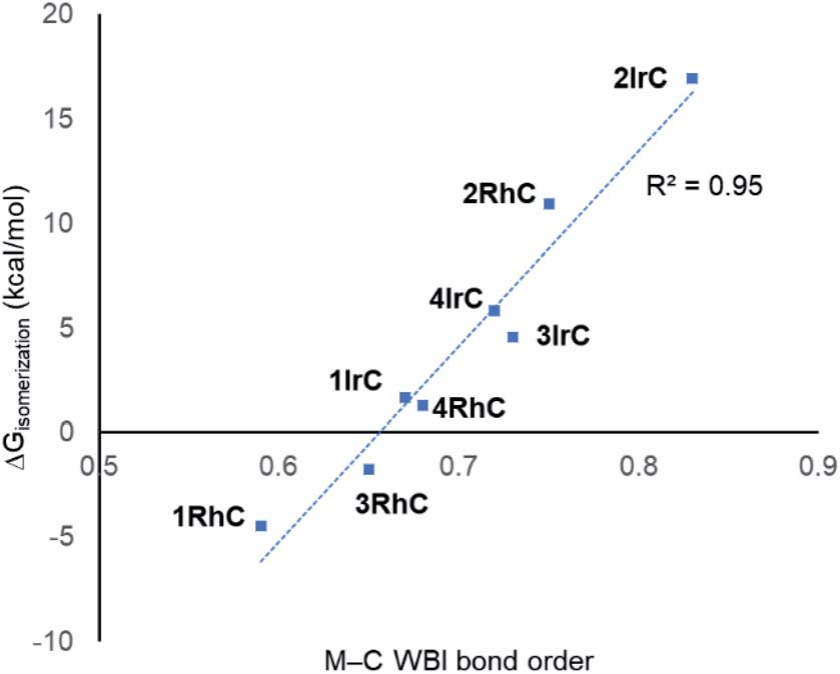
Correlation between the free energy of isomerization and the M–C WBI values for compounds under study.

Considering the M–C bonds, there appears to be a surprisingly linear correlation (Fig. 5) between the WBI values and the thermodynamic isomeric preference, that covers both the Rh and the Ir examples. Higher M–C WBI corresponds to higher preference for the M–C isomer, with ergoneutrality of the isomerization predicted at *ca.* 0.65 M–C WBI. The WBI values of the M–N bonds trend in the same direction. However, the correlation is more diffuse and not as steep, likely reflecting the intrinsically weaker nature of the M–N bond and its lesser dependence on the environment about the metal center (see Fig. S5†).

These observations lead us to conclude that the main factor controlling the thermodynamics of the M–C/M–N isomerization is the quality of the M–C bond, or in other words, the capacity of the metal site for making the strongest M–C bond. This capacity is always greater for Ir than for Rh, but it is also strongly influenced by the nature of the ligand *trans* to C. A hydride *trans* to C (types 1 and 3) is a maximal *trans*-influence conflict, leading to the weakest M–C bonds. A bromide *trans* to C (type 2) is much less *trans*-influencing than a hydride, leading to the strongest M–C bonds. A carbonyl ligand *trans* to C (type 4) represents an intermediate situation. Type 3 can be viewed as type 1 with additional CO ligand coordinated; apparently, CO coordination increases the M–C bond strength and therefore the preference for the M–C bound isomer. Notably, the WBI for the Rh–C bond in 2RhC is higher than the WBI values for all the Ir complexes except 2IrC, meaning that the weak *trans*-influence of Br (*vs.* H or CO) can strengthen the M–C bond *trans* to it to a degree that can overcome the 4d/5d metal handicap.

We have also analyzed the bonding using the extended-transition-state natural orbitals for chemical valence (ETS-NOCV) partitioning of the interaction of the closed shell 2-pyridyl anionic fragment with the formally cationic (PBP)Ir framework (see details in the ESI†). The findings dovetailed the WBI analysis: greater energy of interaction was calculated for (1) Ir *vs.* Rh, (2) M–C *vs.* M–N isomers, and (3) for type 2 *vs.* the other types.

**Fig. 6:**
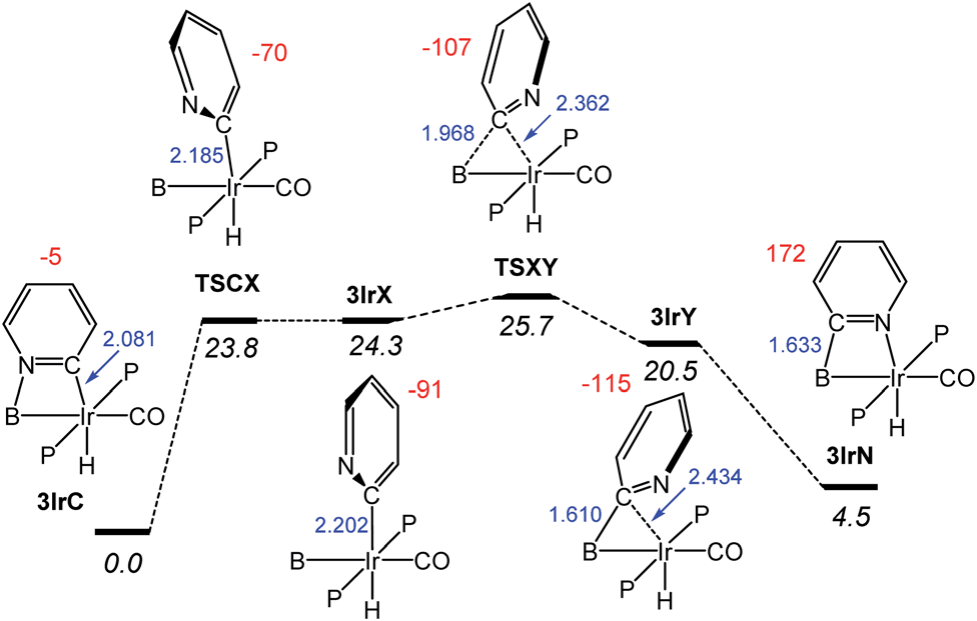
Calculated reaction coordinate for the isomerization of 3IrC into 3IrN. The relative free energy values in kcal mol^−1^ are given in italics. The numbers in blue (in Å) show the B–C and Ir–C distances while the numbers in red show the values (in °) for the dihedral angles N–C–Ir–B.

Next, we examined the mechanism^29^ of the interconversion between 3IrC and 3IrN as a representative example (Fig. 6). From 3IrC, the reaction proceeds *via* dissociation of the pyridine N from B with concomitant *ca.* 90° rotation about the Ir–C bond, resulting in 3IrX. The structure of the intermediate 3IrX evinces no bonding interactions between the pyridyl fragment and B, but a full-fledged Ir–C bond. The transition state connecting it with 3IrC (TSCX) possesses both a similar energy and geometry, with an incomplete rotation. The migration of the pyridyl from Ir in 3IrX to B in intermediate 3IrY proceeds *via*TSXY. In 3IrY, the pyridyl C is connected to the B by means of well-developed C–B bond, which is even 0.023 Å shorter than the calculated C–B distance in 3IrN. The pyridyl C in 3IrY can also be viewed as weakly interacting with Ir. We did not locate a transition state for the conversion of 3IrY into 3IrN; this process is also simply a rotation of the pyridyl with coordination to Ir. It is clear that most of the barrier for the interconversion between 3IrC and 3IrN is owing to the dissociation of N from B/Ir, corresponding to the rotation of N away from B/Ir. Once the pyridyl N is free, the barrier for the migration of the C-pyridyl between B and Ir is only a few kcal mol^−1^. This likely also applies to the other types presented in this paper.

## Conclusion

In summary, we have examined an unusual isomerization of a bridging 2-pyridyl unit in an array of Rh and Ir complexes supported by a PBP pincer ligand. The main factor governing the thermodynamic preference appears to be the strength of the M–C bond in the M–C bonded isomer. It was observed that the thermodynamic preference for the M–N *vs.* M–C bond depends both on the nature of the metal center and on the nature of the ligand *trans* to the M–N/M–C bond. The M–C isomer is favored for the 5d metal Ir *vs.* Rh and by the presence of a more weakly *trans*-influencing ligand *trans* to the M–N/M–C bond. For some of the complexes, both isomers were observed experimentally, in close agreement with theoretical analysis. The interconversion between isomers of similar thermodynamic stability appears to be easily accessible on the experimental timescale, consistent with the computational analysis of a representative system. These findings suggest that the possibility of M–C/M–N isomerization of 2-pyridyl and other closely related fragments should be taken into account when investigating C–H bond activation in azines using a combination of a late transition metal and an embedded main group Lewis acid.

## Data availability

Crystallographic information associated with this publication has been deposited and is available from https://www.ccdc.cam.ac.uk/ under CCDC 2014200, 2014201, 2014203–2014205.

## Author contributions

Y. C., J. Z., and O. V. O. conceived of the project, Y. C. and W.-C. S. performed the synthetic and spectroscopic characterization work, Y. C. and N. B. carried out the X-ray diffractions studies, J. Z. performed the DFT calculations, Y. C. and O. V. O. performed the bulk of the manuscript writing with input from the other co-authors, and J. Z. and O. V. O. supervised the overall direction of the work.

## Conflicts of interest

There are no conflicts to declare.

## Supplementary Material

SC-012-D1SC01850G-s001

SC-012-D1SC01850G-s002

SC-012-D1SC01850G-s003

SC-012-D1SC01850G-s004

SC-012-D1SC01850G-s005

SC-012-D1SC01850G-s006

SC-012-D1SC01850G-s007

SC-012-D1SC01850G-s008

SC-012-D1SC01850G-s009

SC-012-D1SC01850G-s010

SC-012-D1SC01850G-s011

SC-012-D1SC01850G-s012

SC-012-D1SC01850G-s013

SC-012-D1SC01850G-s014

SC-012-D1SC01850G-s015

SC-012-D1SC01850G-s016

SC-012-D1SC01850G-s017

SC-012-D1SC01850G-s018

SC-012-D1SC01850G-s019

SC-012-D1SC01850G-s020

SC-012-D1SC01850G-s021

SC-012-D1SC01850G-s022
