# Correction: Cooperative C–H activation of pyridine by PBP complexes of Rh and Ir can lead to bridging 2-pyridyls with different connectivity to the B–M unit

**DOI:** 10.1039/d2sc90123d

**Published:** 2022-06-28

**Authors:** Yihan Cao, Wei-Chun Shih, Nattamai Bhuvanesh, Jia Zhou, Oleg V. Ozerov

**Affiliations:** Department of Chemistry, Texas A&M University 3255 TAMU College Station Texas 77842 USA ozerov@chem.tamu.edu; State Key Laboratory of Urban Water Resource and Environment, Harbin Institute of Technology Harbin 150090 China jiazhou@hit.edu.cn

## Abstract

Correction for ‘Cooperative C–H activation of pyridine by PBP complexes of Rh and Ir can lead to bridging 2-pyridyls with different connectivity to the B–M unit’ by Yihan Cao *et al.*, *Chem. Sci.*, 2021, **12**, 14167–14173, https://doi.org/10.1039/D1SC01850G.

The authors regret that the incorrect US National Science Foundation grant number (grant CHE-2102095) was provided in the Acknowledgements section. The correct grant number is CHE-2102324.

The Royal Society of Chemistry apologises for these errors and any consequent inconvenience to authors and readers.

## Supplementary Material

