# Supplementary material for: Cooperative C–H activation of pyridine by PBP complexes of Rh and Ir can lead to bridging 2-pyridyls with different connectivity to the B–M unit
Source: Chem Sci. 2021 Oct 5;12(42):14167–73. doi: 10.1039/d1sc01850g (PMC8565379; doi:10.1039/d1sc01850g)
Supplement: SC-012-D1SC01850G-s021 [file SC-012-D1SC01850G-s021.pdf]

Electronic Supplementary Material (ESI) for Chemical Science.
This journal is © The Royal Society of Chemistry 2021

# Supporting Information

## Cooperative C-H Activation of Pyridine by PBP Complexes of Rh and Ir Can Lead to Bridging 2-Pyridyls with Different Connectivity to the B-M Unit

Yihan Cao[a], Wei-Chun Shih[a], Nattamai Bhuvanesh[a], Jia Zhou[b]* and Oleg V. Ozerov[a]*

[a] Department of Chemistry, Texas A&M University, 3255 TAMU, College Station, TX 77842.

[b] State Key Laboratory of Urban Water Resource and Environment, Harbin Institute of Technology, Harbin 150090, China.

ozerov@chem.tamu.edu, jiazhou@hit.edu.cn

**Table of Contents.**


**1. General Considerations.** **S3**

**2. Synthesis and Characterization of Rhodium and Iridium Complexes.** **S4**

**3. X-Ray Structural Determination Details.** **S16**

**4. DFT Calculations.** **S22**

**5. NMR Spectra.** **S32**

**6. SI References.** **S63**

## 1. General Considerations.

**General Considerations.** Unless specified otherwise, all manipulations were performed under an Ar atmosphere using standard Schlenk line or glovebox techniques. Toluene and pentane were dried and deoxygenated (by purging) using a solvent purification system (Innovative Technology Pure Solv MD-5 Solvent Purification System) and stored over molecular sieves in an Ar-filled glove box. $C_6D_6$ and $CDCl_3$ was dried over NaK/$Ph_2CO$/18-crown-6, distilled and stored over molecular sieves in an Ar-filled glovebox. Pyridine derivatives and cyclohexene were dried over $CaH_2$, vacuum transferred and stored over molecular sieves in an Ar-filled glove box. (PBP)RhHCl (**5**) and (PBP)IrHCl (**6**) were prepared via literature procedures.[1] All other chemicals were used as received from commercial vendors.

**Physical Methods.** NMR spectra were recorded on Mercury 300 ($^{1}$H NMR, 299.952 MHz; $^{31}$P NMR, 121.422 MHz), Bruker 400MHz ($^{11}$B NMR, 128.185 MHz, $^{1}$H NMR, 399.535 MHz; $^{31}$P NMR, 161.734 MHz) and Varian Inova 500 ($^{1}$H NMR, 499.703 MHz; $^{13}$C NMR, 125.697 MHz; $^{31}$P NMR, 202.265 MHz) spectrometer. Chemical shifts are reported in δ (ppm). For $^{1}$H and $^{13}$C NMR spectra, the residual solvent peak was used as an internal reference ($^{1}$H NMR: δ 7.16 for $C_6D_6$, δ 7.26 for $CDCl_3$, $^{13}$C NMR: δ 128.06 for $C_6D_6$, δ 77.2 for $CDCl_3$). $^{11}$B NMR spectra were referenced externally with $BF_3$ etherate at δ 0. $^{31}$P NMR spectra were referenced externally with 85% phosphoric acid at δ 0. FT-IR spectra were recorded on an Agilent Cary 630 ATR-IR spectrometer in a glovebox. Elemental analyses were performed by CALI Labs, Inc. (Highland Park, NJ)

## 2. Synthesis and Characterization of Rhodium and Iridium Complexes.

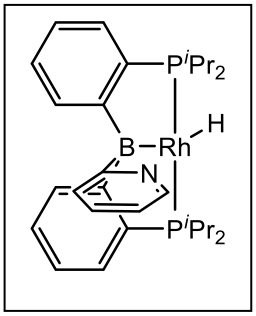


**($PB^{Py}P$)Rh(H) (1RhN).** In a 50 mL PTFE screw-capped reaction tube, $NaEt_3BH$ (0.40 mL, 0.40 mmol, 1.0 M in toluene) was added to a toluene solution (5 mL) of **5** (0.22 g, 0.40 mmol). The solution changed color from orange to dark red immediately. After stirring at room temperature for 5 min, a $^{31}P\{^1H\}$ NMR spectrum was recorded, showing two or more complexes formed (**Figure S1**). Volatiles were removed under vacuum to remove $BEt_3$ that was generated. The resulting red solid was directly dissolved in toluene (10 mL), to which were added pyridine (35 μL, 0.44 mmol) and cyclohexene (44 μL, 0.44 mmol). The reaction mixture was stirred at 80 °C for 12 h, and gradually changed from dark-red to brown color. The solution was filtered through Celite, and volatiles were removed under vacuum. The resulting brown solid was recrystallized in toluene/pentane (1:3), yielding a pale-yellow solid (163 mg, 70%).

$^1H$ NMR (300 MHz, $C_6D_6$): δ 8.46 (d, $J_{H–H}$ = 7.5 Hz, 2H, $PB^{Py}P$-*Phenyl*), 8.34 (d, $J_{H–H}$ = 4.8 Hz, 1H, $PB^{Py}P$-*Pyridyl*), 7.31 (t, $J_{H–H}$ = 7.2 Hz, 2H, $PB^{Py}P$-*Phenyl*), 7.18 (m, 4H, $PB^{Py}P$-*Phenyl*), 6.68 (m, 2H, $PB^{Py}P$-*Pyridyl*), 6.45(m, 1H, $PB^{Py}P$-*Pyridyl*), 2.29 (m, 2H, C*H*$Me_2$), 1.92 (m, 2H, C*H*$Me_2$), 1.32 (dvt, $J_{H–H}$ = $J_{H–P}$ = 7.6 Hz, 6H, CH*$Me_2$*), 1.12 (dvt, $J_{H–H}$ = $J_{H–P}$ = 7.0 Hz, 6H, CH*$Me_2$*), 1.04 (dvt, $J_{H–H}$ = $J_{H–P}$ = 7.4 Hz, 6H, CH*$Me_2$*), 0.57 (dvt, $J_{H–H}$ = $J_{H–P}$ = 7.1 Hz, 6H, CH*$Me_2$*), -17.25 (dt, $J_{H–P}$ = 18.5 Hz, $J_{H–Rh}$ = 22.9 Hz, 1H, Rh–*H*). $^{31}P\{^1H\}$ NMR (121 MHz, $C_6D_6$): δ 60.6 (d, $J_{P–Rh}$ = 141.4 Hz). $^{11}B\{^1H\}$ NMR (128 MHz, $C_6D_6$): δ 3.5. $^{13}C\{^1H\}$ NMR (126 MHz, $C_6D_6$) δ 188.2 (br, Rh–B–*C*–N), 165.4 (br, B–*Phenyl*), 149.6 (s, *Pyridyl*), 137.9 (td, $J_{P–C}$ = 19.8 Hz, $J_{Rh–C}$ = 3.5 Hz, P-*Phenyl*), 132.8 (s, *Pyridyl*), 129.9 (t, $J_{P–C}$ = 9.9 Hz, *Phenyl*), 129.2 (s, *Phenyl*), 125.1 (t, $J_{P–C}$ = 3.1 Hz, *Phenyl*), 122.2 (s, *Pyridyl*), 121.0 (s, *Pyridyl*), 30.0 (t, $J_{P–C}$ = 8.3 Hz, *C*H$Me_2$), 27.7 (t, $J_{P–C}$ = 14.5 Hz, *C*H$Me_2$), 21.3 (t, $J_{P–C}$ = 4.8 Hz, CH*$Me_2$*), 20.4 (t, $J_{P–C}$ = 2.1 Hz, CH*$Me_2$*), 20.1 (s, CH*$Me_2$*),

20.0 (t, $J_{P\text{–}C}$ = 3.6 Hz, CH*Me*$_2$). Anal. Calc. for $C_{29}H_{41}BNP_2Rh$: C, 60.13; H, 7.13; N, 2.42. Found: C, 60.25; H, 6.97; N, 2.34.

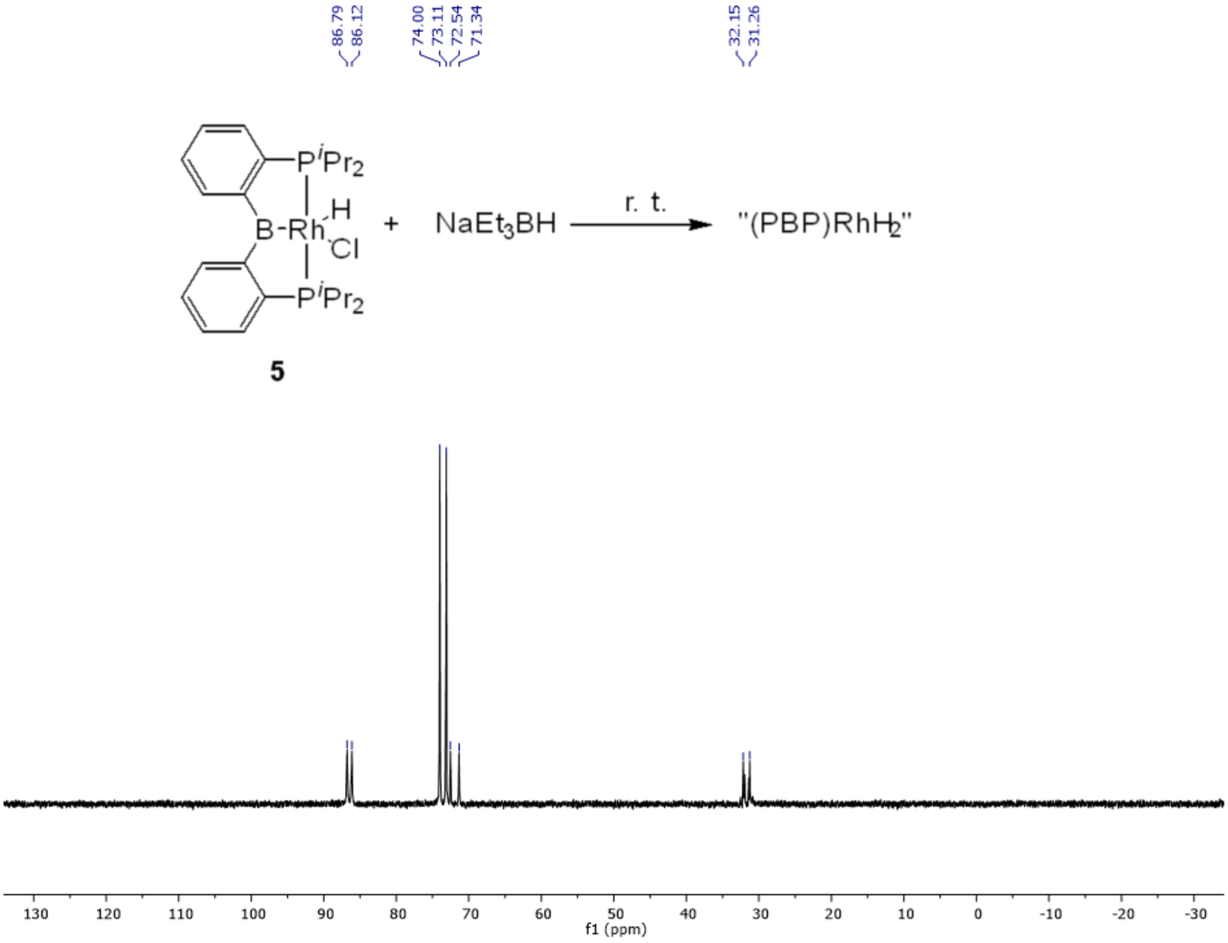


**Figure S1.** $^{31}P\{^1H\}$ NMR spectrum record on a 500 MHz Varian NMR after 1 equiv. of $NaEt_3BH$ were added to **5** in toluene, showing four or more complexes formed.

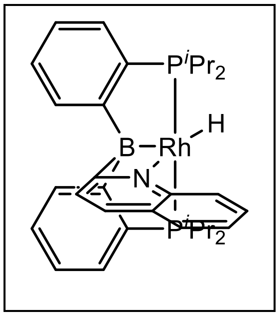


**(PB$^{Qu}$P)Rh(H) (1RhNq).** In a 50 mL PTFE screw-capped reaction tube, $NaEt_3BH$ (0.52 mL, 0.52 mmol, 1.0 M in toluene) was added to a toluene solution (5 mL) of **5** (0.27 g, 0.50 mmol). The solution changed color from orange to dark-red immediately. After stirring at room temperature for 5 min, volatiles were removed under vacuum to remove $BEt_3$ that was

generated. The resulting red solid was dissolved in toluene (10 mL), to which were added quinoline (62 μL, 0.52 mmol) and cyclohexene (53 μL, 0.52 mmol). The reaction mixture was stirred at 80 °C for 12 h, and gradually changed from dark-red to brown color. The solution was filtered through Celite, and volatiles were removed under vacuum. The resulting brown solid was recrystallized in toluene/pentane 1:3, yielding a pale-yellow solid (0.19 g, 59%).

$^{1}$H NMR (400 MHz, $C_6D_6$): δ 8.52 (d, $J_{H–H}$ = 7.5 Hz, 2H, $PB^{Py}P$-*Phenyl*), 8.08 (d, $J_{H–H}$ = 8.3 Hz, 1H, $PB^{Qu}P$-*Quinolyl*), 7.39 (t, $J_{H–H}$ = 7.4 Hz, 1H, $PB^{Qu}P$-*Quinolyl*), 7.33 (t, $J_{H–H}$ = 7.0 Hz, 2H, $PB^{Py}P$-*Phenyl*), 7.15 (m, 6H, $PB^{Py}P$-*Phenyl & Quinolyl*), 7.03 (t, $J_{H–H}$ = 7.4 Hz, 1H, $PB^{Qu}P$-*Quinolyl*), 6.94 (d, $J_{H–H}$ = 8.2 Hz, 1H, $PB^{Qu}P$-*Quinolyl*), 2.24 (m, 2H, C*H*$Me_2$), 1.84 (m, 2H, C*H*$Me_2$), 1.37 (dvt, $J_{H–H}$ = $J_{H–P}$ = 7.5 Hz, 6H, CH*$Me_2$*), 1.06 (m, 12H, CH*$Me_2$*), 0.19 (dvt, $J_{H–H}$ = $J_{H–P}$ = 6.9 Hz, 6H, CH*$Me_2$*), -16.81 (dt, $J_{H–P}$ = 18.5 Hz, $J_{H–Rh}$ = 23.2 Hz, 1H, Rh–*H*). $^{31}$P{$^{1}$H} NMR (121 MHz, $C_6D_6$): δ 60.3 (d, $J_{P–Rh}$ = 141.1 Hz). $^{11}$B{$^{1}$H} NMR (128 MHz, $C_6D_6$): δ 4.5. $^{13}$C{$^{1}$H} NMR (126 MHz, $C_6D_6$): δ 189.5 (br, Rh–B–*C*–N), 164.8 (br, B–*Phenyl*), 148.5 (s, *Quinolyl*), 138.3 (td, $J_{P–C}$ = 19.5 Hz, $J_{Rh–C}$ = 3.7 Hz, P-*Phenyl*), 132.2 (s, *Quinolyl*), 129.8 (t, $J_{P–C}$ = 9.7 Hz, *Phenyl*), 129.4 (s, *Phenyl*), 128.7 (s, *Quinolyl*), 128.6 (s, *Quinolyl*), 128.5(s, *Quinolyl*), 125.3 (t, $J_{P–C}$ = 3.3 Hz, *Phenyl*), 124.9 (s, *Quinolyl*), 121.3 (s, *Quinolyl*), 29.8 (t, $J_{P–C}$ = 8.4 Hz, *C*H$Me_2$), 28.6 (t, $J_{P–C}$ = 14.6 Hz, *C*H$Me_2$), 21.6 (t, $J_{P–C}$ = 4.6 Hz, CH*$Me_2$*), 20.3 (t, $J_{P–C}$ = 4.2 Hz, CH*$Me_2$*), 20.1 (s, CH*$Me_2$*), 19.8 (s, CH*$Me_2$*). Anal. Calc. for $C_{33}H_{43}BNP_2Rh$: C, 62.98; H, 6.89; N, 2.23. Found: C, 62.35; H, 6.59; N, 2.25.

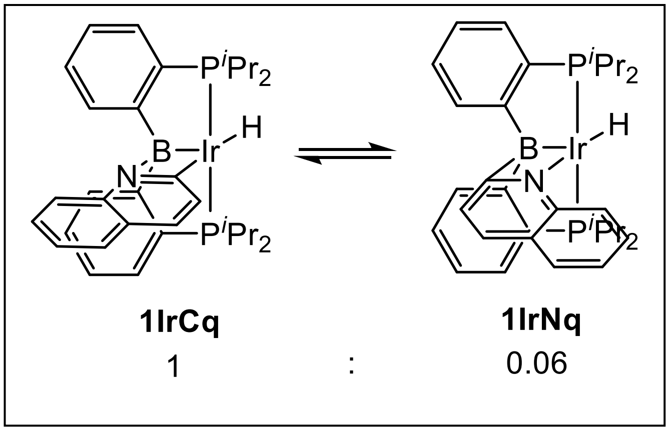


**Synthesis of (PBP)Ir(Qu)H/(PB$^{Qu}$P)IrH (1IrCq/1IrNq). 6** (0.30 g, 0.48 mmol) and quinoline (63 μL, 0.53 mmol) was dissolved in 2 mL toluene, to which was added sodium bis(trimethylsilyl)amide (88 mg, 0.48 mmol). The reaction mixture was stirred at r. t. for 1 h, and passed through Celite. Volatiles were removed under vacuum, and the resulting solid was recrystallized in toluene/pentane 1:10, yielding an orange solid (0.25 g, 72%). The resulting product exists as an equilibrium in solution, and the ratio was calculated to be **1IrCq**/**1IrNq** = 1/0.06 by integration of the most downfield PBP-*Phenyl* signal in $^1$H NMR spectrum. NMR spectroscopic data for **1IrCq**: $^1$H NMR (500 MHz, $C_6D_6$) δ 8.80 (d, $J_{H–H}$ = 7.6 Hz, 2H, PBP-*Phenyl*), 8.19 (d, $J_{H–H}$ = 8.4 Hz, 1H, *Quinolyl*), 8.00 (d, $J_{H–H}$ = 8.5 Hz, 1H, *Quinolyl*), 7.24 (t, $J_{H–H}$ = 7.3 Hz, 2H, PBP-*Phenyl*), 7.20 (m, 2H, PBP-*Phenyl*), 7.10 (m, 5H, PBP-*Phenyl & Quinolyl*), 6.78 (t, $J_{H–H}$ 7.4 Hz, 1H, *Quinolyl*), 2.47 (m, 2H, C*H*Me$_2$), 2.17 (m, 2H, C*H*Me$_2$), 1.36 (dvt, $J_{H–H}$ = $J_{H–P}$ = 7.5 Hz, 6H, CH*Me*$_2$), 1.14 (dvt, $J_{H–H}$ = $J_{H–P}$ = 7.2 Hz, 6H, CH*Me*$_2$), 1.03 (dvt, $J_{H–H}$ = $J_{H–P}$ = 7.1 Hz, 6H, CH*Me*$_2$), 0.61 (dvt, $J_{H–H}$ = $J_{H–P}$ = 7.0 Hz, 6H, CH*Me*$_2$), -0.20 (t, $J_{H–P}$ = 23.2 Hz, 1H, Ir–H). $^{31}$P NMR (202 MHz, $C_6D_6$, with selected range of $^1$H decoupled) δ 59.0 (s). $^{11}$B{$^1$H} NMR (128 MHz, $C_6D_6$) δ -8.5. $^{13}$C{$^1$H} NMR (126 MHz, $C_6D_6$) δ 201.3 (t, $J_{P–C}$ = 6.2 Hz, Ir–*C*), 159.0 (br, B–*Phenyl*), 141.6 (s, *Quinolyl*), 141.0 (t, $J_{P–C}$ = 23.9 Hz, P–*Phenyl*), 135.6 (s, *Quinolyl*), 130.2 (s, *Phenyl*), 130.1 (s, *Quinolyl*), 129.8 (t, $J_{P–C}$ = 8.2 Hz, *Phenyl*), 129.5 (s, *Quinolyl*), 129.0 (s,

*Quinolyl*), 126.0 (t, $J_{P–C}$ = 3.6 Hz, *Phenyl*), 127.7 (s, *Phenyl*), 124.1 (s, *Quinolyl*), 122.9 (s, *Quinolyl*), 117.7 (s, *Quinolyl*), 30.5 (t, $J_{P–C}$ = 11.7 Hz, *C*HMe$_2$), 29.0 (t, $J_{P–C}$ = 18.1 Hz, *C*HMe$_2$), 20.6 (s, CH*Me*$_2$), 20.5 (t, $J_{P–C}$ = 3.5 Hz, CH*Me*$_2$), 20.3 (m, CH*Me*$_2$), 19.60 (s, CH*Me*$_2$). Selected NMR spectroscopic data for **1IrNq**. $^1$H NMR (500 MHz, $C_6D_6$) δ 8.64 (d, $J_{H–H}$ = 7.6 Hz, 2H, PBP-*Phenyl*), 8.14 (d, $J_{H–H}$ = 7.8 Hz, 1H, Quinolyl), 6.84 (d, $J_{H–H}$ = 8.4 Hz, 2H, PBP-*Phenyl*), 2.00 (m, 2H, C*H*Me$_2$), 0.19 (dvt, $J_{H–H}$ = $J_{H–P}$ = 7.1 Hz, 6H, CH*Me*$_2$), -17.10 (t, $J_{P–H}$ = 19.5 Hz, Ir–H). $^{31}$P NMR (202 MHz, $C_6D_6$, with selected range of $^1$H decoupled) δ 59.0 (d, $J_{P–H}$ = 19.3 Hz, Ir–H). Anal. Calc. for $C_{33}H_{43}BIrNP_2$: C, 55.15; H, 6.03; N, 1.95. Found: C, 55.21; H, 6.23; N, 2.01.

**VT-NMR experiment of 1IrCq/1IrNq in $C_6D_6$.** In a J. Young tube, **1IrCq/1IrNq** was dissolved in 0.4 mL $C_6D_6$. $^1$H NMR were recorded at different temperature using a 500 MHz Varian NMR. **1IrCq/1IrNq** exists as an equilibrium in solution, and the ratio was calculated by integration of the most downfield PBP-*Phenyl* signal in $^1$H NMR spectrum. (**Figure S2**)

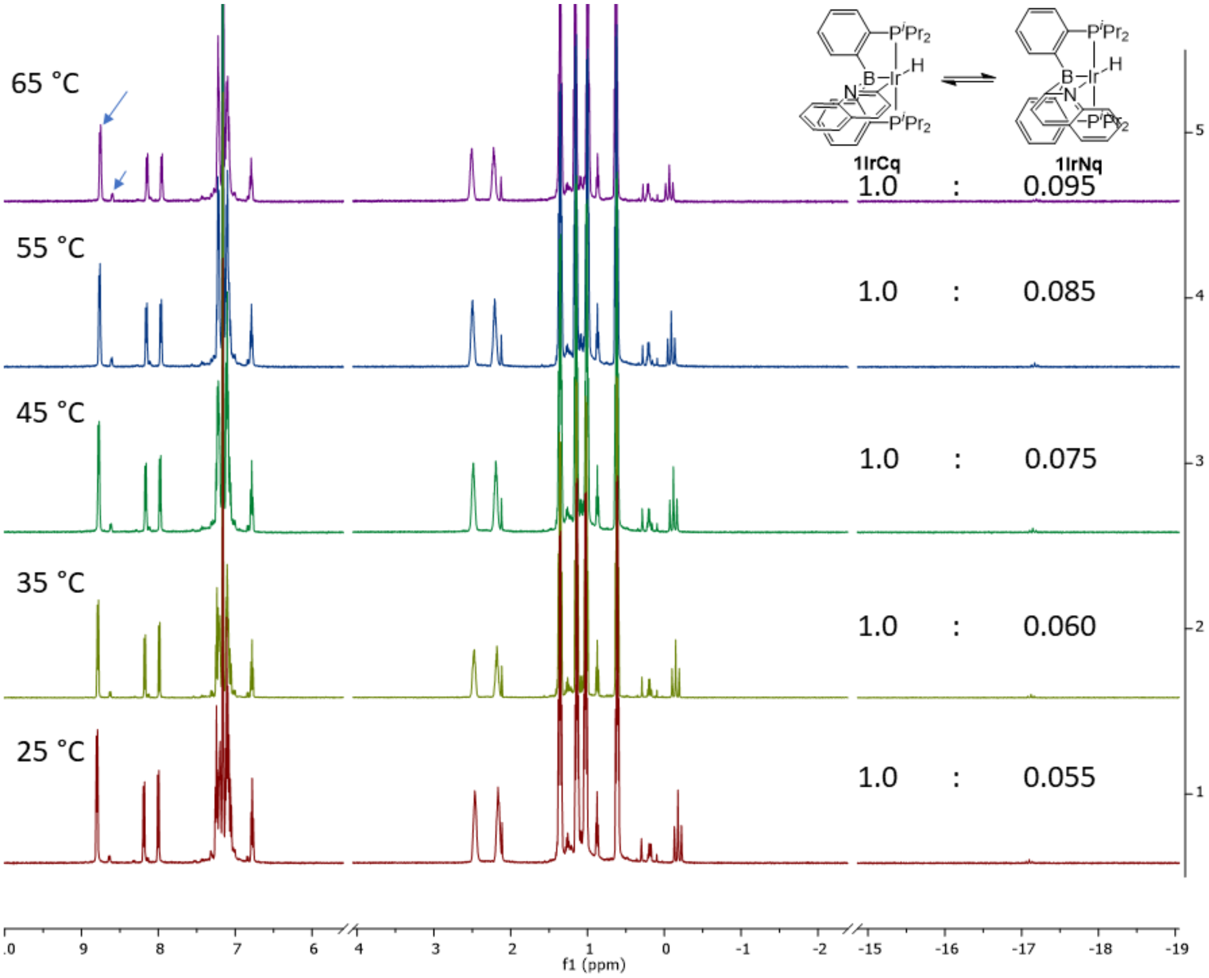


**Figure S2.** VT-NMR experiment of **1IrCq/1IrNq** on a 500 MHz Varian NMR.

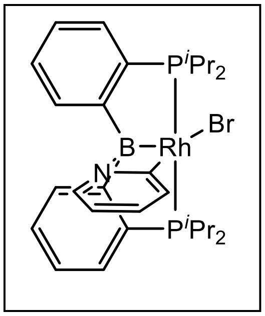


**(PBP)RhPyBr (2RhC).** In a J. Young tube, N-bromosuccinimide (0.17 mmol, 30 mg) was added to a solution of **1RhN** (100 mg, 0.17 mmol) in 0.40 mL $C_6D_6$. This reaction mixture was stirred at room temperature for 24 h and heated at 100 °C for 12 h until all **1RhN** were consumed. (monitored by $^{31}P\{^1H\}$ NMR). The resulting mixture was filtered over a pad of silica gel and washed through with dichloromethane. All volatiles were removed under vacuum and the resulting solid was recrystallized from toluene/pentane (1:3), yield an orange solid (80 mg, 70%)

$^{1}H$ NMR (500 MHz, $C_6D_6$) δ 8.44 (d, $J_{H–H}$ = 7.5 Hz, 2H, PBP-*Phenyl*), 7.61 (d, $J_{H–H}$ = 8.2 Hz, 1H, *Pyridyl*), 7.32 (t, $J_{H–H}$ = 6.8 Hz, 2H, PBP-*Phenyl*), 7.13 (m, 4H, PBP-*Phenyl*), 6.62 (d, $J_{H–H}$ = 5.6 Hz, 1H, *Pyridyl*), 6.09 (t, $J_{H–H}$ = 7.7 Hz, 1H, *Pyridyl*), 5.59 (t, $J_{H–H}$ = 6.5 Hz, 1H, *Pyridyl*), 3.56 (m, 2H, C*H*Me$_2$), 2.14 (m, 2H, C*H*Me$_2$), 1.56 (dvt, $J_{H–H}$ = $J_{H–P}$ = 7.3 Hz, 6H, CH*Me*$_2$), 1.15 (dvt, $J_{H–H}$ = $J_{H–P}$ = 7.5 Hz, 6H, CH*Me*$_2$), 1.08 (dvt, $J_{H–H}$ = $J_{H–P}$ = 6.5 Hz, 6H, CH*Me*$_2$), 0.49 (dvt, $J_{H–H}$ = $J_{H–P}$ = 7.5 Hz, 6H, CH*Me*$_2$). $^{31}P\{^{1}H\}$ NMR (202 MHz, $C_6D_6$): δ 42.8 (d, $J_{P–Rh}$ = 129.2 Hz). $^{11}B\{^{1}H\}$ NMR (128 MHz, $C_6D_6$): δ 1.7. $^{13}C\{^{1}H\}$ NMR (126 MHz, $C_6D_6$) δ 178.0 (dt, $J_{C–Rh}$ = 34.8 Hz, $J_{C–P}$ = 10.2 Hz, *C*–Rh), 160.1 (br, B–*Phenyl*), 138.5 (s, *Pyridyl*), 137.3 (td, $J_{P–C}$ = 19.0 Hz, $J_{Rh–C}$ = 3.4 Hz, P–*Phenyl*), 134.0 (s, *Pyridyl*), 131.8 (s, *Pyridyl*), 131.7 (s, *Phenyl*), 131.2 (t, $J_{P–C}$ = 9.1 Hz, *Phenyl*), 129.5 (s, *Phenyl*), 127.4 (t, $J_{P–C}$ = 3.2 Hz, *Phenyl*), 116.0 (s, *Pyridyl*), 27.6 (t, $J_{P–C}$ = 12.3 Hz, *C*HMe$_2$), 25.6 (t, $J_{P–C}$ = 10.3 Hz, *C*HMe$_2$), 20.6 (s, CH*Me*$_2$), 19.9 (t, $J_{P–C}$ = 2.3 Hz, CH*Me*$_2$), 19.3 (t, $J_{P–C}$ = 3.1 Hz, CH*Me*$_2$), 18.4 (s, CH*Me*$_2$). Anal. Calc. for $C_{29}H_{40}BBrNP_2Rh$: C, 52.92; H, 6.13; N, 2.13. Found: C, 52.40; H, 5.81; N, 1.85.

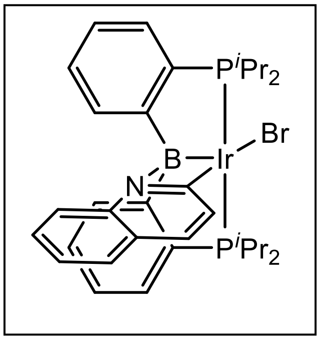


**(PBP)Ir(Qu)Br. (2IrCq).** To **1IrCq/1IrNq** (0.14 mmol, 100 mg) in 2 mL toluene was added N-bromosuccinimide (0.14 mmol, 25 mg). The reaction mixture was heated at 50 °C for 12 h. The resulting mixture was filtered over a pad of silica gel and washed through with dichloromethane. All volatiles were removed under vacuum and the resulting solid was recrystallized from THF/pentane (1:5), yield an orange solid (77 mg, 69%). $^{1}H$ NMR (500 MHz, $C_6D_6$) δ 8.86 (d, $J_{H–H}$ = 7.7 Hz, 2H, PBP-*Phenyl*), 7.67 (d, $J_{H–H}$ = 8.8 Hz, 1H, *Quinolyl*), 7.64 (d, $J_{H–H}$ = 8.3 Hz, 1H, *Quinolyl*), 7.30 (m, 2H, PBP-*Phenyl*), 7.07 (m, 4H, PBP-*Phenyl*), 6.89 (m, 2H, *Quinolyl*), 6.65 (t, $J_{H–H}$ = 7.5 Hz, 1H, *Quinolyl*), 6.52 (d, $J_{H–H}$ = 8.8 Hz, 1H, *Quinolyl*), 3.79 (m, 2H, C*H*Me$_2$), 2.50 (m, 2H, C*H*Me$_2$), 1.51 (dvt, $J_{H–H}$ = $J_{H–P}$ = 7.3 Hz, 6H,

CH*Me*$_2$), 1.10 (m, 12H, CH*Me*$_2$), 0.44 (dvt, $J_{H–H} = J_{H–P}$ = 7.5 Hz, 6H, CH*Me*$_2$). $^{31}P\{^1H\}$ NMR (202 MHz, $CDCl_3$) δ 40.0 (s). $^{11}B\{^1H\}$ NMR (128 MHz, $CDCl_3$) δ -6.8. $^{13}C\{^1H\}$ NMR (126 MHz,$CDCl_3$) δ 176.9 (s, Ir–C), 157.7 (br, B–*Phenyl*), 152.0 (t, $J_{C–P}$ = 6.2 Hz, *Quinolyl*), 141.0 (s, *Quinolyl*), 136.1 (t, $J_{C–P}$ = 24.4 Hz, P–*Phenyl*), 131.1 (t, $J_{C–P}$ = 1.8 Hz, *Phenyl*), 131.1 (s, *Quinolyl*), 130.3 (t, $J_{C–P}$ = 8.3 Hz, *Phenyl*), 129.8 (s, *Quinolyl*), 128.7 (s, *Quinolyl*), 128.4 (s, *Phenyl*), 126.3 (t, $J_{C–P}$ = 3.8 Hz, *Phenyl*), 123.7 (s, *Quinolyl*), 123.6 (s, *Quinolyl*), 117.0 (s, *Quinolyl*), 25.6 (t, $J_{C–P}$ = 15.9 Hz, *C*HMe$_2$), 25.0 (t, $J_{C–P}$ = 12.6 Hz, *C*HMe$_2$), 19.4 (s, CH*Me*$_2$), 18.9 (t, $J_{C–P}$ = 1.6 Hz, CH*Me*$_2$), 18.6 (t, $J_{C–P}$ = 2.8 Hz, CH*Me*$_2$), 17.9 (s, CH*Me*$_2$). Anal. Calc. for $C_{29}H_{40}BBrNP_2Ir$: C, 49.70; H, 5.31; N, 1.36. Found: C, 48.95; H, 5.06; N, 1.68.

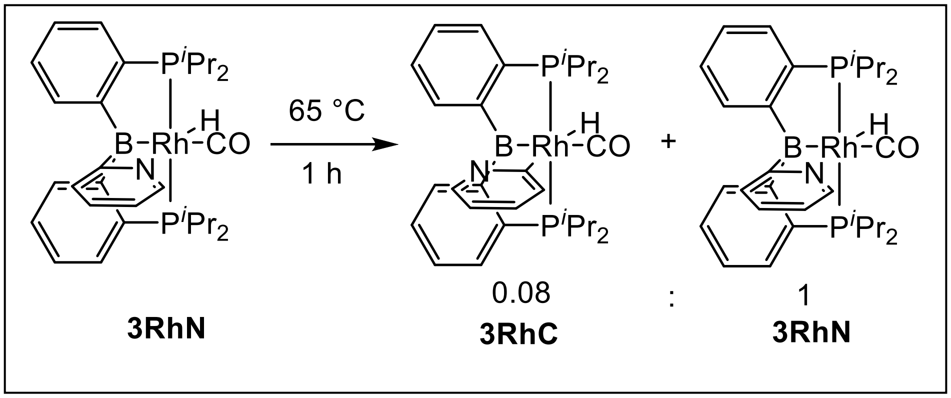


**In situ generation of (PB$^{Py}$P)RhH(CO) and observation of the mixture of (PB$^{Py}$P)RhH(CO)/(PBP)RhPyH(CO) after heating (3RhN/3RhC).** In a J. Young tube, 1 atm of CO was added to a solution of **1RhN** (40 mg, 0.086 mmol) in 0.40 mL $C_6D_6$ after 3 rounds of freeze-pump-thaw. After stirring at room temperature for 10 min, NMR spectra were recorded, showing the generation of **3RhN**. All volatiles were removed and 0.40 mL $C_6D_6$ was added. ATR-IR spectrum were recorded. The solution was heated at 65 °C for 1 h. $^1H$ NMR and $^{31}P\{^1H\}$ NMR spectra were recorded at room temperature after heating, showing a mixture of **3RhC**/**3RhN** (0.08:1). (calculated by integration of methyl signals in $^1H$ NMR spectrum). The mixture was

further heated at 65 °C for 24 h. It resulted in a mixture of four different compounds: an unidentified complex, **3RhC, 3RhN,** and **1RhN** (1:3:17:1). ($^{31}P\{^1H\}$ NMR evidence, **Figure S3**).

Spectroscopic data for **3RhN** under CO atmosphere**:** $^1H$ NMR (500 MHz, $C_6D_6$) δ 8.41 (d, $J_{H–H}$ = 7.6 Hz, 2H, PB$^{Py}$P-*Phenyl*), 7.90 (d, $J_{H–H}$ = 5.0 Hz, 1H, PB$^{Py}$P- *Pyridyl*), 7.36 (t, $J_{H–H}$ = 7.0 Hz, 2H, PB$^{Py}$P-*Phenyl*), 7.15 (m, 4H, PB$^{Py}$P-*Phenyl*), 6.67 (t, $J_{H–H}$ = 7.6 Hz, 1H, PB$^{Py}$P-*Pyridyl*), 6.58 (d, $J_{H–H}$ = 7.7 Hz, 1H, PB$^{Py}$P-*Pyridyl*), 6.28 (dd, $J_{H–H}$ = 7.2, 5.2 Hz, 1H, PB$^{Py}$P-*Pyridyl*), 2.17 (m, 2H, C*H*Me$_2$), 2.03 (m, 2H, C*H*Me$_2$), 1.26 (dvt, $J_{H–H}$ = $J_{H–P}$ = 7.0 Hz, 6H, CH*Me*$_2$), 1.18 (dvt, $J_{H–H}$ = $J_{H–P}$ = 7.2 Hz, 6H, CH*Me*$_2$), 0.86 (dvt, $J_{H–H}$ = $J_{H–P}$ = 7.1 Hz, 6H, CH*Me*$_2$), 0.58 (dvt, $J_{H–H}$ = $J_{H–P}$ = 7.2 Hz, 6H, CH*Me*$_2$), -15.69 (br, 1H, Rh–*H*). $^{31}P\{^1H\}$ NMR (202 MHz, $C_6D_6$) δ 71.6 (d, $J_{P–Rh}$ = 130.2 Hz). $^{11}B\{^1H\}$ NMR (160 MHz, $C_6D_6$) δ 2.0. $^{13}C\{^1H\}$ NMR (126 MHz, $C_6D_6$) δ 221.6 (s, free *C*O), 193.5 (br, Rh–B–*C*–N), 165.8 (br, B–*Phenyl*), 150.1 (s, *Pyridyl*), 141.0 (t, $J_{P–C}$ = 21.7 Hz, P–*Phenyl*), 132.9 (s, *Pyridyl*), 132.2 (t, $J_{P–C}$ = 9.1 Hz, *Phenyl*), 129.3 (s, *Phenyl*), 129.2 (s, *Phenyl*), 125.1 (t, $J_{P–C}$ = 3.2 Hz, *Phenyl*), 125.0 (s, *Pyridyl*), 120.4 (s, *Pyridyl*), 30.1 (t, $J_{P–C}$ = 10.1 Hz, *C*HMe$_2$), 29.2 (t, $J_{P–C}$ = 15.1 Hz, *C*HMe$_2$), 20.2 (s, CH*Me*$_2$), 19.9 (t, $J_{P–C}$ = 2.6 Hz, CH*Me*$_2$), 19.8 (t, $J_{P–C}$ = 2.9 Hz, CH*Me*$_2$), 19.6 (s, CH*Me*$_2$). (Rh–*C*O signals were not observed due to broadness). ATR-IR: $\nu_{CO}$ 1968 cm$^{-1}$ Selected spectroscopic data for **3RhC**: $^1H$ NMR (500 MHz, $C_6D_6$) δ 7.67 (d, $J_{H–H}$ = 7.7 Hz, 1H, *Pyridyl*), 7.32 (t, $J_{H–H}$ = 5.6 Hz, 2H, PBP-*Phenyl*), 6.51 (t, $J_{H–H}$ = 7.5 Hz, 1H, *Pyridyl*), 5.92 (t, $J_{H–H}$ = 6.5 Hz, 1H, *Pyridyl*), 1.98 (m, 2H, C*H*Me$_2$), 0.93 (dvt, $J_{H–H}$ = $J_{H–P}$ = 7.1 Hz, 6H, CH*Me*$_2$), 0.40 (dvt, $J_{H–H}$ = $J_{H–P}$ = 7.1 Hz, 6H, CH*Me*$_2$). -11.04 (dt, $J_{H–P}$ = 12.6 Hz, $J_{H–Rh}$ = 10.4 Hz, 1H, Rh–*H*). $^{31}P\{^1H\}$ NMR (202 MHz, $C_6D_6$) δ 78.8 (d, $J_{P–Rh}$ = 126.1 Hz). Other signals overlapped with **3RhN**.

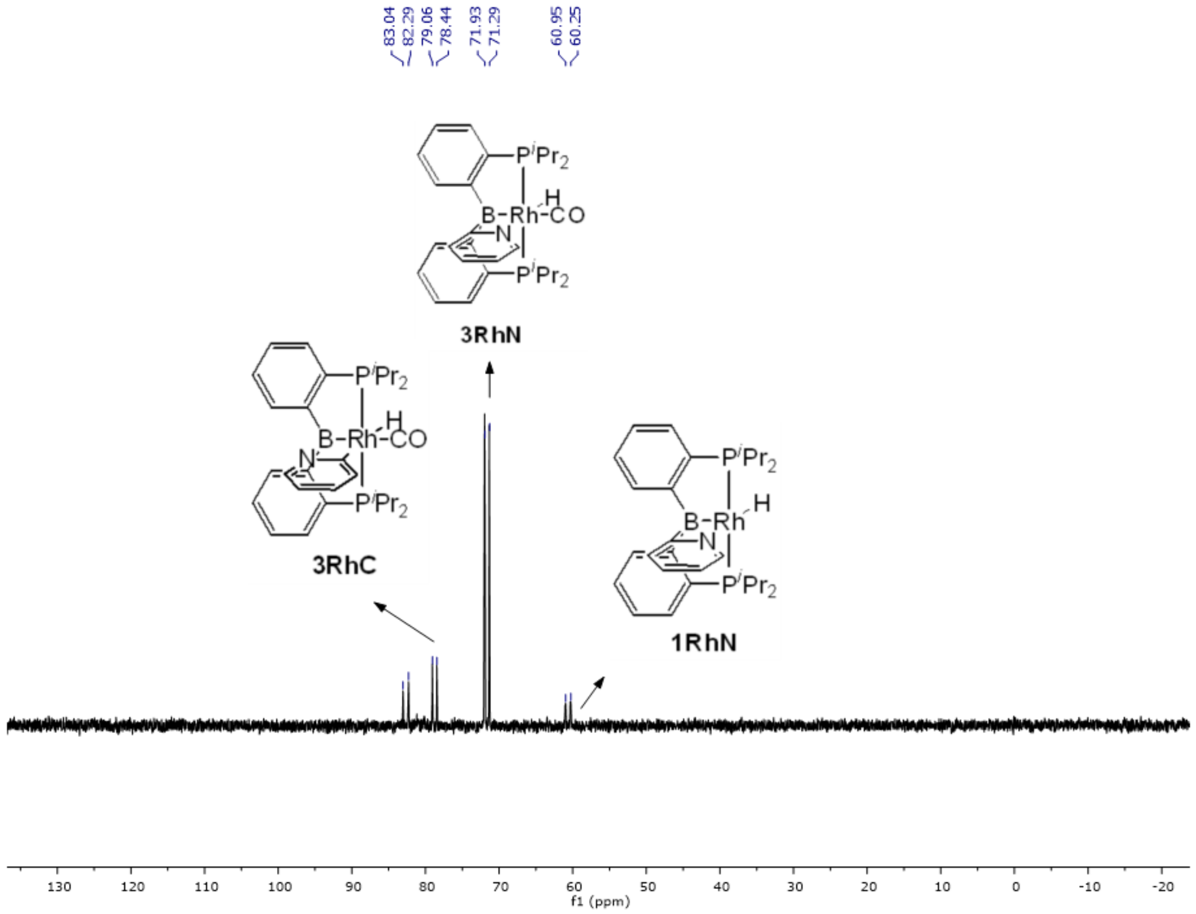


**Figure S3.** $^{31}P\{^{1}H\}$ NMR (202 MHz, $C_6D_6$) spectrum recorded at room temperature after the reaction mixture were heated at 65 °C for 24 h. The sample contains a mixture of an unidentified complex, **3RhC, 3RhN,** and **1RhN** at a ratio of 1:3:17:1.

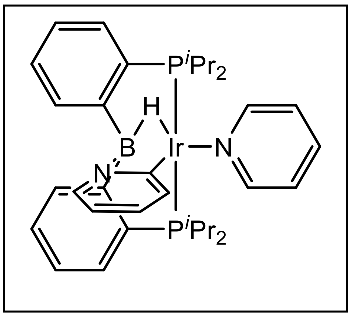

**(PBP)Ir(Py)H(Py) (7).** To **6** (0.10 mmol, 63 mg) in 2 mL toluene was add pyridine (9.0 µL, 0.10 mmol) and sodium bis(trimethylsilyl)amide (19 mg, 0.10 mmol). After being heated at 45 °C for 12 h, $^{31}P\{^1H\}$ NMR spectrum were recorded, showing a mixture of three unidentified complexes. Additional pyridine (18 µL, 0.10 mmol) were added, and the mixture was heated at 45 °C for 12 h. The reaction mixture was filtered through Celite, and volatiles were removed under vacuum. $C_6D_6$ were added and NMR spectra were recorded for characterization. $^1H$ NMR (500 MHz, $C_6D_6$) δ 8.97 (dt, $J_{H–H}$ = 1.6 Hz, 4.4 Hz, 2H, PBP-*Phenyl*), 8.53 (d, $J_{H–H}$ = 7.5 Hz, 2H, Ir–*Pyridine*), 7.85 (d, $J_{H–H}$ = 7.8 Hz, 1H, *Pyridyl*), 7.30 (m, 5H, PBP-*Phenyl & Pyridyl*), 7.18 (m, 2H, PBP-*Phenyl*), 6.94 (tt, $J_{H–H}$ = 1.8 Hz, 7.6 Hz, 1H, Ir–*Pyridine*), 6.72 (td, $J_{H–H}$ = 1.6 Hz, 7.6 Hz, 1H, *Pyridyl*), 6.58 (ddd, $J_{H–H}$ = 1.5 Hz, 4.5 Hz, 7.6 Hz, 2H, Ir–*Pyridine*), 5.93 (t, $J_{H–H}$ = 6.3 Hz, 1H, *Pyridyl*), 2.48 (m, 2H, C*H*Me$_2$), 2.33 (m, 2H, C*H*Me$_2$), 1.24 (dvt, $J_{H–H}$ = $J_{H–P}$ = 7.6 Hz, 6H, CH*Me*$_2$), 1.09 (dvt, $J_{H–H}$ = $J_{H–P}$ = 6.9 Hz, 6H, CH*Me*$_2$), 0.98 (dvt, $J_{H–H}$ = $J_{H–P}$ = 7.0 Hz, 6H, CH*Me*$_2$), 0.62 (dvt, $J_{H–H}$ = $J_{H–P}$ = 7.1 Hz, 6H, CH*Me*$_2$), -7.17 (br, 1H, Ir–*H*). $^{31}P\{^1H\}$ NMR (202 MHz, $C_6D_6$) δ 50.0 (s). $^{11}B\{^1H\}$ NMR (128 MHz, $C_6D_6$) δ -7.4. $^{13}C\{^1H\}$ NMR (126 MHz, $C_6D_6$) δ 153.4 (s), 142.5 (t, $J_{P–C}$ = 3.2 Hz), 139.3 (s), 136.4 (s), 134.8 (s), 131.7 (s), 129.6 (s), 129.2 (t, $J_{P–C}$ = 8.8 Hz), 125.7 (t, $J_{P–C}$ = 3.4 Hz), 123.7 (s), 117.9 (s), 113.4 (s), 30.6 (t, $J_{P–C}$ = 11.2 Hz), 30.2 (t, $J_{P–C}$ = 17.2 Hz), 20.2 (m, 2C), 19.9 (t, $J_{P–C}$ = 2.8 Hz), 19.6 (s).

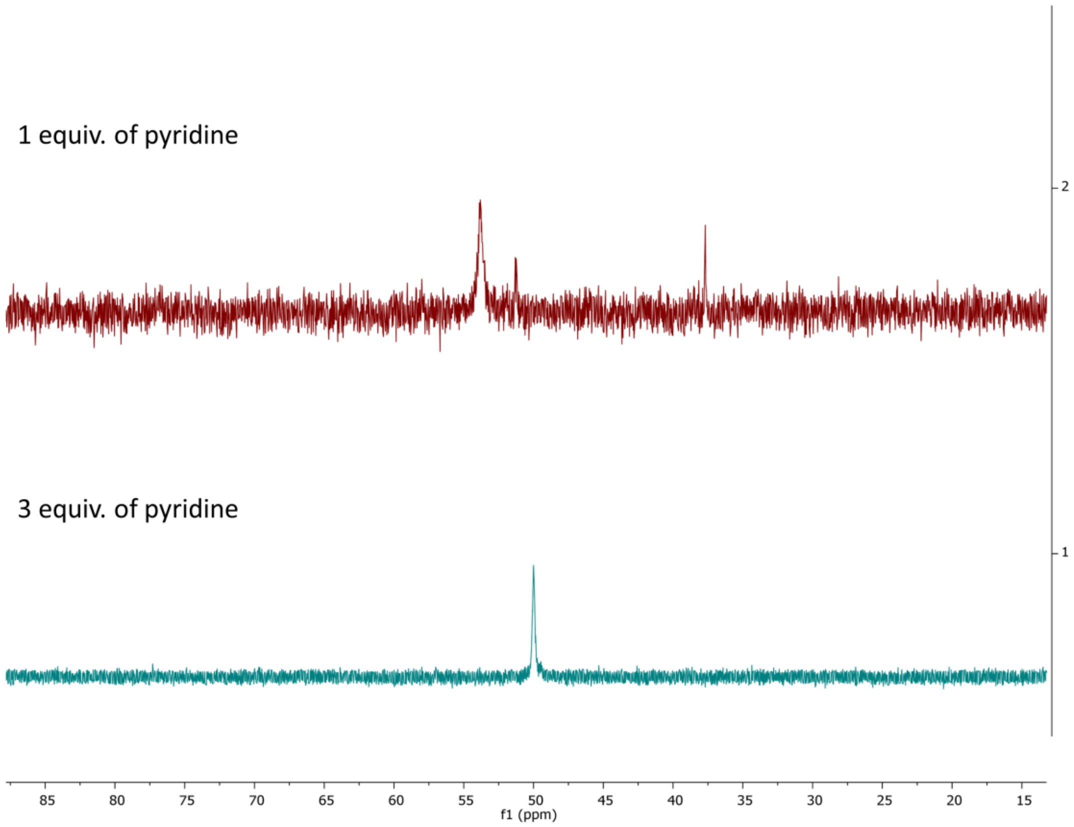


**Figure S4.** Top: $^{31}P\{^{1}H\}$ NMR recorded at room temperature on a 500 MHz Varian NMR for a mixture of **6** (0.10 mmol), pyridine (0.10 mmol) and sodium bis(trimethylsilyl)amide (0.10 mmol) in 2 mL toluene after being heated at 45 °C for 12 h. Bottom: $^{31}P\{^{1}H\}$ NMR recorded at room temperature on a 500 MHz Varian NMR for the same mixture after adding additional pyridine (0.20 mmol) and being heated at 45 °C for another 12 h.

## 3. X-ray Structural Determination Details.

**1RhN.** A light yellow, multi-faceted block of suitable size (0.46*0.22*0.16 $mm^3$) was selected from a representative sample of crystals of the same habit using an optical microscope and mounted onto a nylon loop. Low temperature (110 K) X-ray data were obtained on a Bruker APEXII CCD based diffractometer (Mo sealed X-ray tube, $K_\alpha$ = 0.71073 Å). All diffractometer manipulations, including data collection, integration and scaling were carried out using the Bruker APEXII software.[2] An absorption correction was applied using SADABS.[3] The space group was determined on the basis of systematic absences and intensity statistics and the structure was solved by direct methods and refined by full-matrix least squares on $F^2$. The structure was solved in the monoclinic P $2_1$/c space group using XS[4] (incorporated in Olex). All non-hydrogen atoms were refined with anisotropic thermal parameters. All hydrogen atoms were placed in idealized positions and refined using riding model with the exception of the hydrogen bound to rhodium which was located from the difference map. The structure was refined (weighted least squares refinement on $F^2$) and the final least-squares refinement converged. No additional symmetry was found using ADDSYM incorporated in PLATON program.[5] CCDC 2014200 contain the supplementary crystallographic data.

**1RhNq.** A Leica MZ $7_5$ microscope was used to identify a suitable yellow block with very well-defined faces with dimensions (max, intermediate, and min) 0.368 x 0.36 x 0.164 $mm^3$ from a representative sample of crystals of the same habit. The crystal mounted on a nylon loop was then placed in a cold nitrogen stream (Oxford) maintained at 110 K. A BRUKER APEX 2 X-ray (three-circle) diffractometer was employed for crystal screening, unit cell determination, and data collection. The goniometer was controlled using the APEX2 software suite, v2008-6.0.[6] The sample was optically centered with the aid of a video camera such that no translations were

observed as the crystal was rotated through all positions. The detector was set at 6.0 cm from the crystal sample (APEX2, 512x512 pixel). The X-ray radiation employed was generated from a Mo sealed X-ray tube ($K_\alpha$ = 0.70173Å with a potential of 40 kV and a current of 40 mA). Sixty data frames were taken at widths of 1.0°. These reflections were used in the auto-indexing procedure to determine the unit cell. A suitable cell was found and refined by nonlinear least squares and Bravais lattice procedures. The unit cell was verified by examination of the *h k l* overlays on several frames of data. No super-cell or erroneous reflections were observed. After careful examination of the unit cell, an extended data collection procedure (4 sets) was initiated using omega scans. Integrated intensity information for each reflection was obtained by reduction of the data frames with the program APEX2.[6] The integration method employed a three-dimensional profiling algorithm and all data were corrected for Lorentz and polarization factors, as well as for crystal decay effects. Finally, the data was merged and scaled to produce a suitable data set. The absorption correction program SADABS[3] was employed to correct the data for absorption effects. Systematic reflection conditions and statistical tests of the data suggested the space group *P*-1. A solution was obtained readily using XT/XS in APEX2.[4,6] Hydrogen atoms were placed in idealized positions and were set riding on the respective parent atoms. All non-hydrogen atoms were refined with anisotropic thermal parameters. Absence of additional symmetry and voids were confirmed using PLATON (ADDSYM).[5] The structure was refined (weighted least squares refinement on $F^2$) to convergence.[4,7] CCDC 2014201 contain the supplementary crystallographic data.

**1IrCq.** A Leica MZ $7_5$ microscope was used to identify an orange plate with very well-defined faces with dimensions (max, intermediate, and min) 0.528 x 0.213 x 0.046 $mm^3$ from a representative sample of crystals of the same habit. The crystal mounted on a nylon loop was then placed in a cold nitrogen stream (Oxford) maintained at 110 K. A BRUKER APEX 2 Duo X-ray

(three-circle) diffractometer was employed for crystal screening, unit cell determination, and data collection. The goniometer was controlled using the APEX3 software suite, v2017.3-0.[8] The sample was optically centered with the aid of a video camera such that no translations were observed as the crystal was rotated through all positions. The detector (Bruker - PHOTON) was set at 6.0 cm from the crystal sample. The X-ray radiation employed was generated from a Mo sealed X-ray tube ($K_\alpha$ = 0.71073Å with a potential of 40 kV and a current of 40 mA). 45 data frames were taken at widths of 1.0°. These reflections were used in the auto-indexing procedure to determine the unit cell. A suitable cell was found and refined by nonlinear least squares and Bravais lattice procedures. The unit cell was verified by examination of the *h k l* overlays on several frames of data. No super-cell or erroneous reflections were observed. After careful examination of the unit cell, an extended data collection procedure (16 sets) was initiated using omega scans. Integrated intensity information for each reflection was obtained by reduction of the data frames with the program APEX3.[8] The integration method employed a three-dimensional profiling algorithm and all data were corrected for Lorentz and polarization factors, as well as for crystal decay effects. Finally, the data was merged and scaled to produce a suitable data set. The absorption correction program SADABS[3] was employed to correct the data for absorption effects. Systematic reflection conditions and statistical tests of the data suggested the space group *P*-1. A solution was obtained readily (Z=4; Z'=2) using XT/XS in APEX2.[4,8] A molecule of toluene was found solvated (1/2 a molecule per Ir-complex). Hydrogen atoms were placed in idealized positions and were set riding on the respective parent atoms. All non-hydrogen atoms were refined with anisotropic thermal parameters. Elongated ellipsoids and nearby residual electron density peaks for C64 – C66 indicated disorder which was modeled between two positions with an occupancy ratio of 0.77:0.23. Appropriate restraints and constraints were added to keep the bond

distances, angles, and thermal ellipsoids meaningful. Absence of additional symmetry and voids were confirmed using PLATON (ADDSYM). The structure was refined (weighted least squares refinement on $F^2$) to convergence.[4,7] CCDC 2014205 contain the supplementary crystallographic data.

**2RhC.** A Leica MZ $7_5$ microscope was used to identify a suitable yellow block with very well-defined faces with dimensions (max, intermediate, and min) 0.222 x 0.19 x 0.182 $mm^3$ from a representative sample of crystals of the same habit. The crystal mounted on a nylon loop was then placed in a cold nitrogen stream (Oxford) maintained at 110 K. A BRUKER APEX 2 X-ray (three-circle) diffractometer was employed for crystal screening, unit cell determination, and data collection. The goniometer was controlled using the APEX2 software suite, v2008-6.0.[6] The sample was optically centered with the aid of a video camera such that no translations were observed as the crystal was rotated through all positions. The detector was set at 6.0 cm from the crystal sample (APEX2, 512x512 pixel). The X-ray radiation employed was generated from a Mo sealed X-ray tube ($K_\alpha$ = 0.70173Å with a potential of 40 kV and a current of 40 mA). Sixty data frames were taken at widths of 1.0°. These reflections were used in the auto-indexing procedure to determine the unit cell. A suitable cell was found and refined by nonlinear least squares and Bravais lattice procedures. The unit cell was verified by examination of the *h k l* overlays on several frames of data. No super-cell or erroneous reflections were observed. After careful examination of the unit cell, an extended data collection procedure (4 sets) was initiated using omega scans. Integrated intensity information for each reflection was obtained by reduction of the data frames with the program APEX2.[6] The integration method employed a three-dimensional profiling algorithm and all data were corrected for Lorentz and polarization factors, as well as for crystal decay effects. Finally, the data was merged and scaled to produce a suitable data set. The

absorption correction program SADABS[3] was employed to correct the data for absorption effects. Systematic reflection conditions and statistical tests of the data suggested the space group $P4_3$. A solution was obtained readily using XT/XS in APEX2.[4,6] Hydrogen atoms were placed in idealized positions and were set riding on the respective parent atoms. All non-hydrogen atoms were refined with anisotropic thermal parameters. Elongated thermal ellipsoids on C27 and C28 suggested disorder, which was modeled successfully between two positions with an occupancy ratio of 0.42 to 0.58. Appropriate restraints were added to keep the bond distances, angles and thermal ellipsoids meaningful. Absence of additional symmetry and voids were confirmed using PLATON (ADDSYM).[5] The structure was refined (weighted least squares refinement on $F^2$) to convergence.[4,7] CCDC 2014203 contain the supplementary crystallographic data.

**3RhN.** A Leica MZ $7_5$ microscope was used to identify a suitable colorless block with very well-defined faces with dimensions (max, intermediate, and min) 0.774 x 0.706 x 0.429 mm$^3$ from a representative sample of crystals of the same habit. The crystal mounted on a nylon loop was then placed in a cold nitrogen stream (Oxford) maintained at 110 K. A BRUKER APEX 2 Duo X-ray (three-circle) diffractometer was employed for crystal screening, unit cell determination, and data collection. The goniometer was controlled using the APEX3 software suite, v2017.3-0.[8] The sample was optically centered with the aid of a video camera such that no translations were observed as the crystal was rotated through all positions. The detector was set at 6.0 cm from the crystal sample (APEX2, 512x512 pixel). The X-ray radiation employed was generated from a Mo sealed X-ray tube ($K_\alpha$ = 0.70173Å with a potential of 40 kV and a current of 40 mA). 45 data frames were taken at widths of 1.0°. These reflections were used in the auto-indexing procedure to determine the unit cell. A suitable cell was found and refined by nonlinear least squares and Bravais lattice procedures. The unit cell was verified by examination of the *h k l* overlays on several

frames of data. No super-cell or erroneous reflections were observed. After careful examination of the unit cell, an extended data collection procedure (24 sets) was initiated using omega and phi scans. Integrated intensity information for each reflection was obtained by reduction of the data frames with the program APEX3.[8] The integration method employed a three-dimensional profiling algorithm and all data were corrected for Lorentz and polarization factors, as well as for crystal decay effects. Finally, the data was merged and scaled to produce a suitable data set. The absorption correction program SADABS[3] was employed to correct the data for absorption effects. Systematic reflection conditions and statistical tests of the data suggested the space group $P2_1/n$. A solution was obtained readily using XT/XS in APEX2.[4,8] Hydrogen atoms were placed in idealized positions (the one connected to Rh was refined independently) and were set riding on the respective parent atoms. All non-hydrogen atoms were refined with anisotropic thermal parameters. Absence of additional symmetry and voids were confirmed using PLATON (ADDSYM).[5] The structure was refined (weighted least squares refinement on $F^2$) to convergence.[4,7] CCDC 2014204 contain the supplementary crystallographic data.

ORTEP-3 for Windows and POV-Ray were employed for the final data presentation and structure plots.[9,10]

## 4. DFT Calculations.

The Gaussian suite of programs[11] was used for the ab initio electronic structure calculations. All structures were fully optimized by the B97D3[12] functional in the gas phase, and harmonic vibrational frequency calculations were performed to ensure that either a minimum was obtained. The Los Alamos basis set and the associated effective core potential (ECP) was used for Ru and Ir atoms, and an all-electron 6-31G(d) basis set was used for all the other atoms. Unless otherwise stated, the energies reported in this paper are Gibbs free energies under 298.15 K and 1 atm with solvent effect corrections.

The more recent natural orbitals for chemical valence (NOCV) analyses[13] implemented as part of the ORCA program[14] were applied to study the chemical bonds in terms of the electron density rearrangement taking place upon bond formation at the B97D3/def2-TZVP level using the Extended Transition State (ETS) method of Ziegler. The figures of NOCV deformation densities are generated by Multiwfn code.[15]

**Table S1.** Absolute free energies and enthalpies in the gas phase at 298K and free energies and enthalpies for the M–C/M–N isomerizations (negative values favor the M-N isomer) calculated at the B97D3/LANL2DZ/6-31G(d) level in gas phase.

| | Absolute values of Gibbs free energy (G) at 298 K (a. u.) | Absolute values of enthalpy (H) (a. u.) | $\Delta G_{isomerization}$ (kcal/mol) | $\Delta H_{isomerization}$ = (kcal/mol) |
|---|---|---|---|---|
| **1RhC** | -2000.592744 | -2000.486785 | | |
| **1RhN** | -2000.599909 | -2000.492665 | -4.5 | -3.7 |
| **1IrC** | -1995.792319 | -1995.686313 | | |
| **1IrN** | -1995.789668 | -1995.681918 | 1.7 | 2.8 |
| **2RhC** | -4572.779866 | -4572.670542 | | |
| **2RhN** | -4572.762453 | -4572.652586 | 10.9 | 11.3 |
| **2IrC** | -4567.973024 | -4567.863829 | | |
| **2IrN** | -4567.946079 | -4567.836552 | 16.9 | 17.1 |
| **3RhC** | -2113.872887 | -2113.761183 | | |
| **3RhN** | -2113.875726 | -2113.763486 | -1.8 | -1.4 |
| **3IrC** | -2109.088223 | -2108.977483 | | |
| **3IrN** | -2109.081008 | -2108.969473 | 4.5 | 5.0 |
| **4RhC** | -2113.847909 | -2113.738511 | | |
| **4RhN** | -2113.84592 | -2113.73573 | 1.2 | 1.7 |
| **4IrC** | -2109.06682 | -2108.958266 | | |
| **4IrN** | -2109.057584 | -2108.948267 | 5.8 | 6.3 |

**Table S2.** Wiberg bond indices calculated at the B97D3/LANL2DZ/6-31G(d) level of theory for Type 1-4 Rh and Ir complexes.

| | Wiberg Bond Index | | | |
|---|---|---|---|---|
| | M-B | B-C/N | C-N | M-N/C |
| **1RhC** | 0.58 | 0.59 | 1.23 | 0.59 |
| **1RhN** | 0.56 | 0.81 | 1.31 | 0.28 |
| **1IrC** | 0.66 | 0.59 | 1.22 | 0.67 |
| **1IrN** | 0.65 | 0.81 | 1.30 | 0.32 |
| **2RhC** | 0.52 | 0.58 | 1.24 | 0.75 |
| **2RhN** | 0.52 | 0.80 | 1.29 | 0.43 |
| **2IrC** | 0.60 | 0.57 | 1.23 | 0.83 |
| **2IrN** | 0.61 | 0.79 | 1.27 | 0.50 |
| **3RhC** | 0.51 | 0.59 | 1.26 | 0.65 |
| **3RhN** | 0.48 | 0.82 | 1.31 | 0.38 |
| **3IrC** | 0.59 | 0.59 | 1.25 | 0.73 |
| **3IrN** | 0.56 | 0.81 | 1.29 | 0.46 |
| **4RhC** | 0.49 | 0.58 | 1.27 | 0.68 |
| **4RhN** | 0.41 | 0.81 | 1.31 | 0.45 |
| **4IrC** | 0.58 | 0.57 | 1.27 | 0.72 |
| **4IrN** | 0.54 | 0.80 | 1.29 | 0.50 |

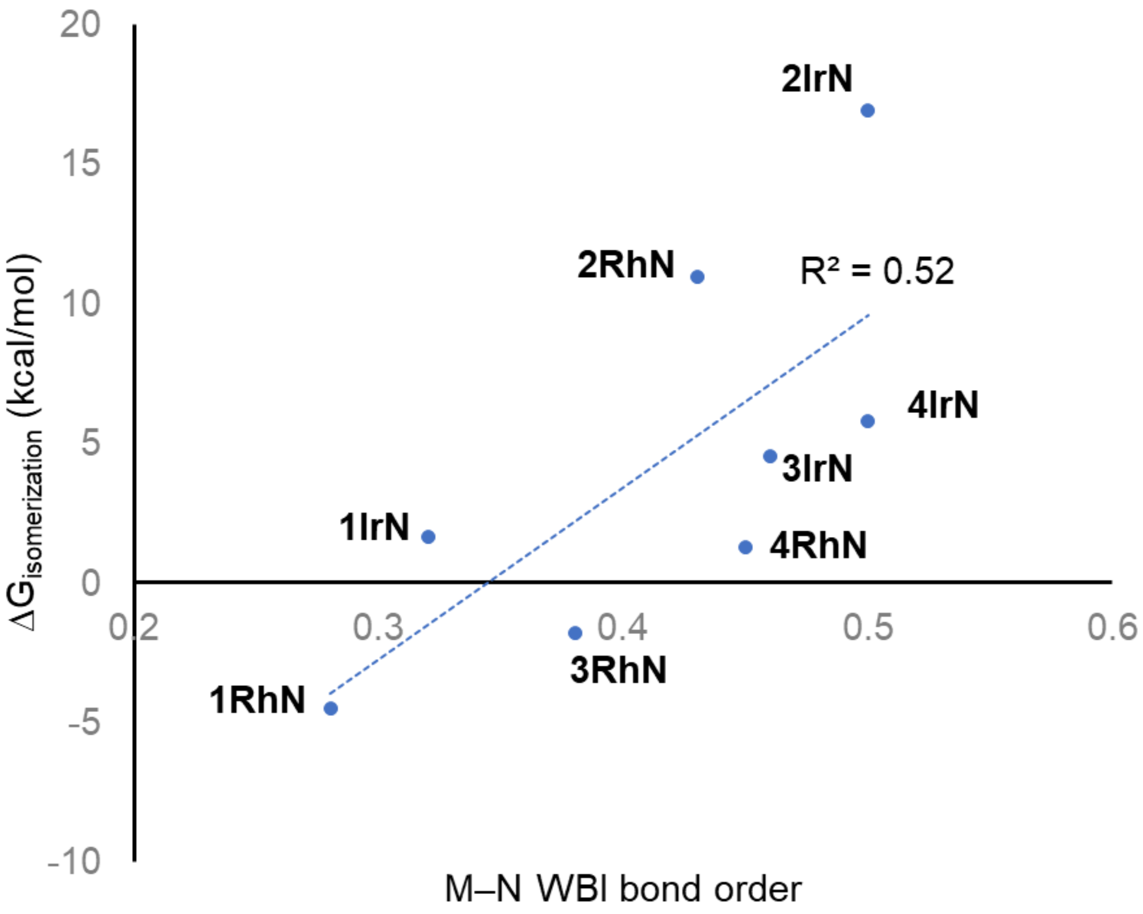


**Figure S5.** Correlation between $\Delta G_{isomerization}$ and M–N WBI bond order.

**Table S3.** ETS-NOCV analysis of the interaction between 2-pyridyl anion and the formally positive charged rest of the molecule. The two largest decomposed NOCV contributions (Δρ1 and Δρ2) constructed with the density isosurface contour value of 0.002 are shown. Blue and red surfaces identify regions of electron density accumulation and depletion, respectively. It is clear the electrons "flow" from CN anion to metal cation.

| | Sum of decomposition energy by ETS-NOCV (kcal/mol) | The Two Major Components | |
|---|---|---|---|
| **1RhC** | -243.2 | 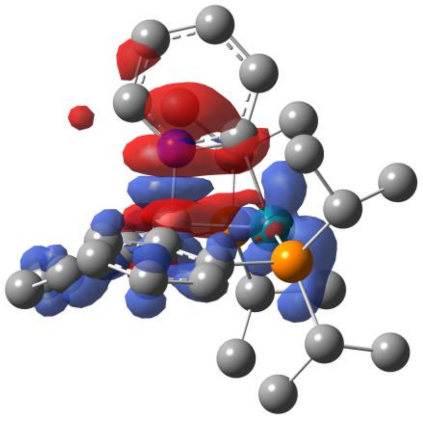 $\Delta\rho$ 1 = -105 kcal/mol | 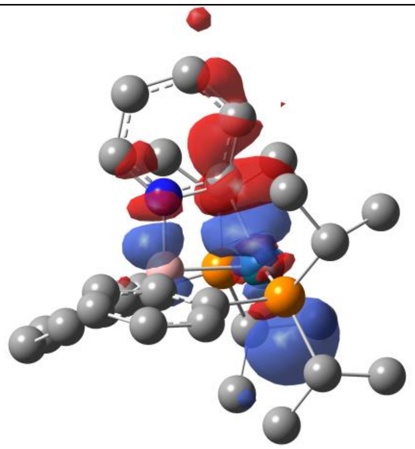 $\Delta\rho$ 2 = -76 kcal/mol |
| **1RhN** | -232.0 | 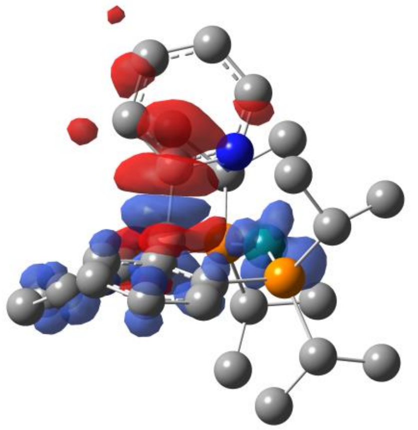 $\Delta\rho$ 1 = -153 kcal/mol | 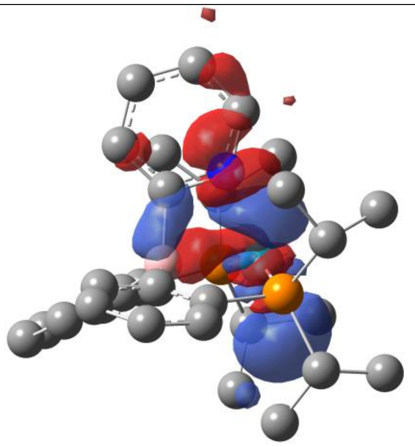 $\Delta\rho$ 2 = -30 kcal/mol |

| | | | |
|---|---|---|---|
| **1IrC** | -343.7 | 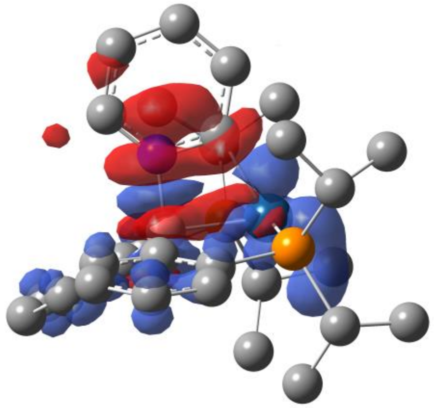<br>$\Delta\rho$ 1 = -155 kcal/mol | 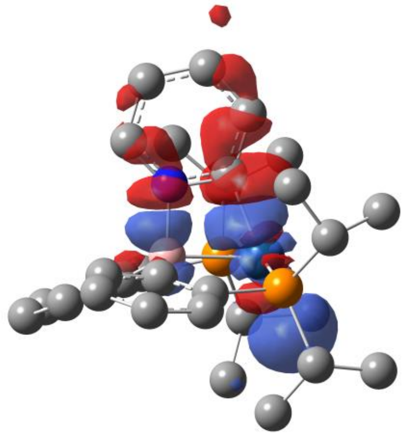<br>$\Delta\rho$ 2 = -117 kcal/mol |
| **1IrN** | -255.3 | 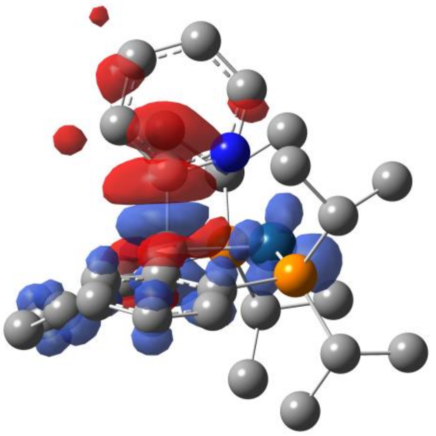<br>$\Delta\rho$ 1 = -150 kcal/mol | 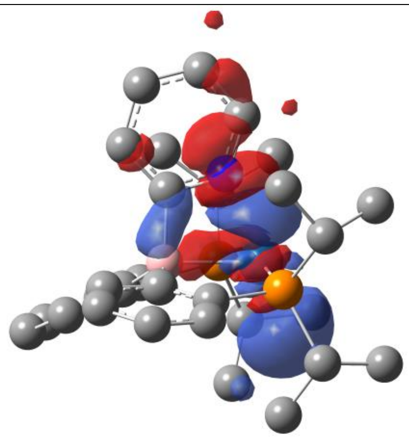<br>$\Delta\rho$ 2 = -51 kcal/mol |
| **2RhC** | -310.9 | 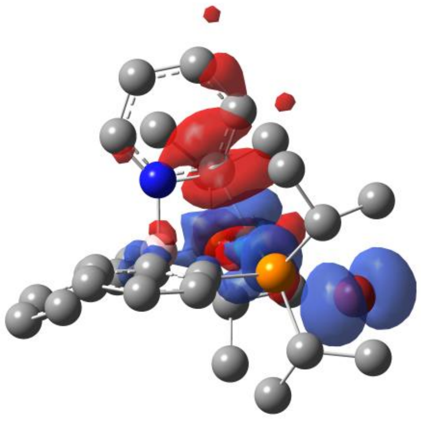<br>$\Delta\rho$ 1 = -131 kcal/mol | 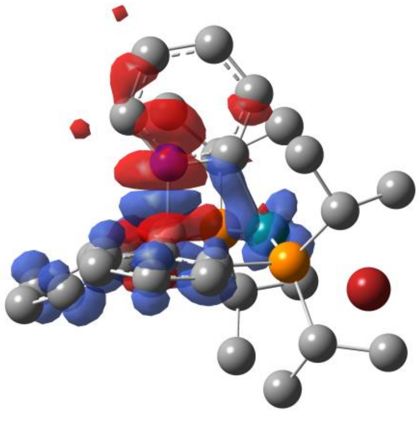<br>$\Delta\rho$ 2 = -106 kcal/mol |

| | | | |
|---|---|---|---|
| **2RhN** | -271.0 | 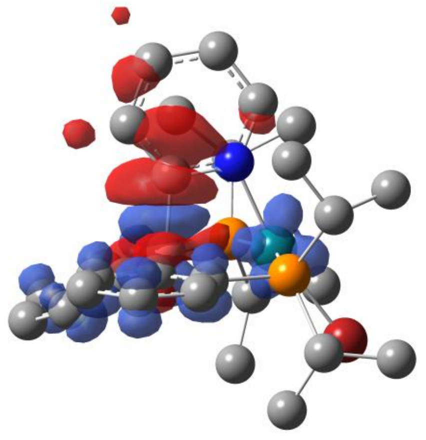 Δ ρ 1 = -157 kcal/mol | 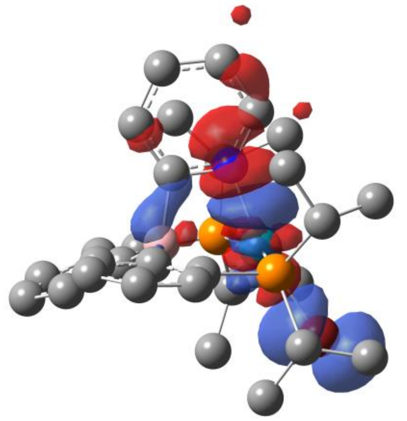 Δ ρ 2 = -56 kcal/mol |
| **2IrC** | -467.0 | 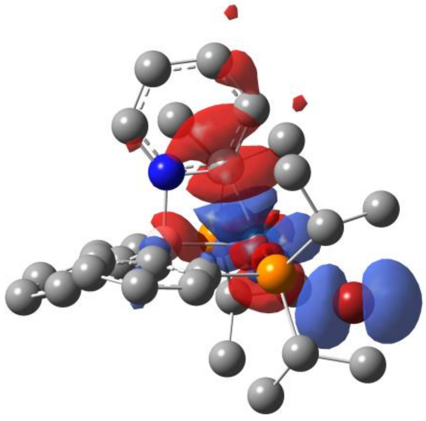 Δ ρ 1 = -260 kcal/mol | 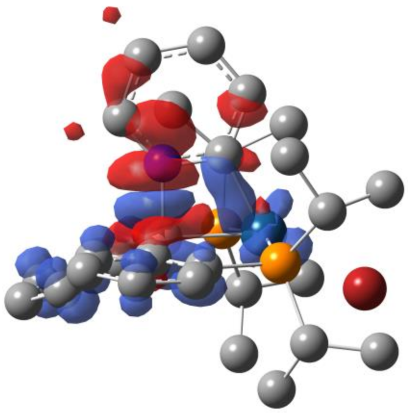 Δ ρ 2 = -111 kcal/mol |
| **2IrN** | -313.1 | 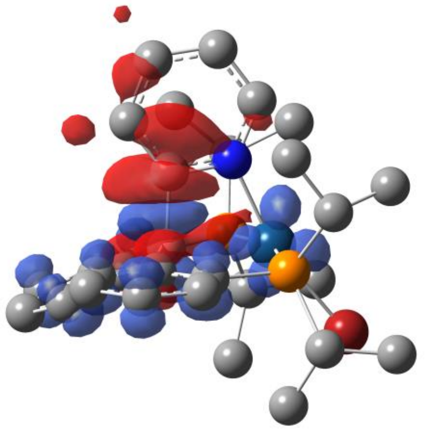 Δ ρ 1 = -153 kcal/mol | 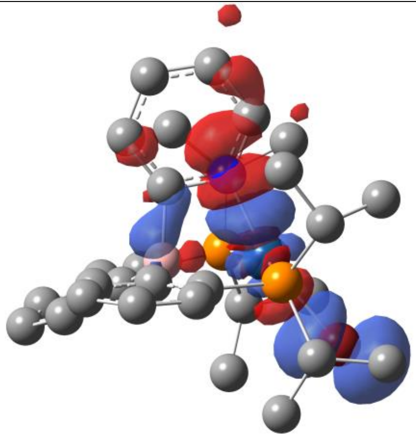 Δ ρ 2 = -96 kcal/mol |

| | | | |
|---|---|---|---|
| **3RhC** | -241.0 | 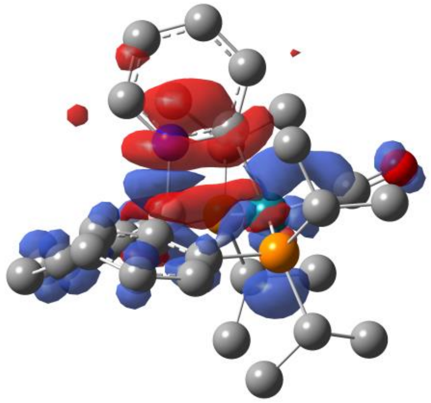 Δ $\rho$ 1 = -101 kcal/mol | 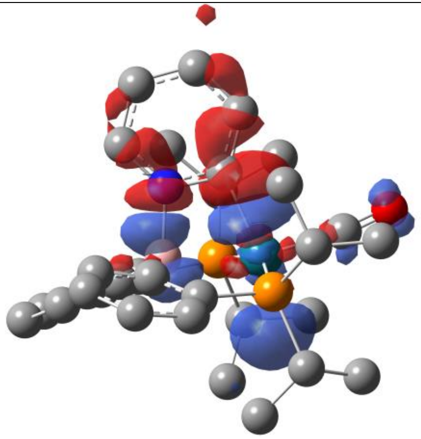 Δ $\rho$ 2 = -83 kcal/mol |
| **3RhN** | -236.3 | 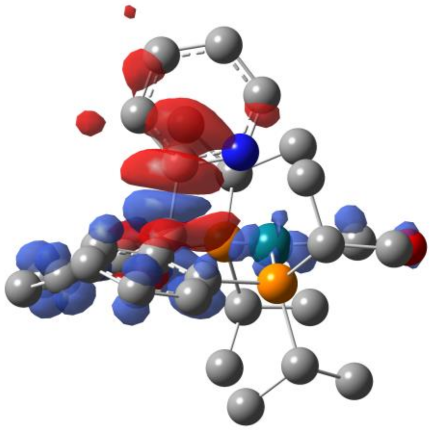 Δ $\rho$ 1 = -153 kcal/mol | 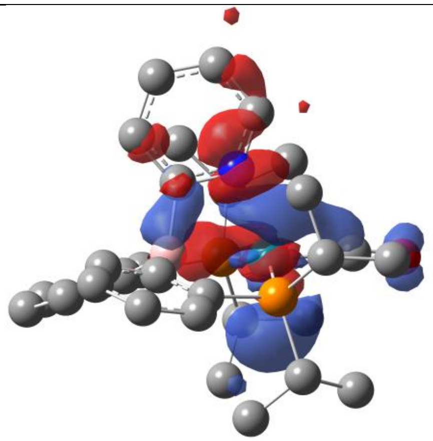 Δ $\rho$ 2 = -36 kcal/mol |
| **3IrC** | -329.8 | 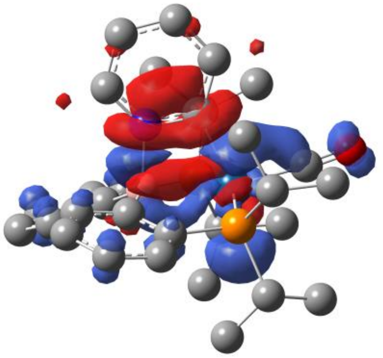 Δ $\rho$ 1 = -135 kcal/mol | 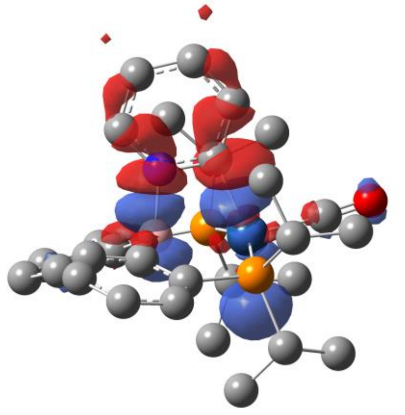 Δ $\rho$ 2 = -129 kcal/mol |

| | | | |
|---|---|---|---|
| **3IrN** | -259.6 | 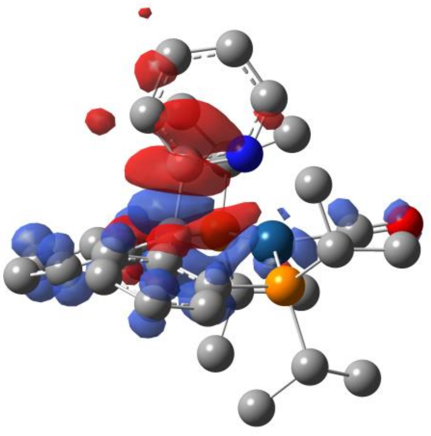 Δ $\rho$ 1 = -148 kcal/mol | 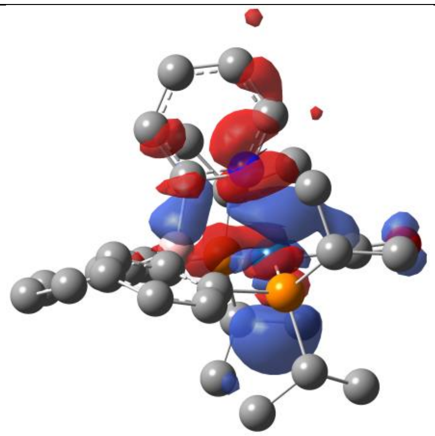 Δ $\rho$ 2 = -58 kcal/mol |
| **4RhC** | -282.6 | 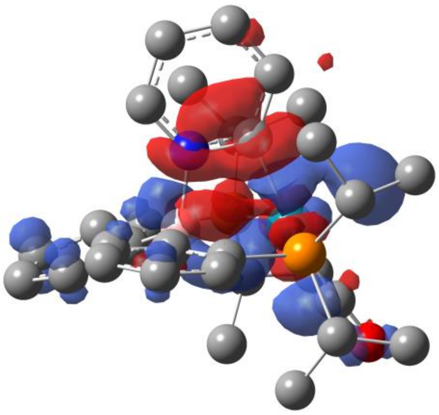 Δ $\rho$ 1 = -125 kcal/mol | 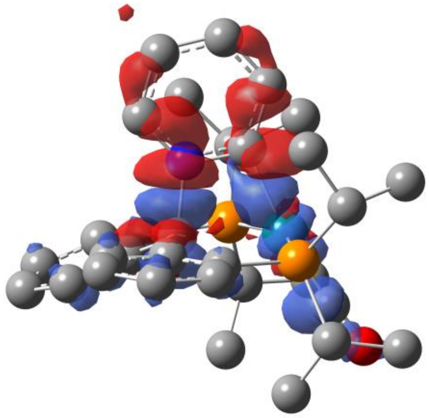 Δ $\rho$ 2 = -97 kcal/mol |
| **4RhN** | -274.6 | 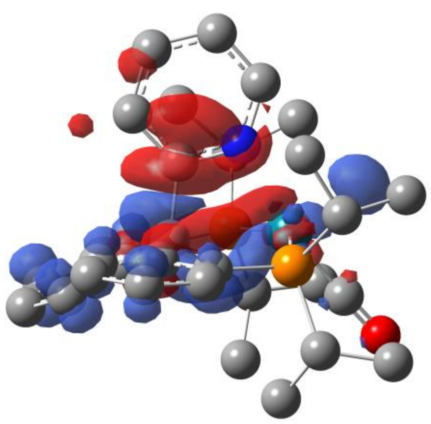 Δ $\rho$ 1 = -159 kcal/mol | 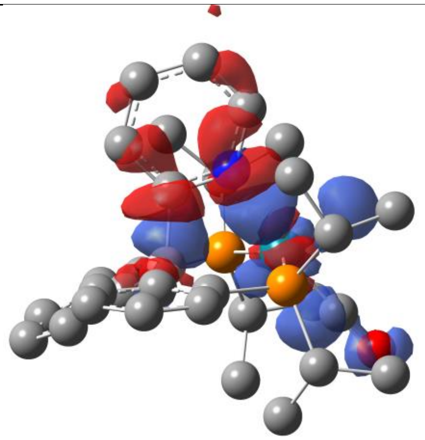 Δ $\rho$ 2 = -65 kcal/mol |

| | | | |
|---|---|---|---|
| **4IrC** | -366.9 | 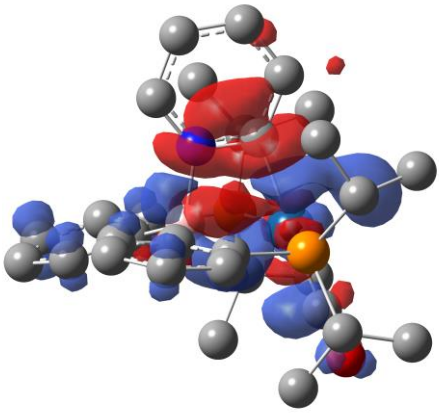<br>Δ $\rho$ 1 = -162 kcal/mol | 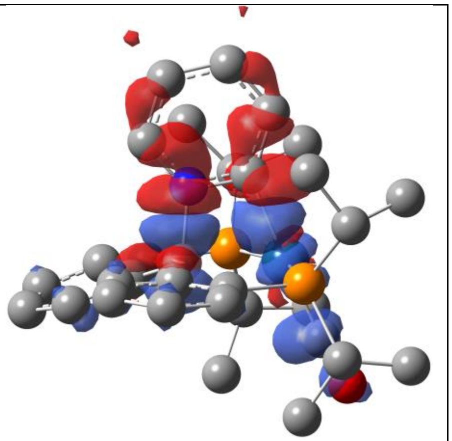<br>Δ $\rho$ 2 = -131 kcal/mol |
| **4IrN** | -297.1 | 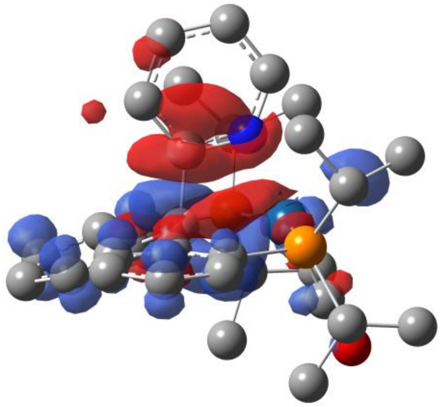<br>Δ $\rho$ 1 = -154 kcal/mol | 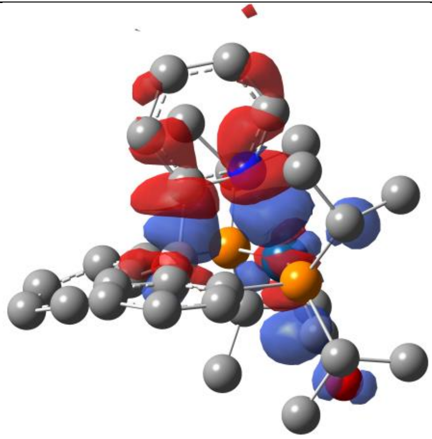<br>Δ $\rho$ 2 = -82 kcal/mol |

## 5. NMR Spectra.

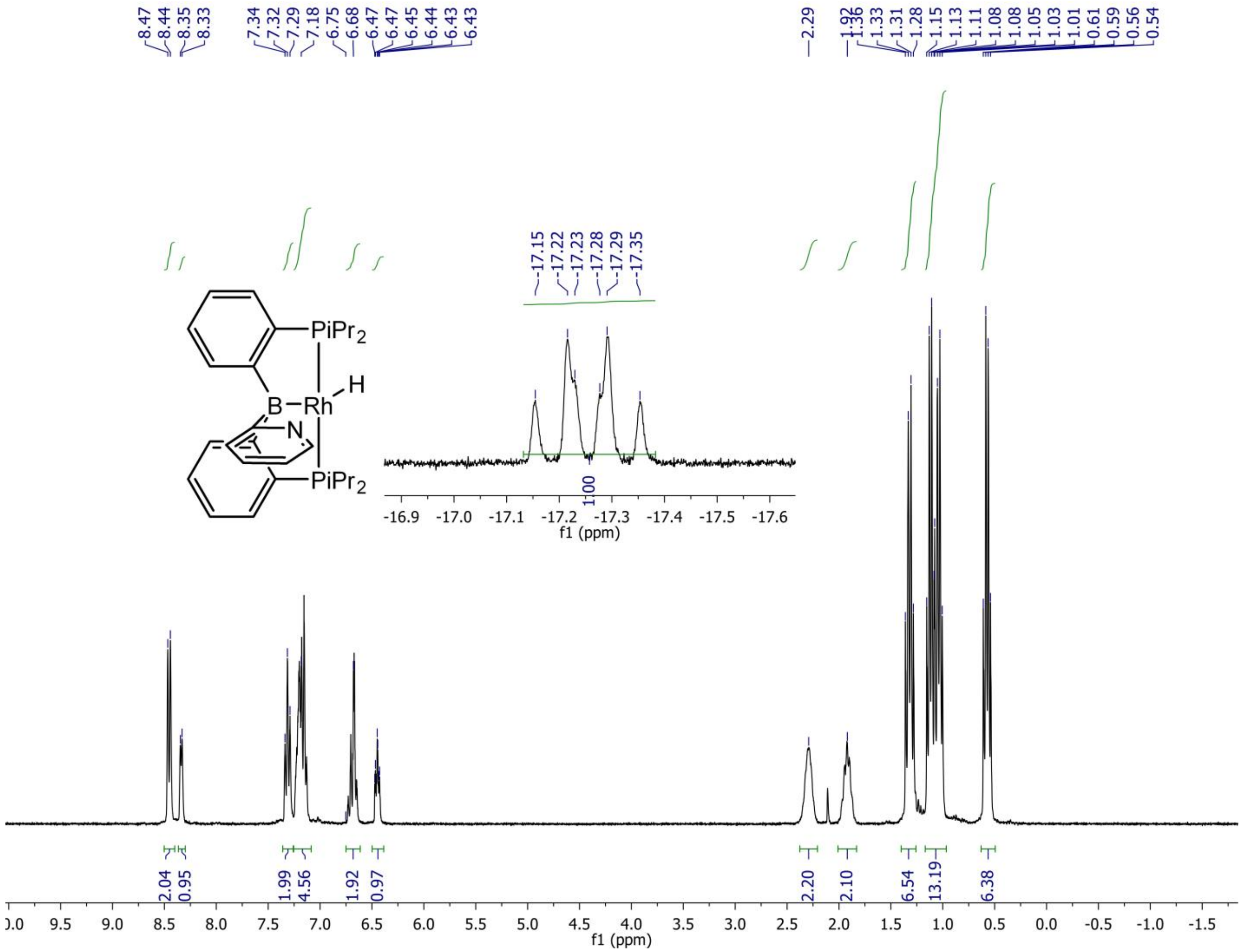

**Figure S6.** $^{1}$H NMR spectrum of **1RhN** in $C_6D_6$ measured on a 300 MHz Mercury NMR

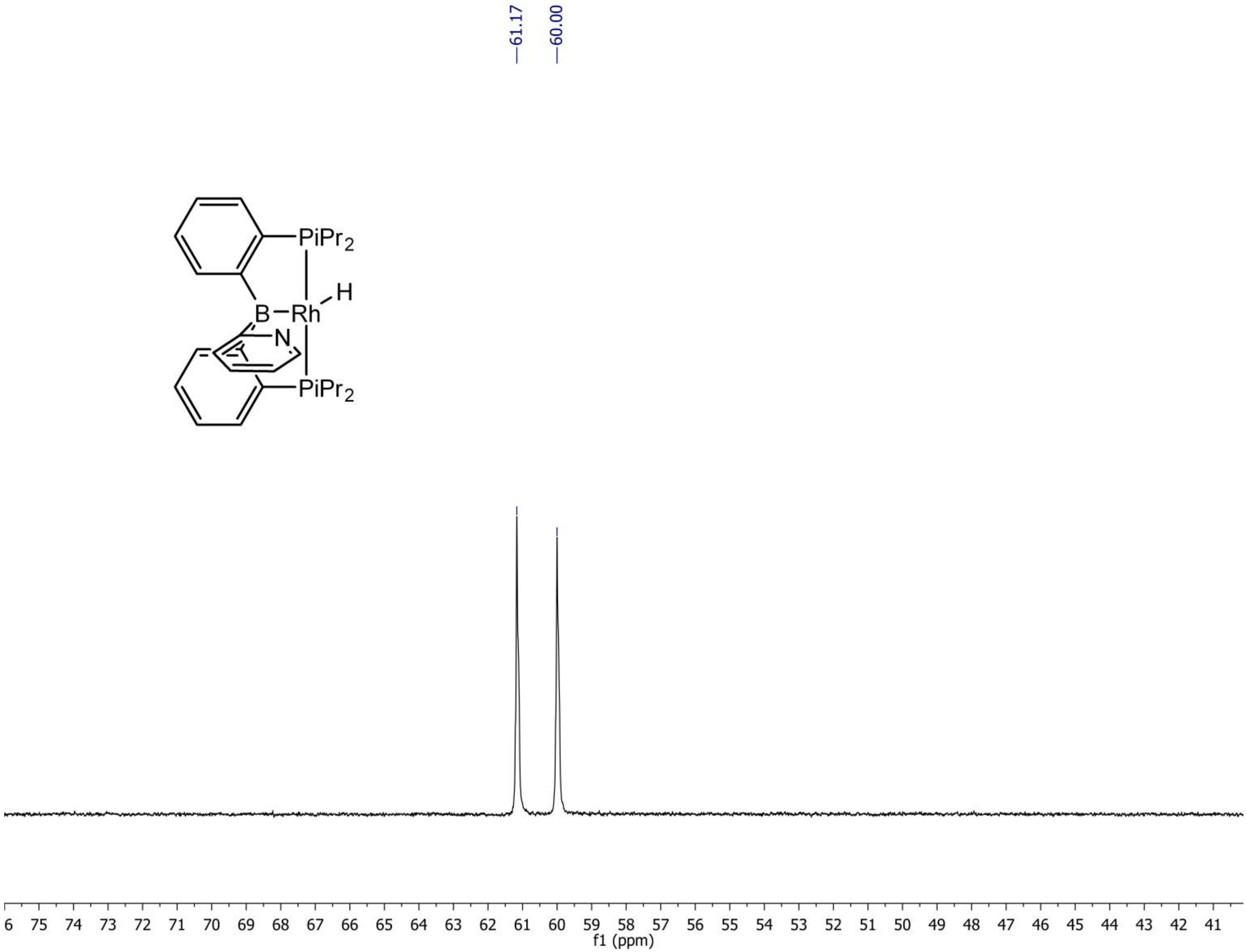


**Figure S7.** $^{31}P\{^{1}H\}$ NMR spectrum of **1RhN** in $C_6D_6$ measured on a 300 MHz Mercury NMR

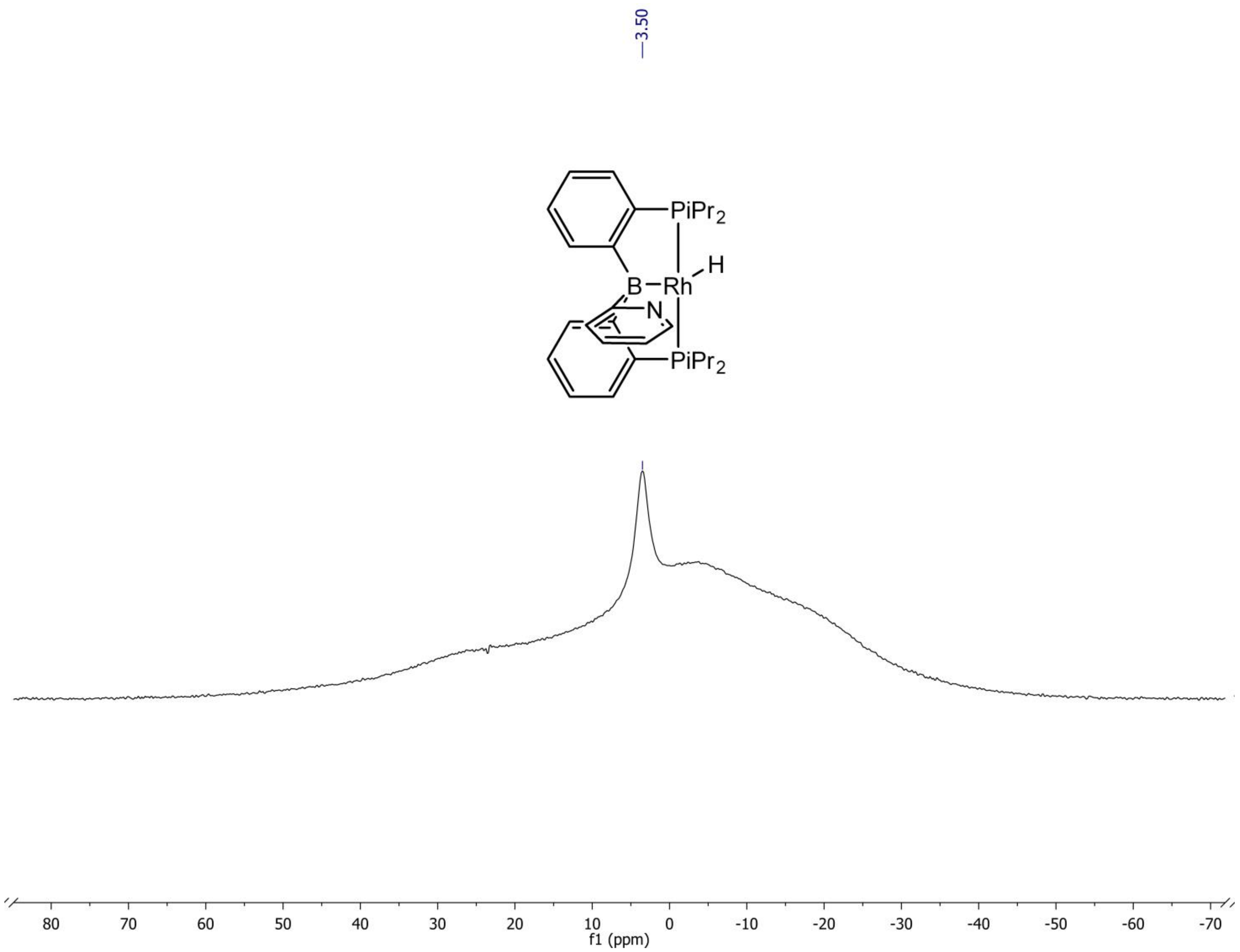


**Figure S8.** $^{11}B\{^1H\}$ NMR spectrum of **1RhN** in $C_6D_6$ measured on a 400 MHz Varian NMR

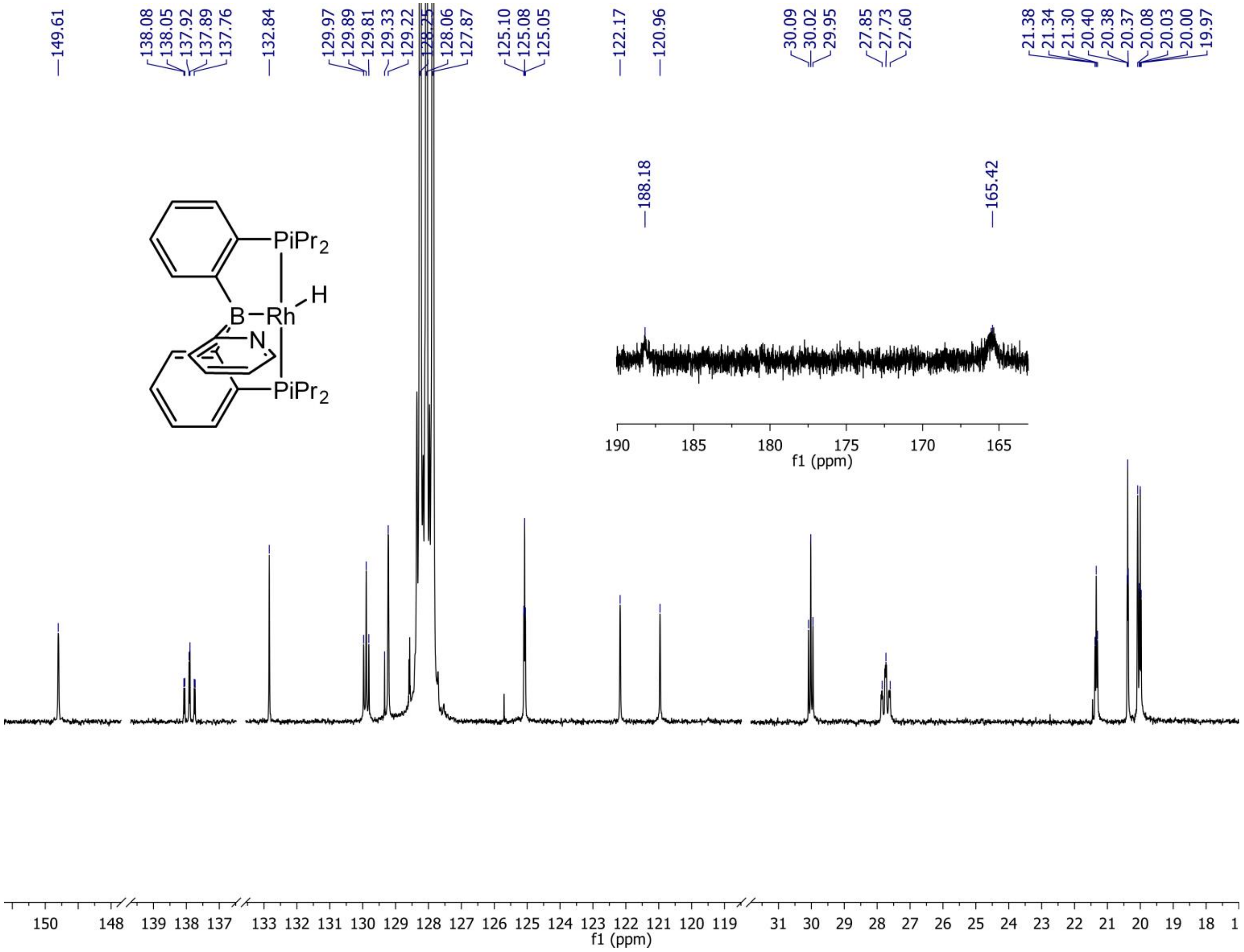


**Figure S9.** $^{13}C\{^{1}H\}$ NMR of **1RhN** in $C_6D_6$ measured on a 500 MHz Varian NMR.

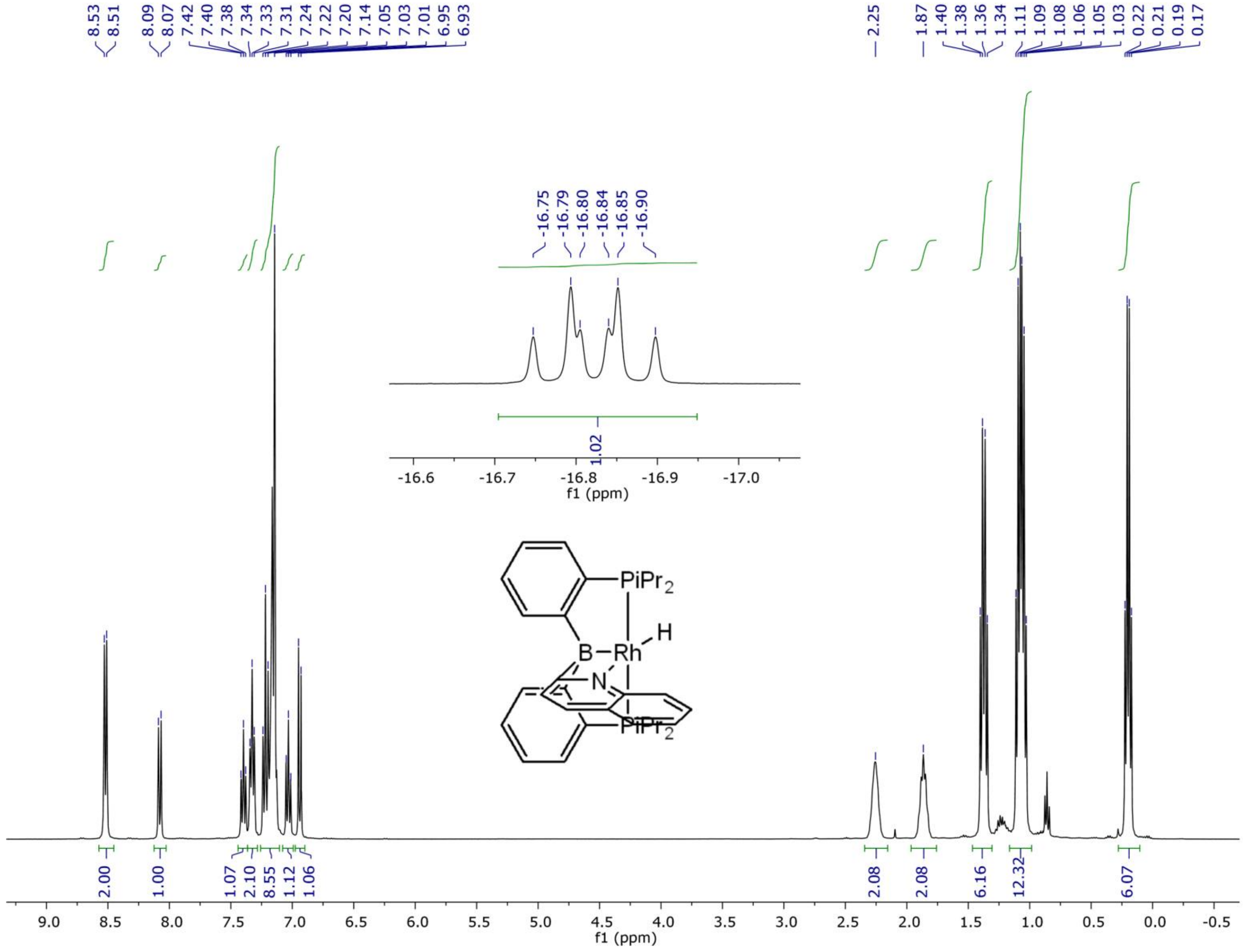

**Figure S10.** $^{1}$H NMR spectrum of **1RhNq** in $C_6D_6$ measured on a 400 MHz Bruker NMR

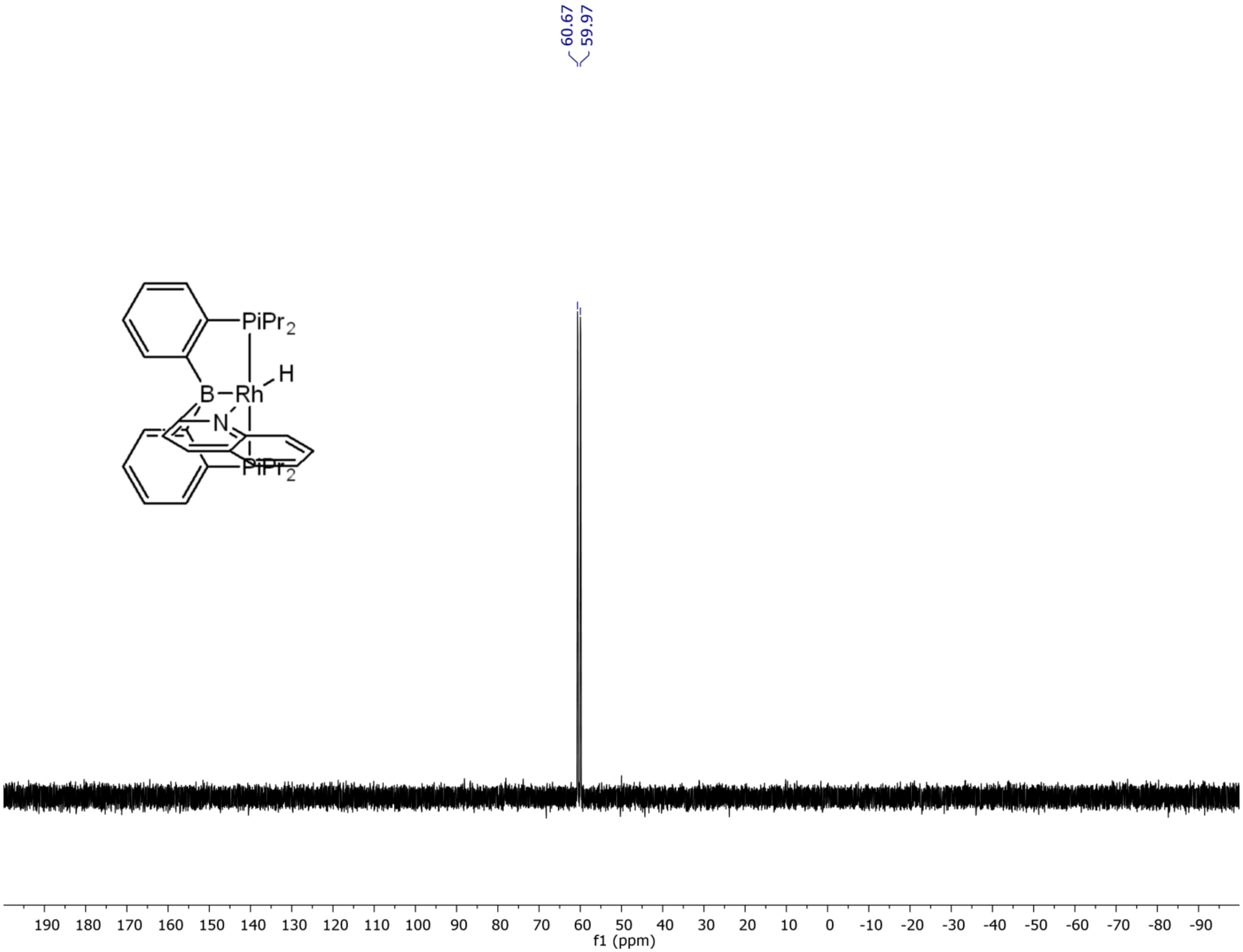


**Figure S11.** $^{31}P\{^{1}H\}$ NMR spectrum of **1RhNq** in $C_6D_6$ measured on a 300 MHz Mercury NMR

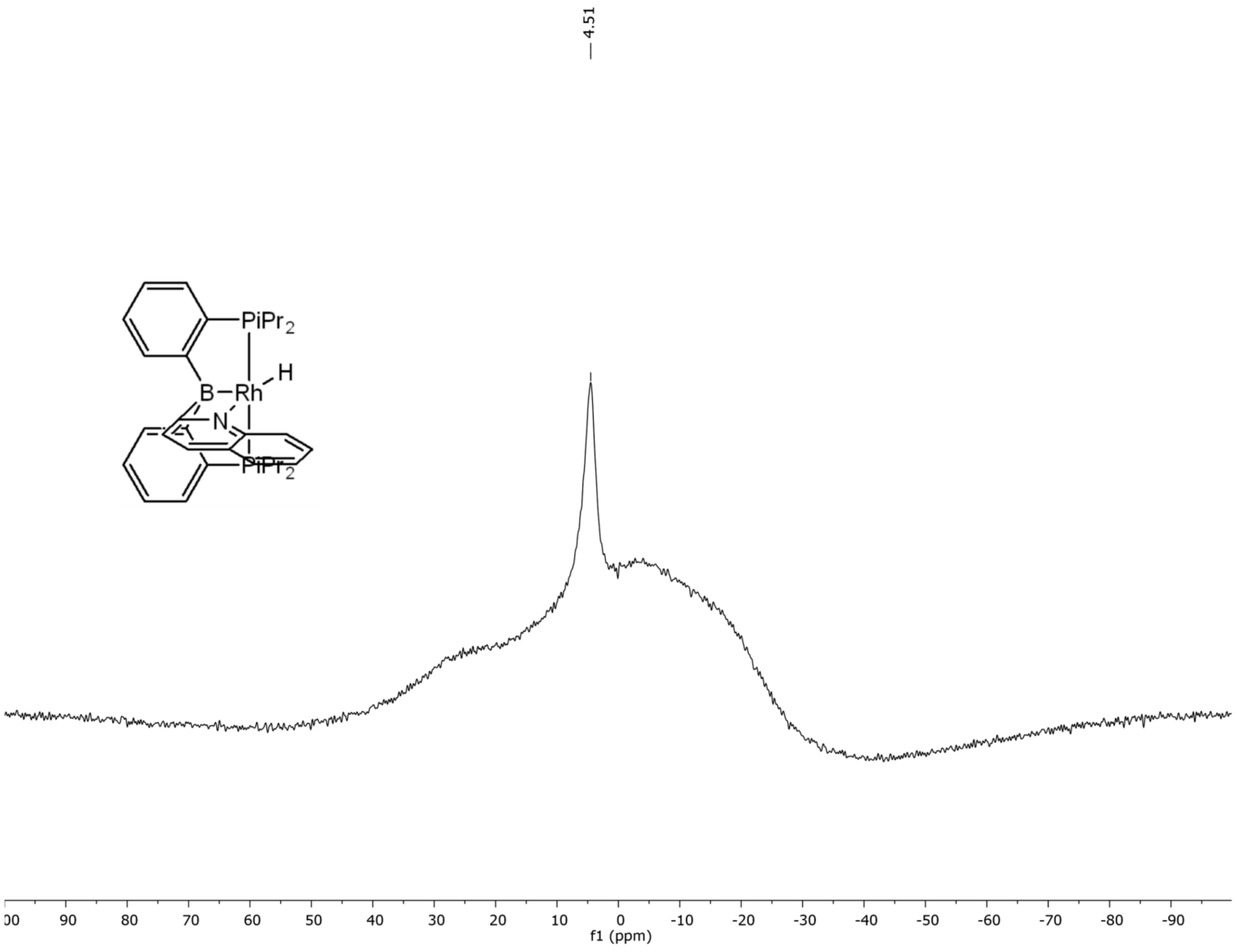


**Figure S12.** $^{11}B\{^{1}H\}$ NMR spectrum of **1RhNq** in $C_6D_6$ measured on a 400 MHz Varian NMR

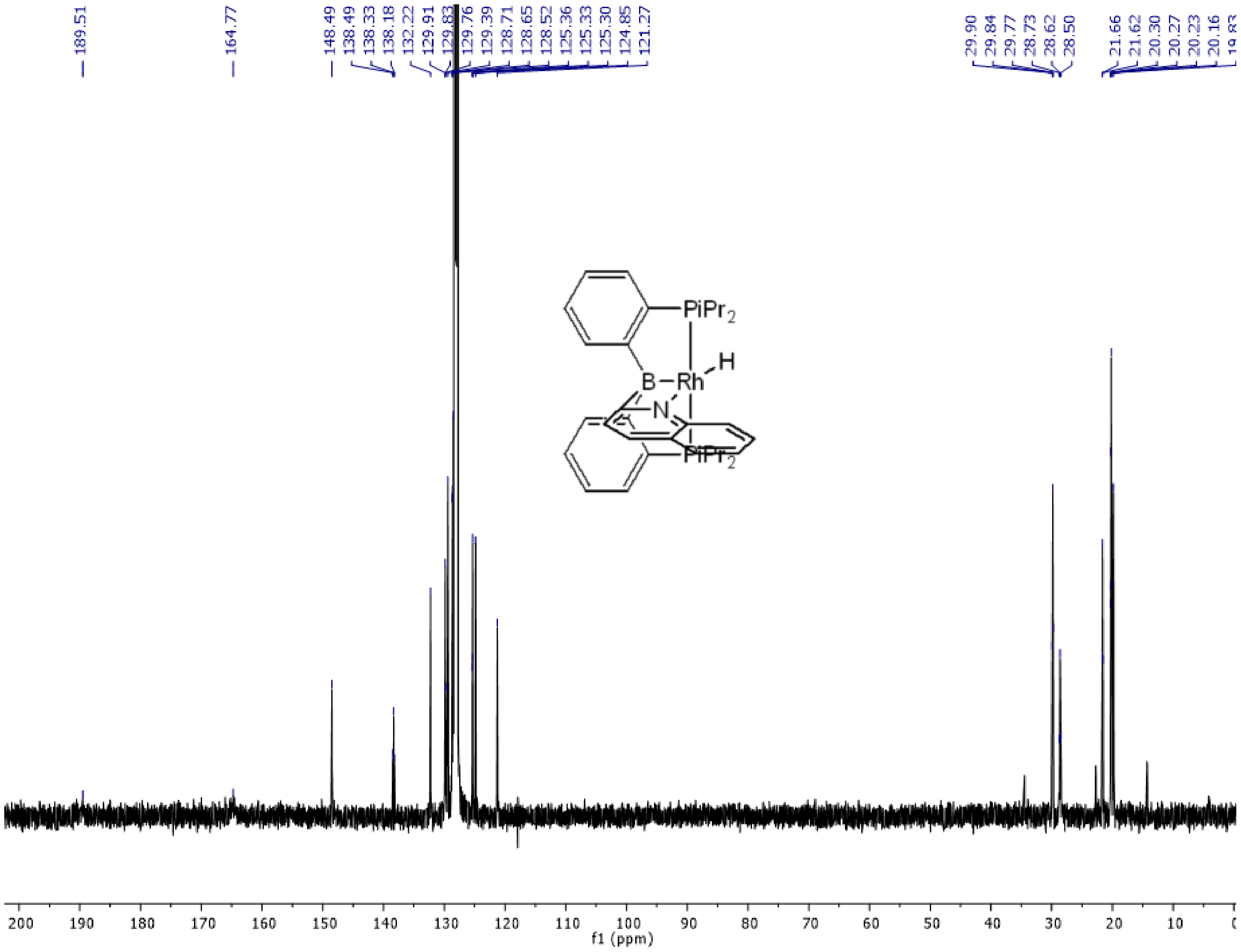

**Figure S13.** $^{13}C\{^1H\}$ NMR of **1RhNq** in $C_6D_6$ measured on a 500 MHz Varian NMR

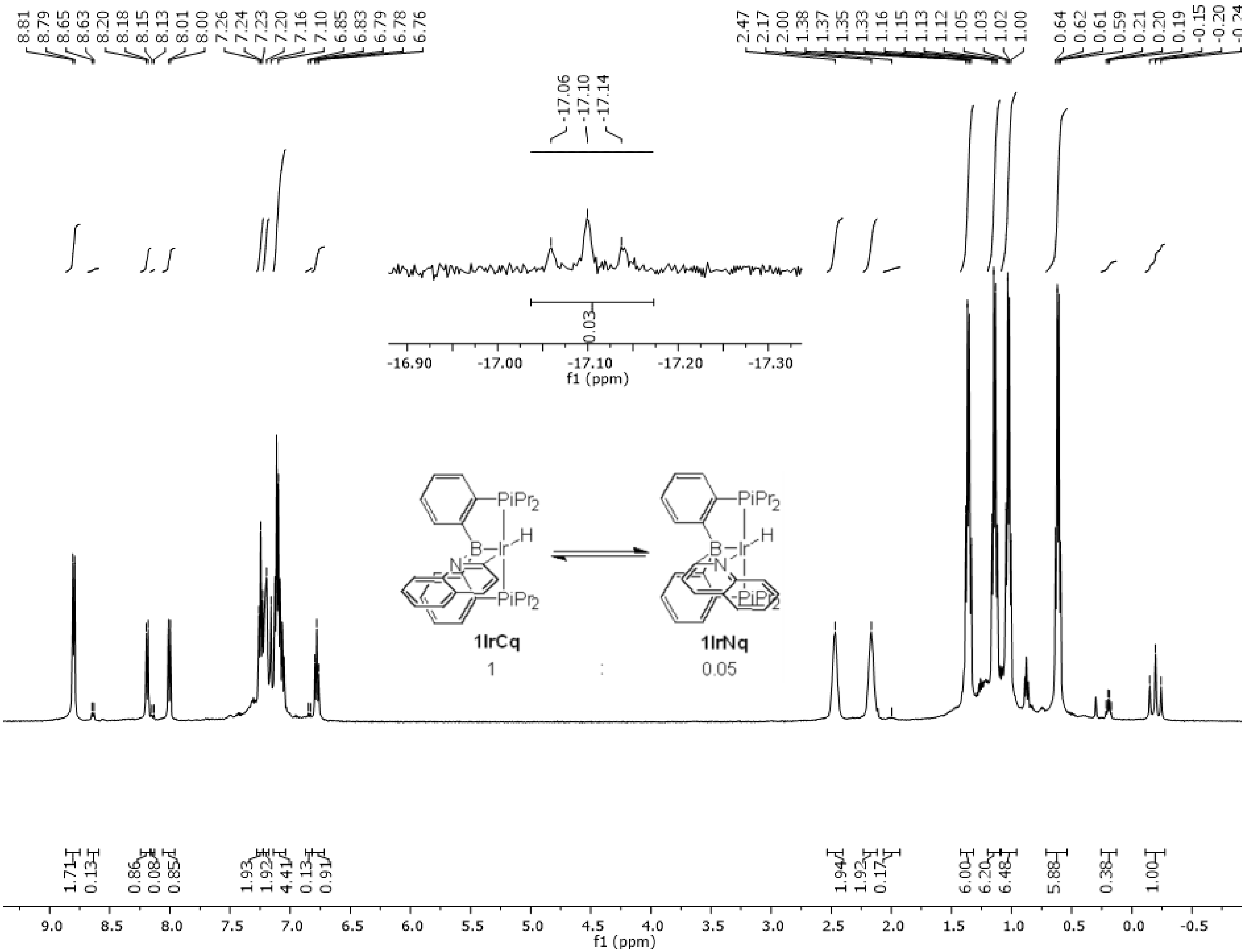


**Figure S14.** $^{1}H$ NMR spectrum of **1IrCq/1IrNq** in $C_6D_6$ measured on a 500 MHz Varian NMR

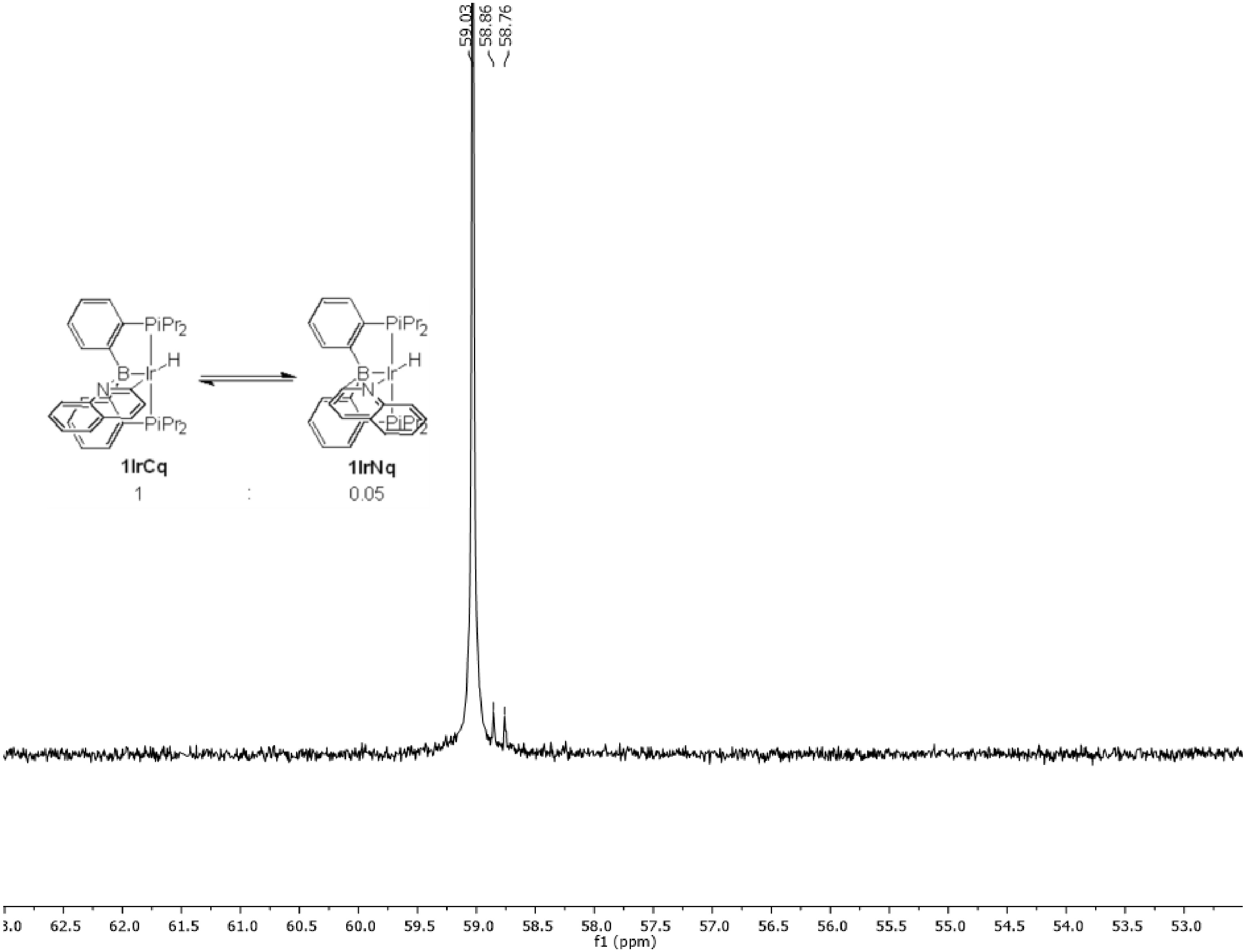


**Figure S15.** $^{31}P$ NMR spectrum (with selected $^{1}H$ decoupled) of **1IrCq/1IrNq** in $C_6D_6$ measured on a 500 MHz Varian NMR

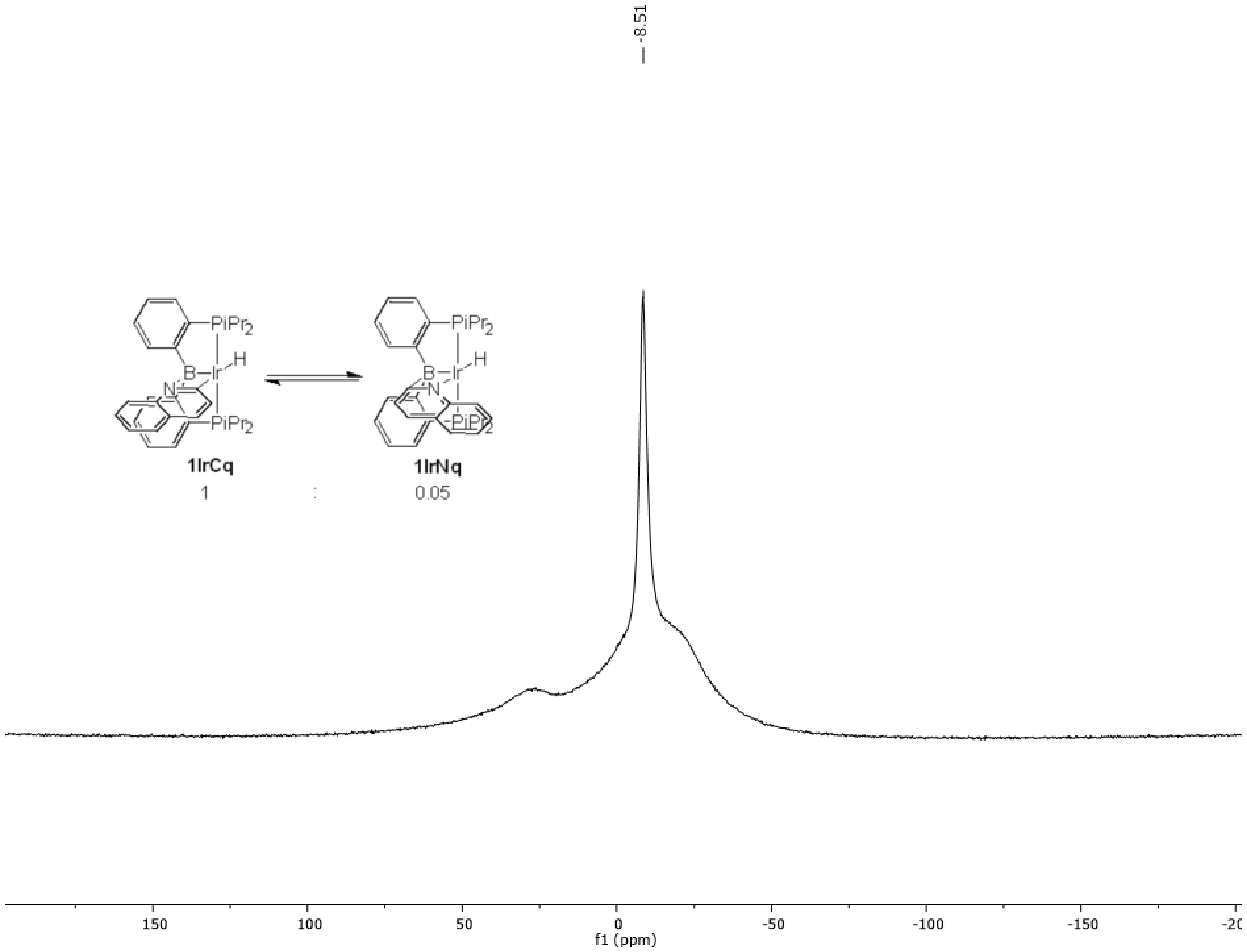


**Figure S16.** $^{11}B\{^{1}H\}$ NMR spectrum of **1IrCq**/**1IrNq** in $C_6D_6$ measured on a 400 MHz Bruker NMR

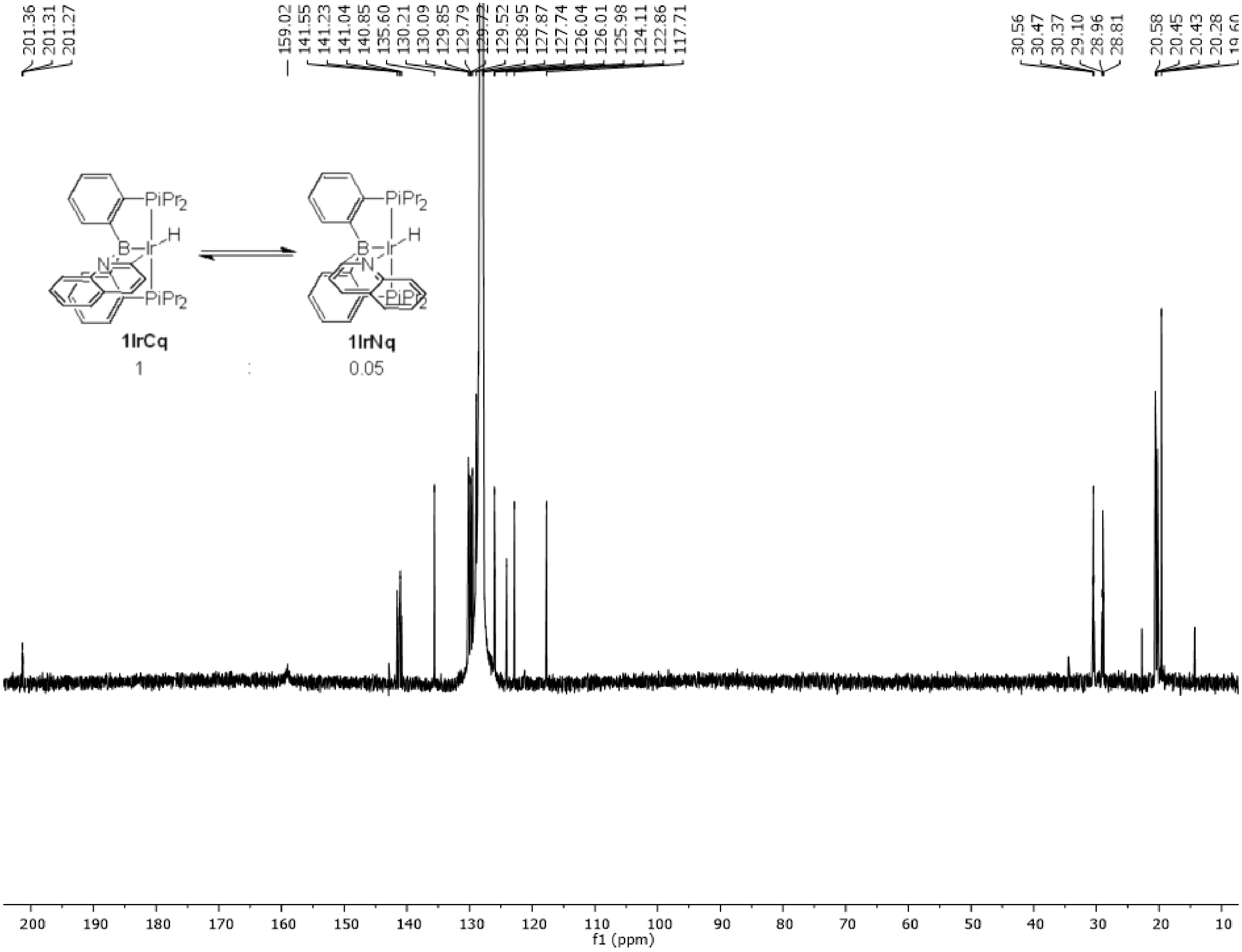


**Figure S17.** $^{13}C\{^1H\}$ NMR spectrum of **1IrCq**/**1IrNq** in $C_6D_6$ measured on a 500 MHz Varian NMR

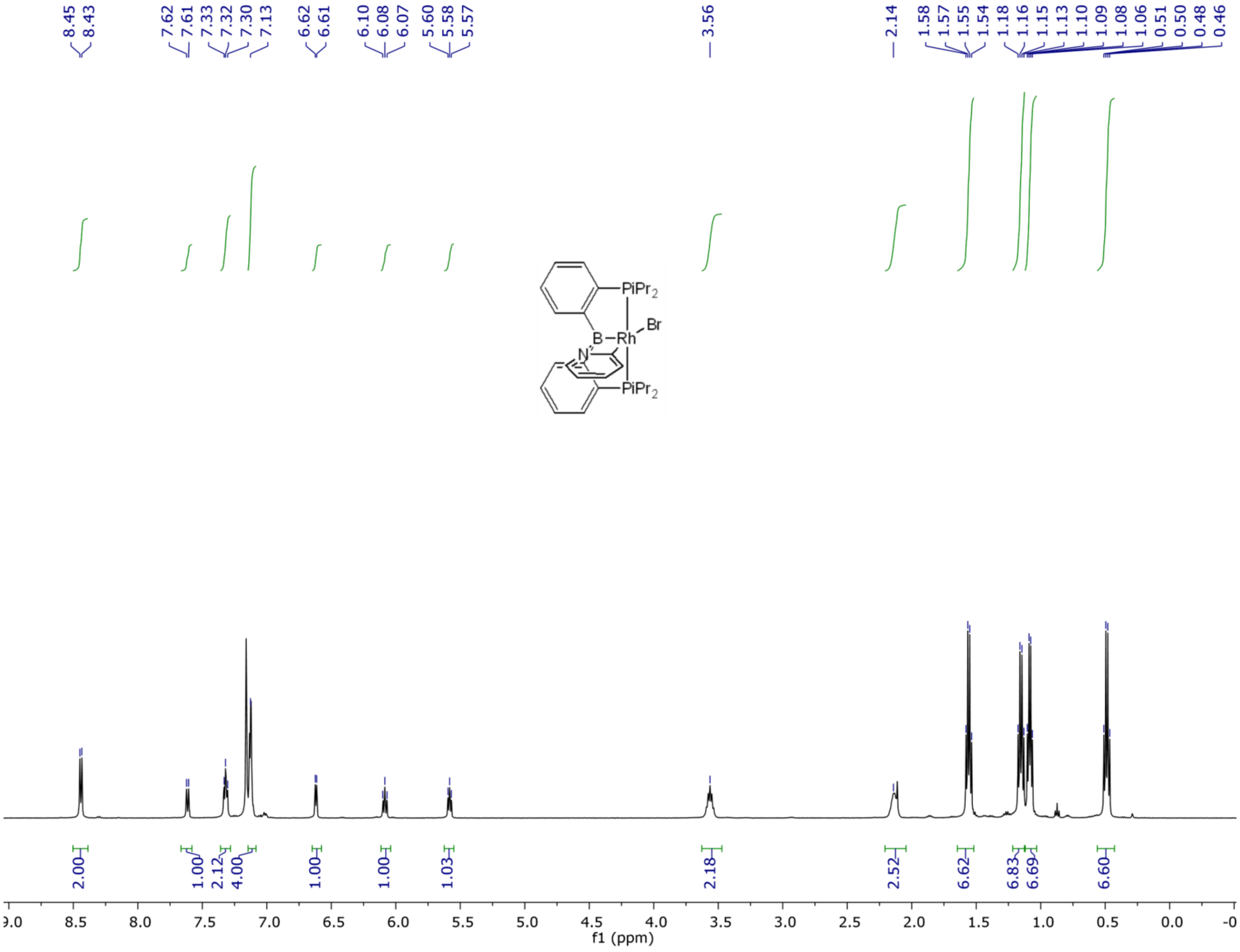


**Figure S18.** $^{1}$H NMR spectrum of **2RhC** in $C_6D_6$ measured on a 500 MHz Varian NMR

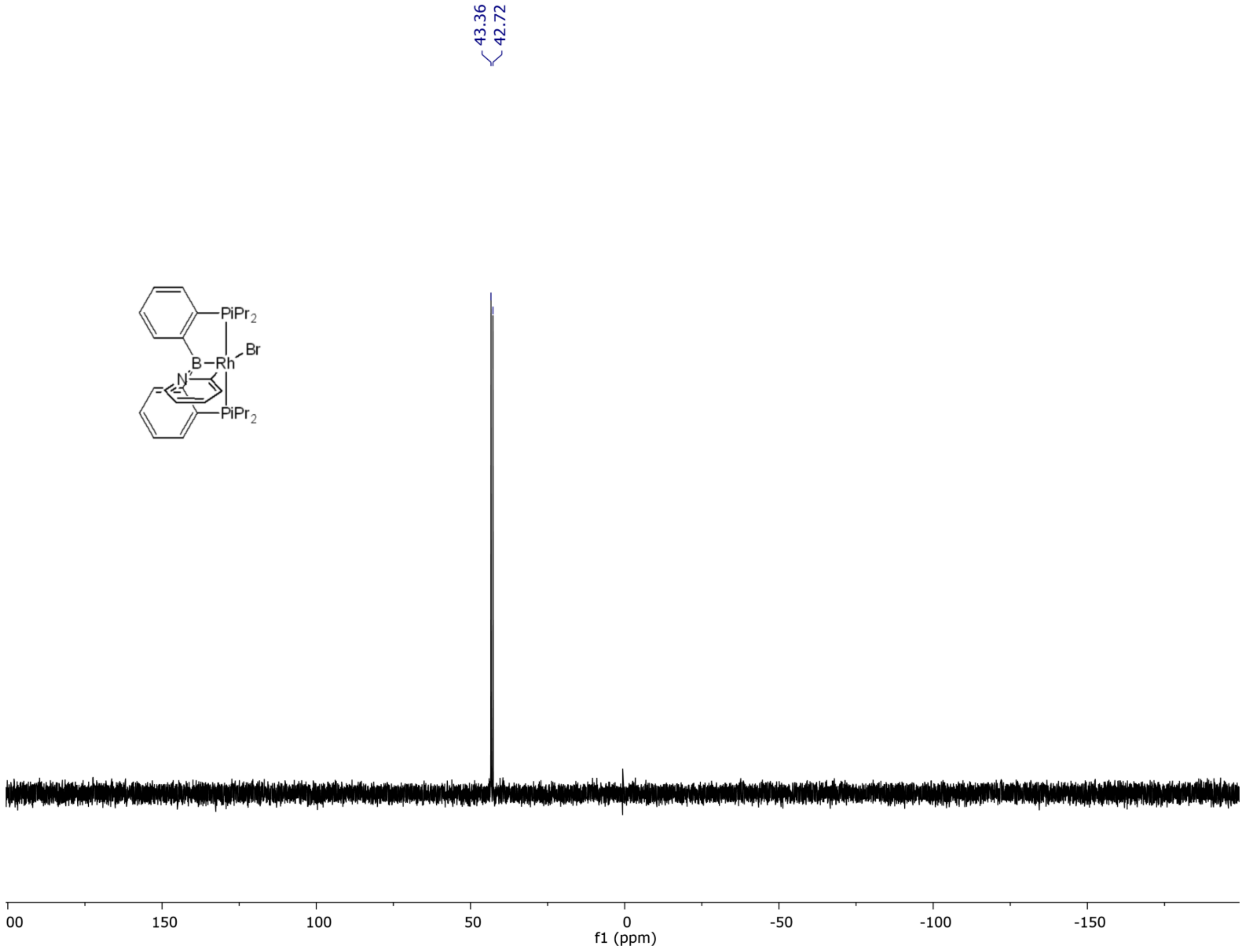


**Figure S19.** $^{31}P\{^1H\}$ NMR spectrum of **2RhC** in $C_6D_6$ measured on a 500 MHz Varian NMR

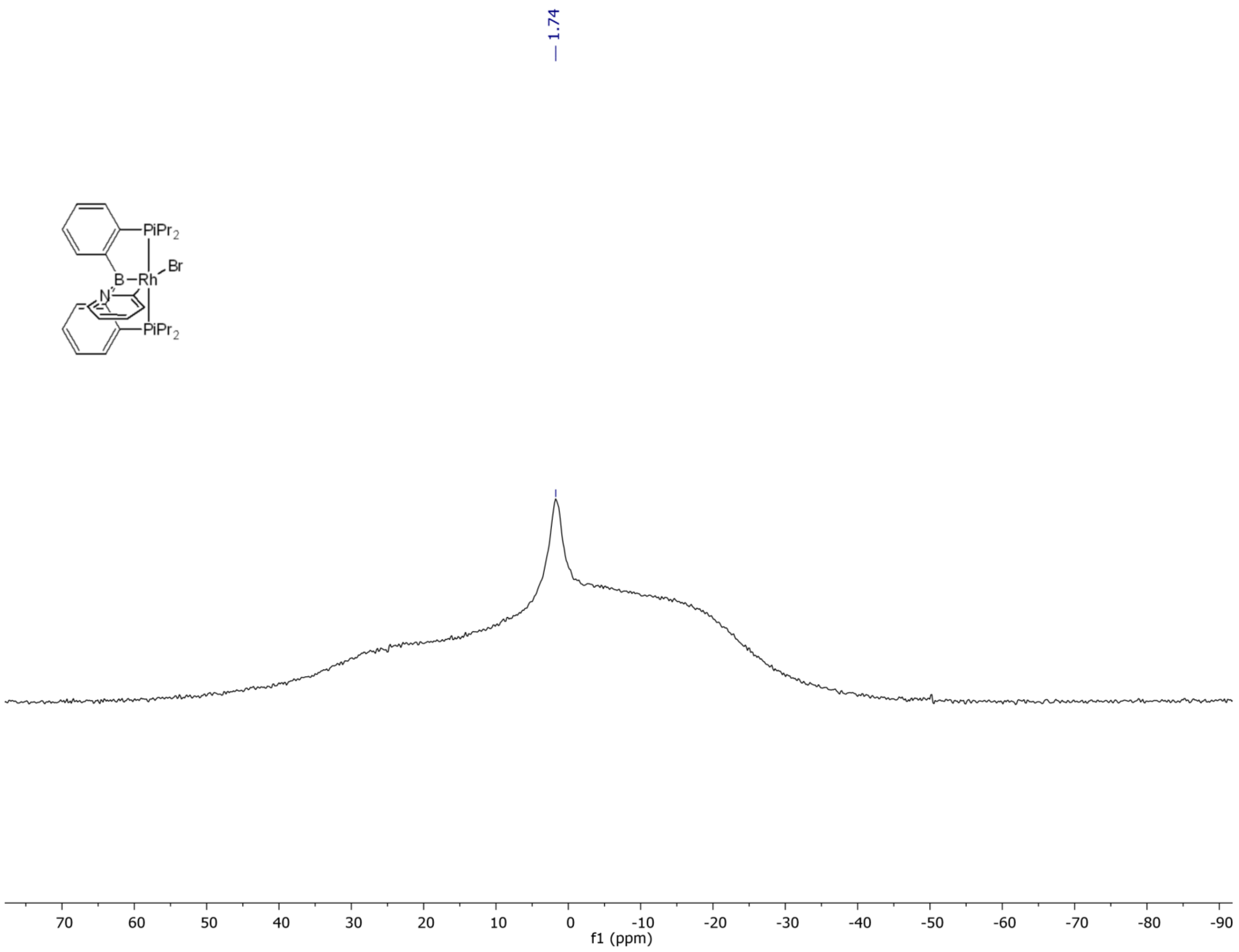


**Figure S20.** $^{11}B\{^1H\}$ NMR spectrum of **2RhC** in $C_6D_6$ measured on a 400 MHz Varian NMR

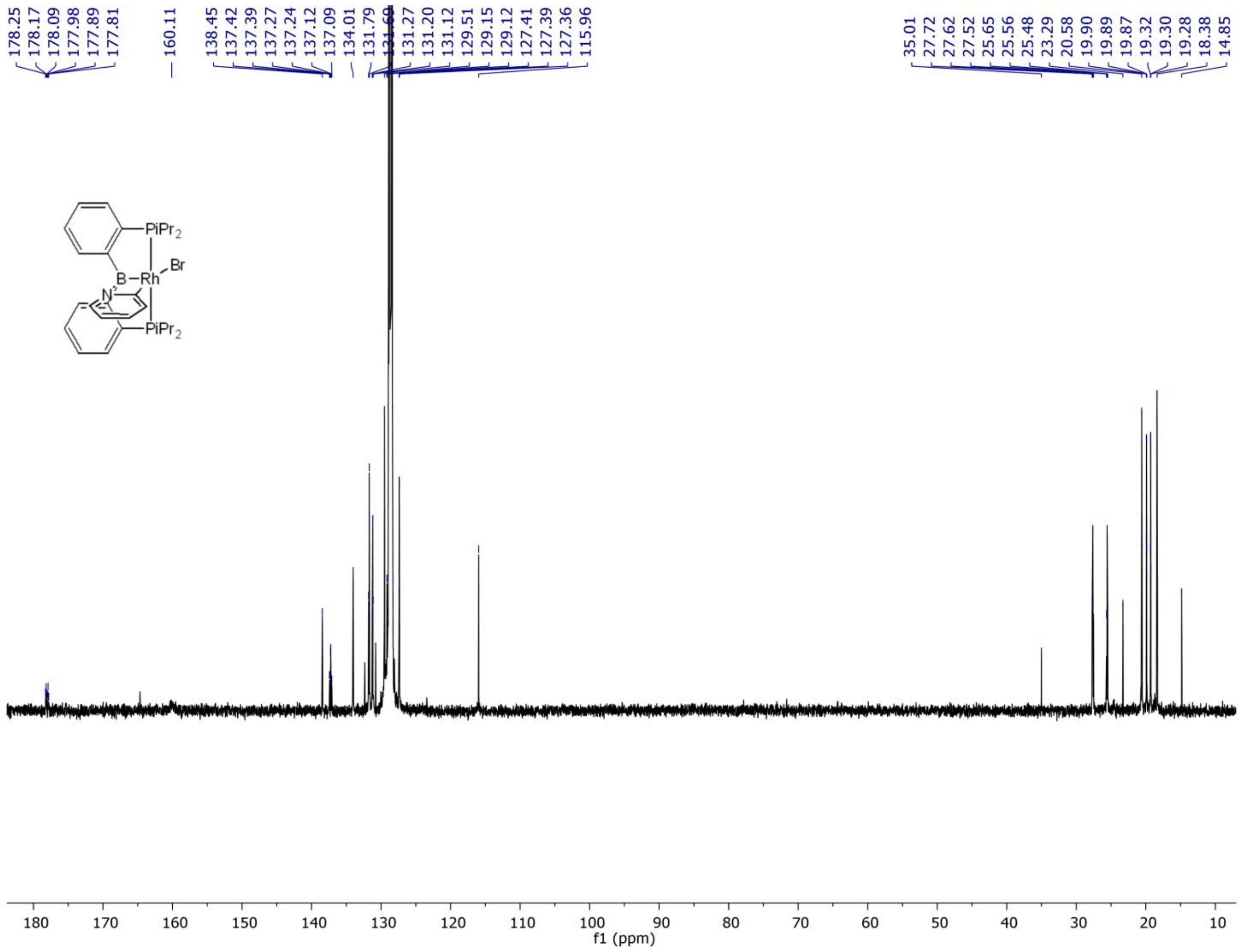


**Figure S21.** $^{13}C\{^{1}H\}$ NMR of **2RhC** in $C_6D_6$ measured on a 500 MHz Varian NMR.

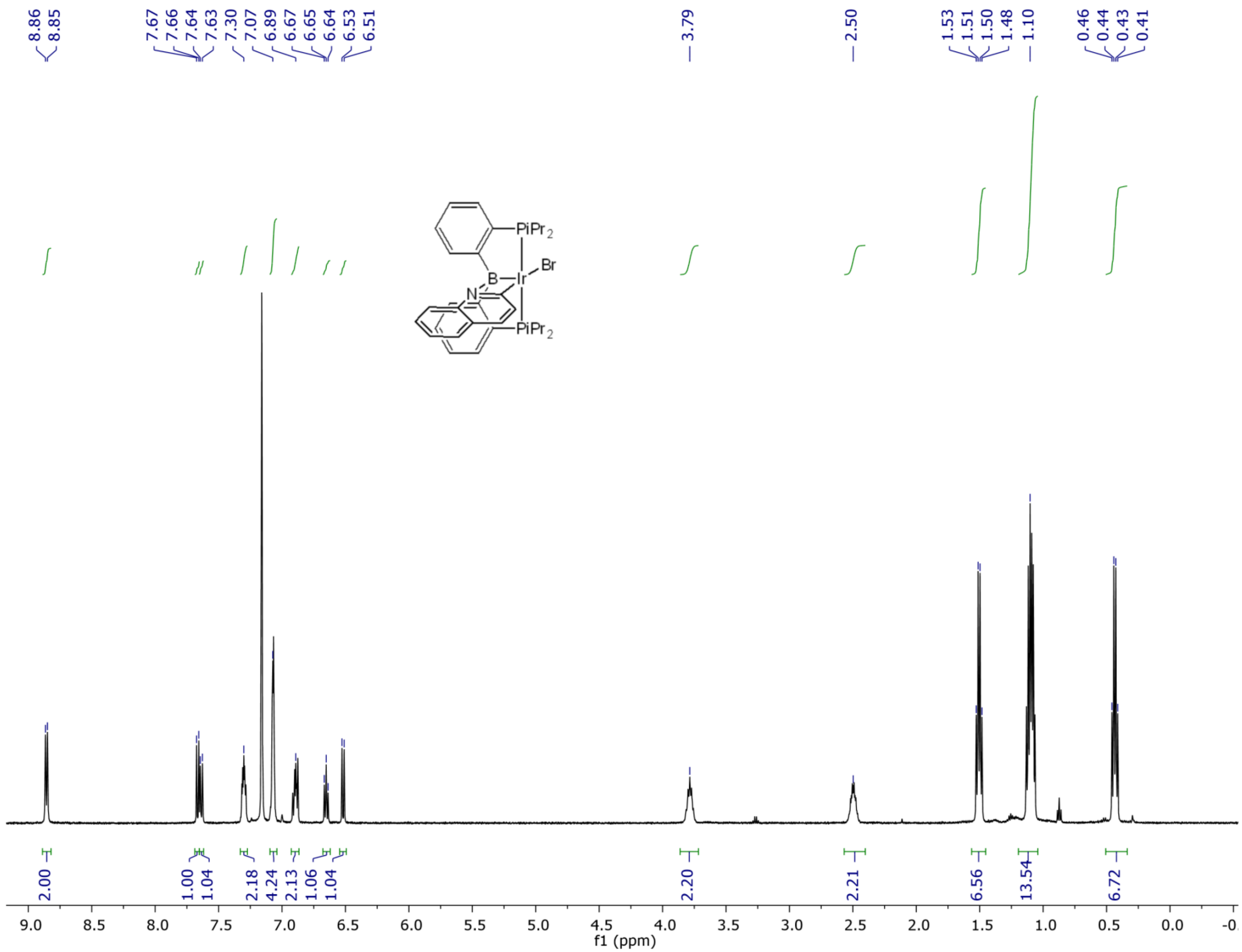

**Figure S22.** $^{1}$H NMR spectrum of **2IrCq** in $C_6D_6$ measured on a 500 MHz Varian NMR

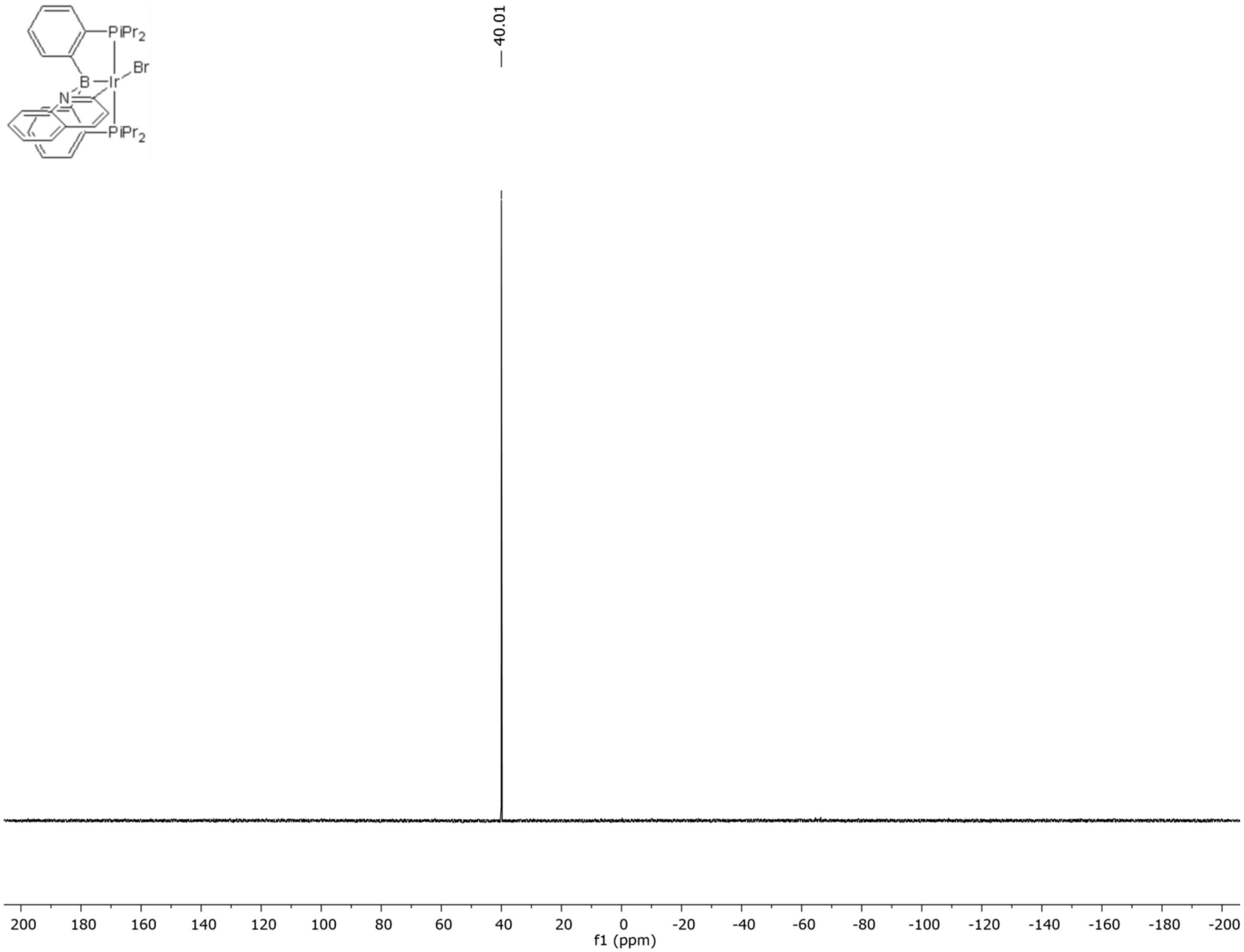


**Figure S23.** $^{31}P\{^1H\}$ NMR spectrum of **2IrCq** in $CDCl_3$ measured on a 500 MHz Varian NMR

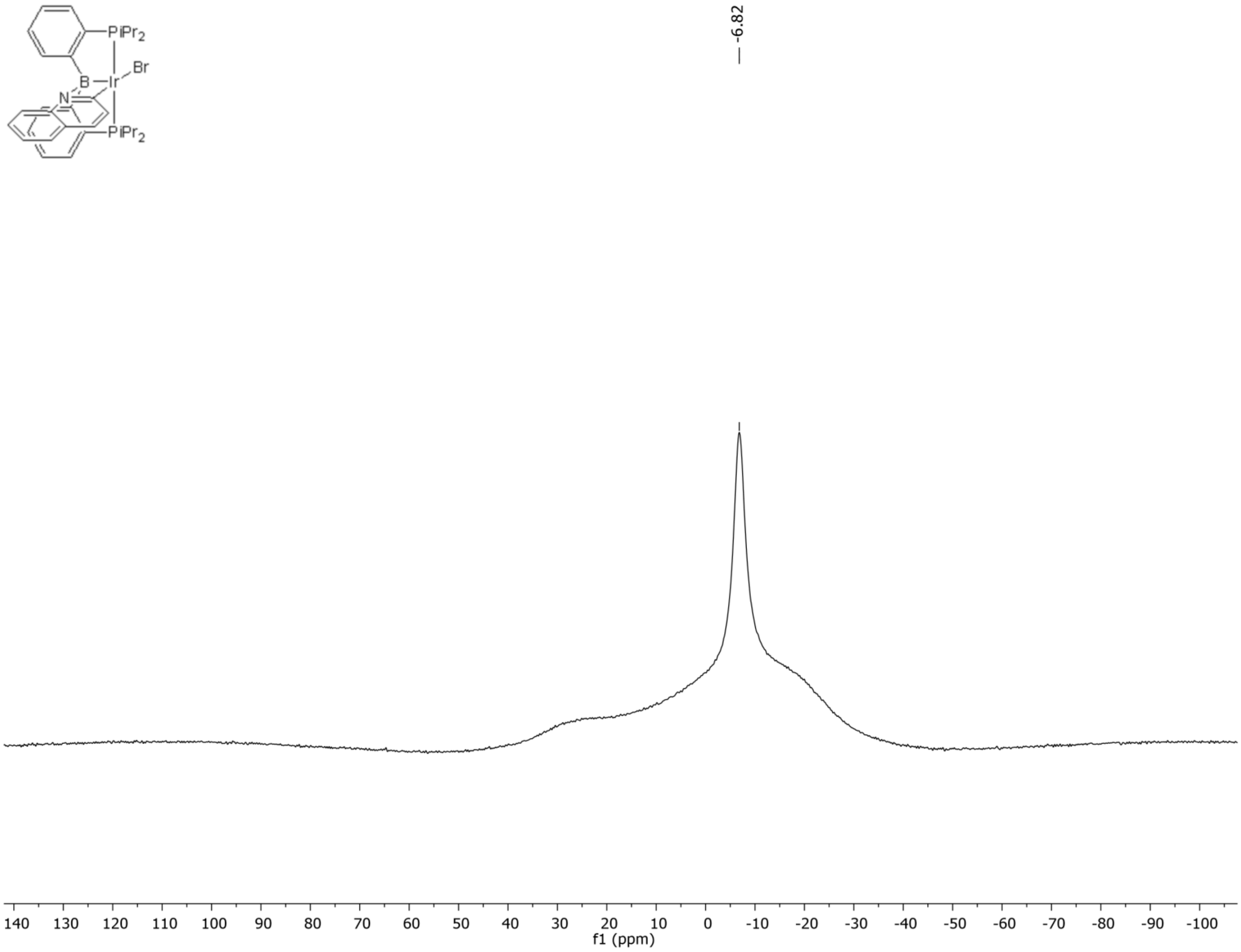


**Figure S24.** $^{11}B\{^1H\}$ NMR spectrum of **2IrCq** in $CDCl_3$ measured on a 400 MHz Varian NMR

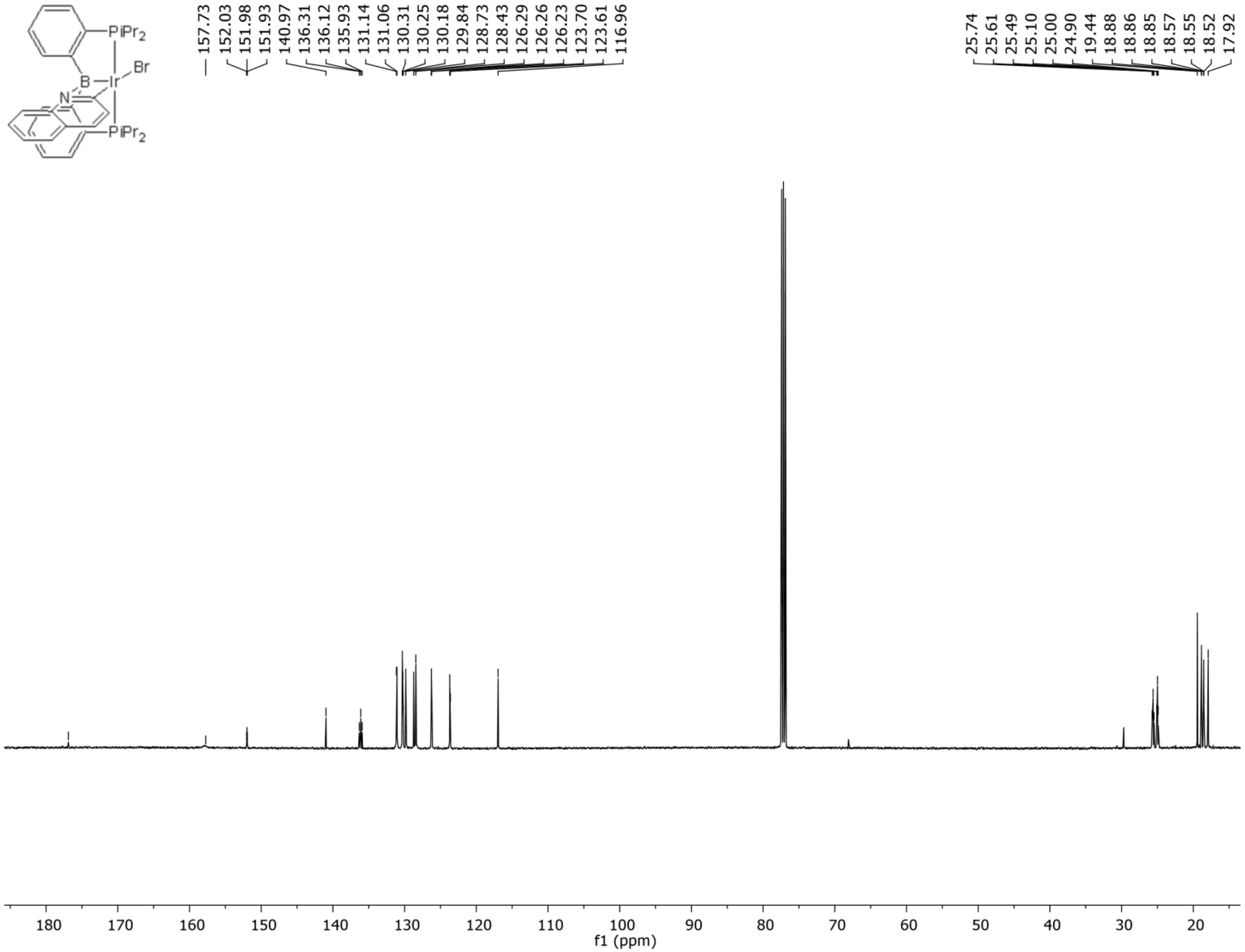


**Figure S25.** $^{13}C\{^{1}H\}$ NMR spectrum of **2IrCq** in $CDCl_3$ measured on a 500 MHz Varian NMR

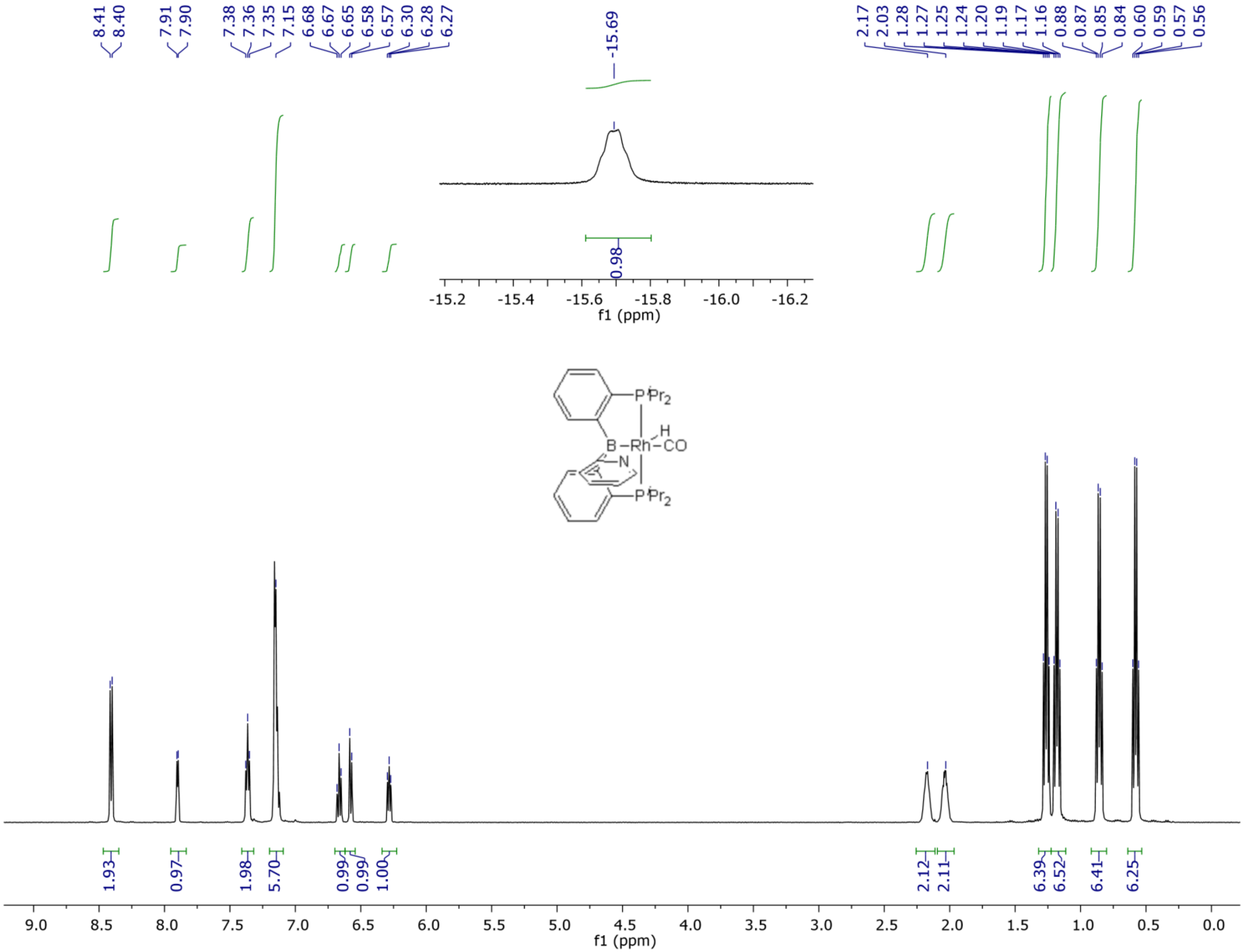


**Figure S26.** $^1$H NMR spectrum of **3RhN** in $C_6D_6$ measured on a 500 MHz Varian NMR

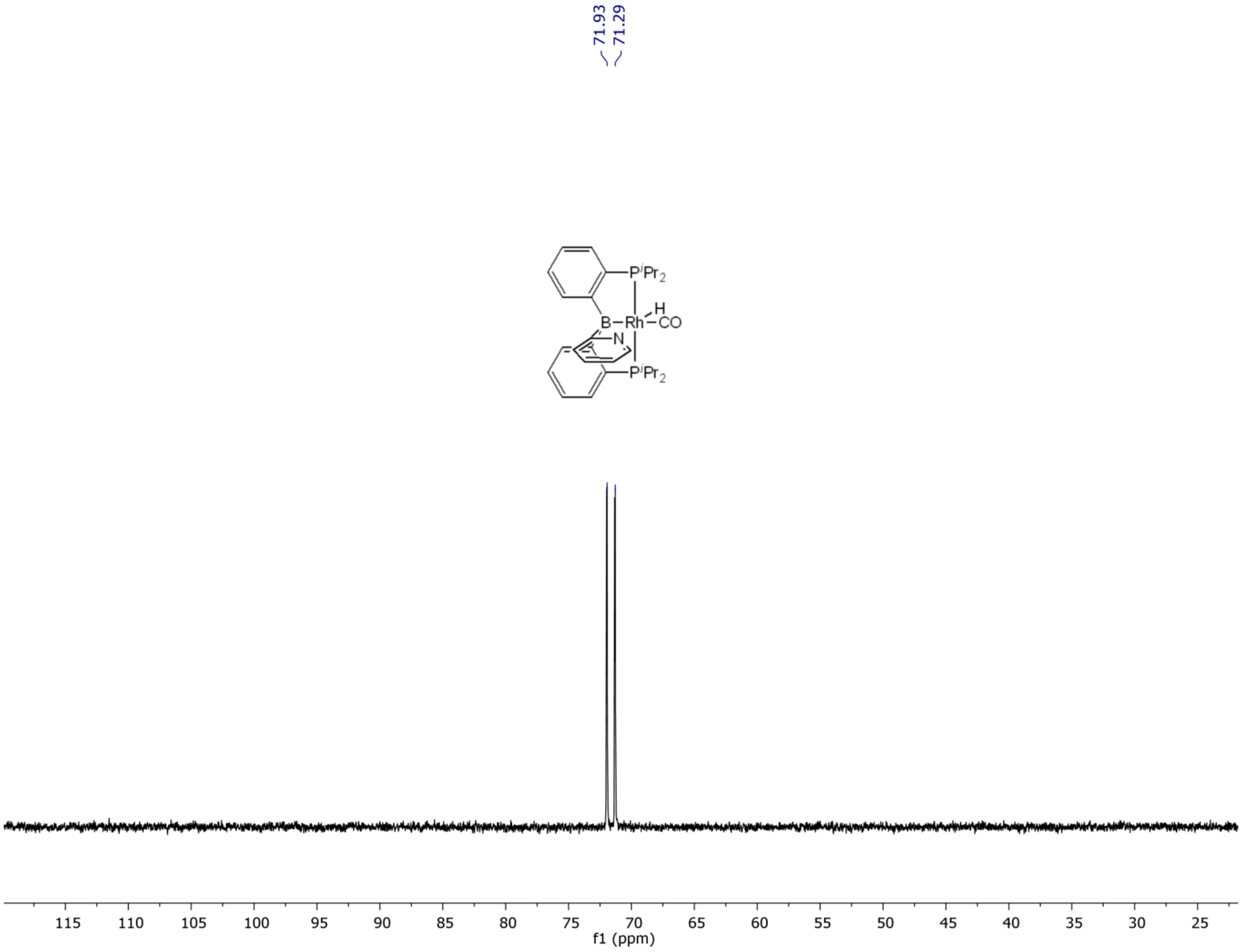


**Figure S27.** $^{31}P\{^1H\}$ NMR spectrum of **3RhN** in $C_6D_6$ measured on a 500 MHz Varian NMR

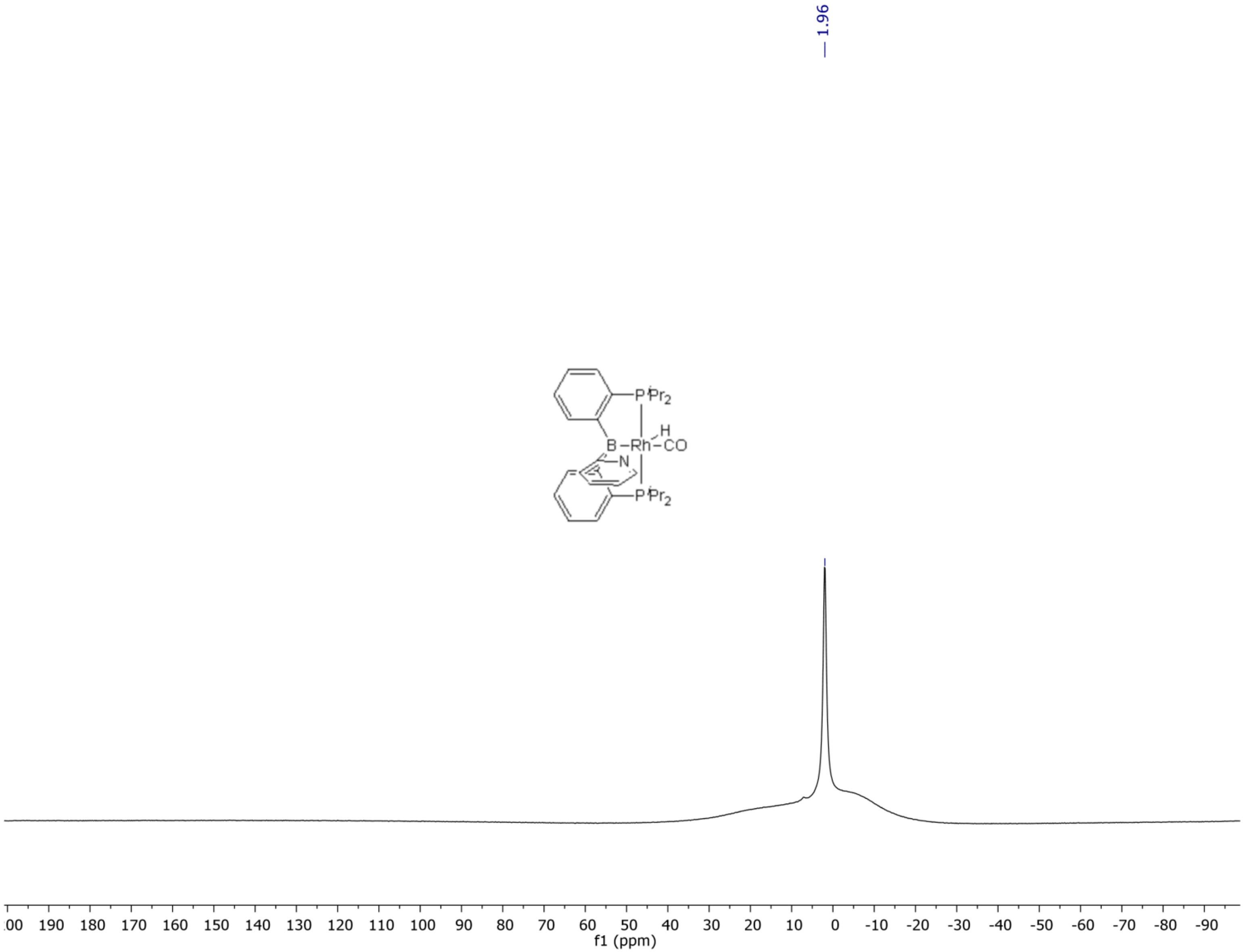


**Figure S28.** $^{11}B\{^1H\}$ NMR spectrum of **3RhN** in $C_6D_6$ measured on a 500 MHz Bruker NMR

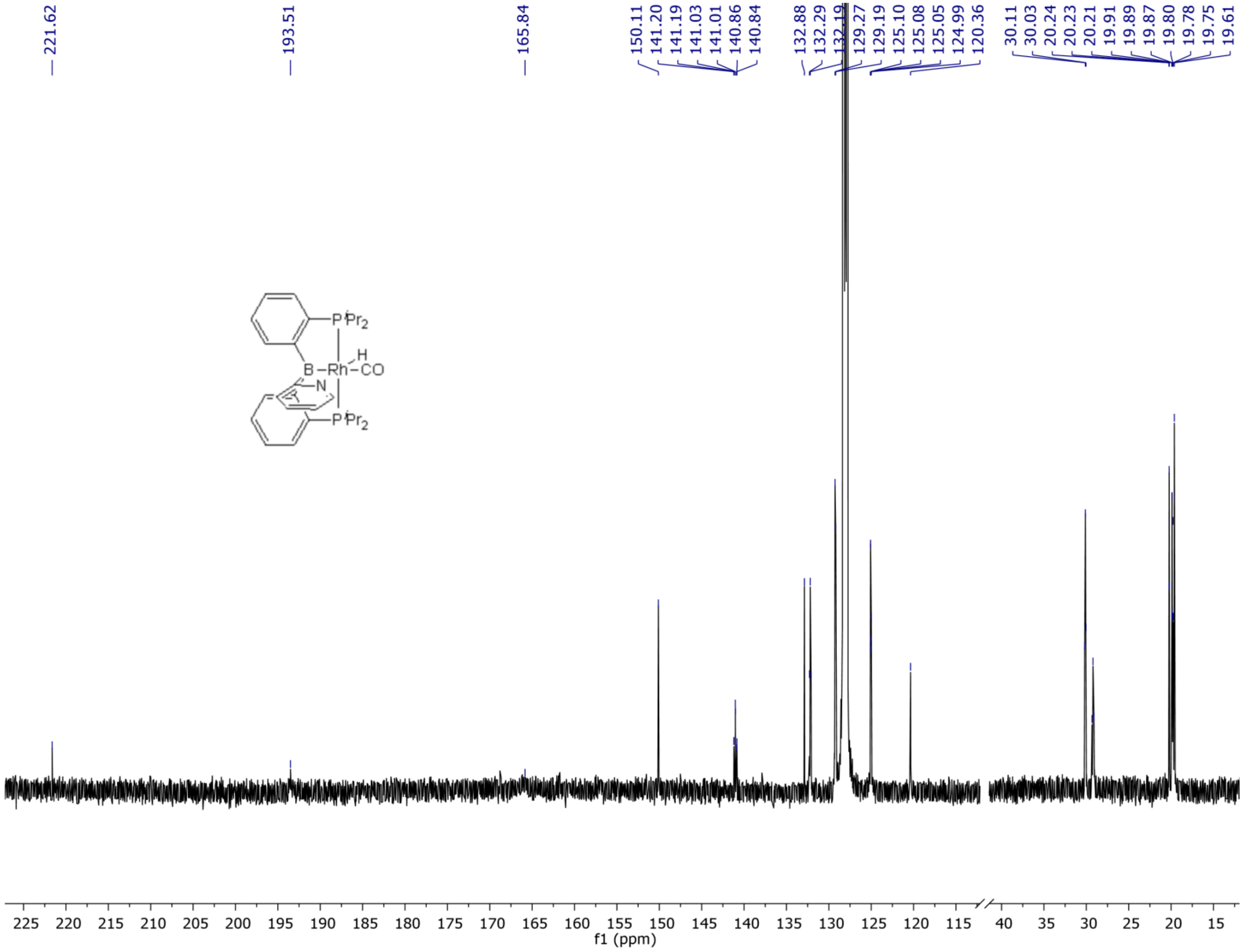


**Figure S29.** $^{13}C\{^1H\}$ NMR spectrum of **3RhN** in $C_6D_6$ measured on a 500 MHz Varian NMR

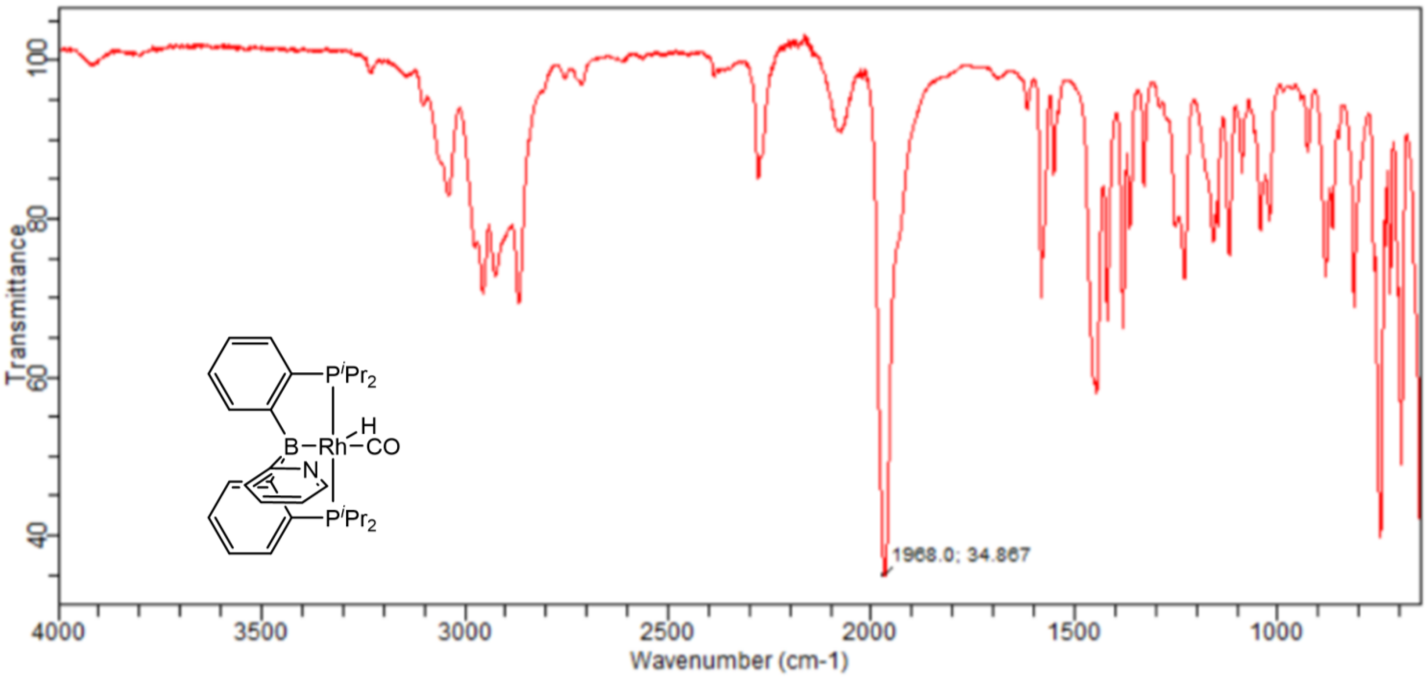


**Figure S30.** IR spectrum of **3RhN** measured on an Agilent ATR-IR spectrometer.

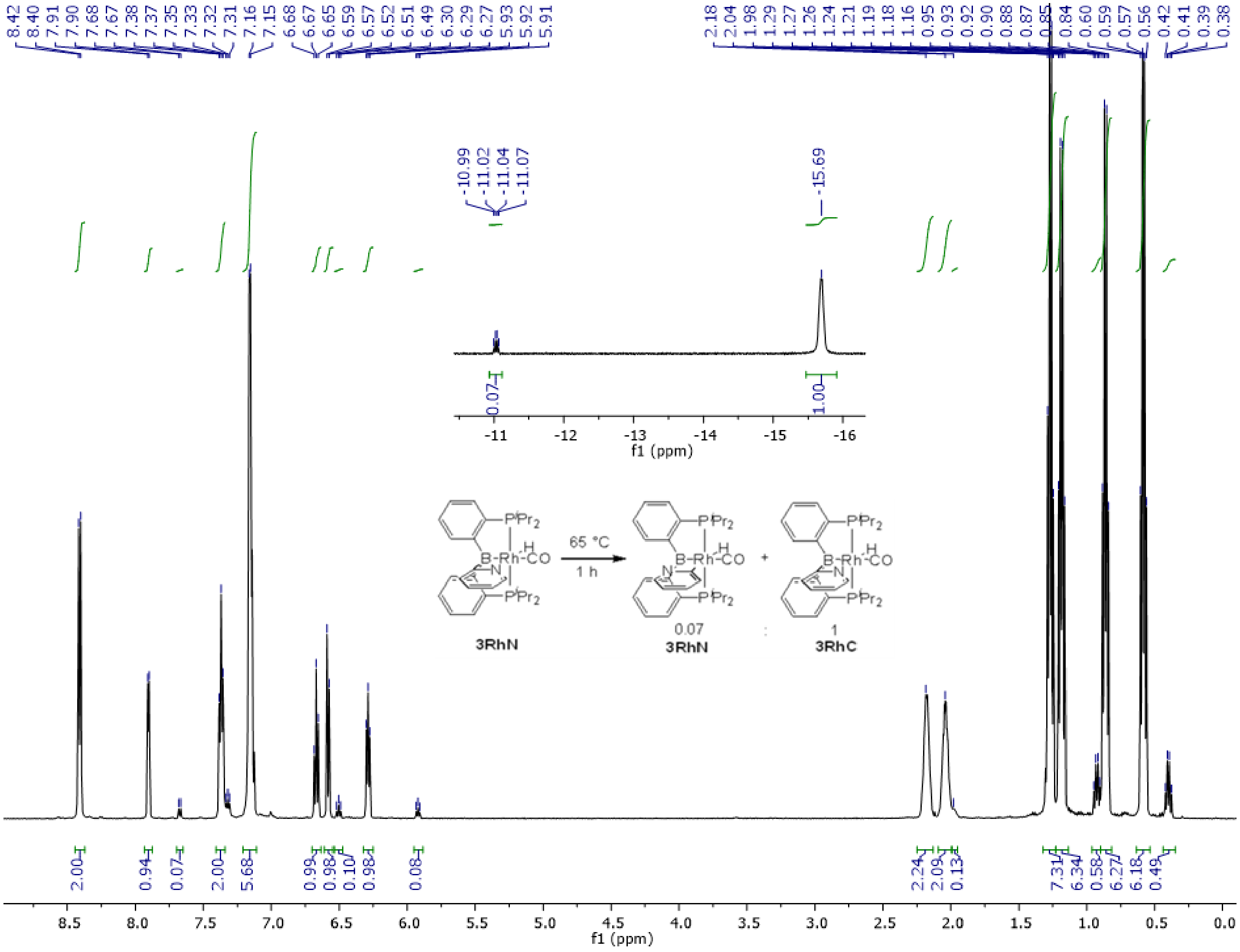

**Figure S31.** $^{1}H$ NMR spectrum of **3RhN** in $C_6D_6$ measured at room temperature on a 500 MHz Varian NMR after heating at 65 °C for 1 h. The sample contains a mixture of **3RhN**/**3RhC** (1:0.07)

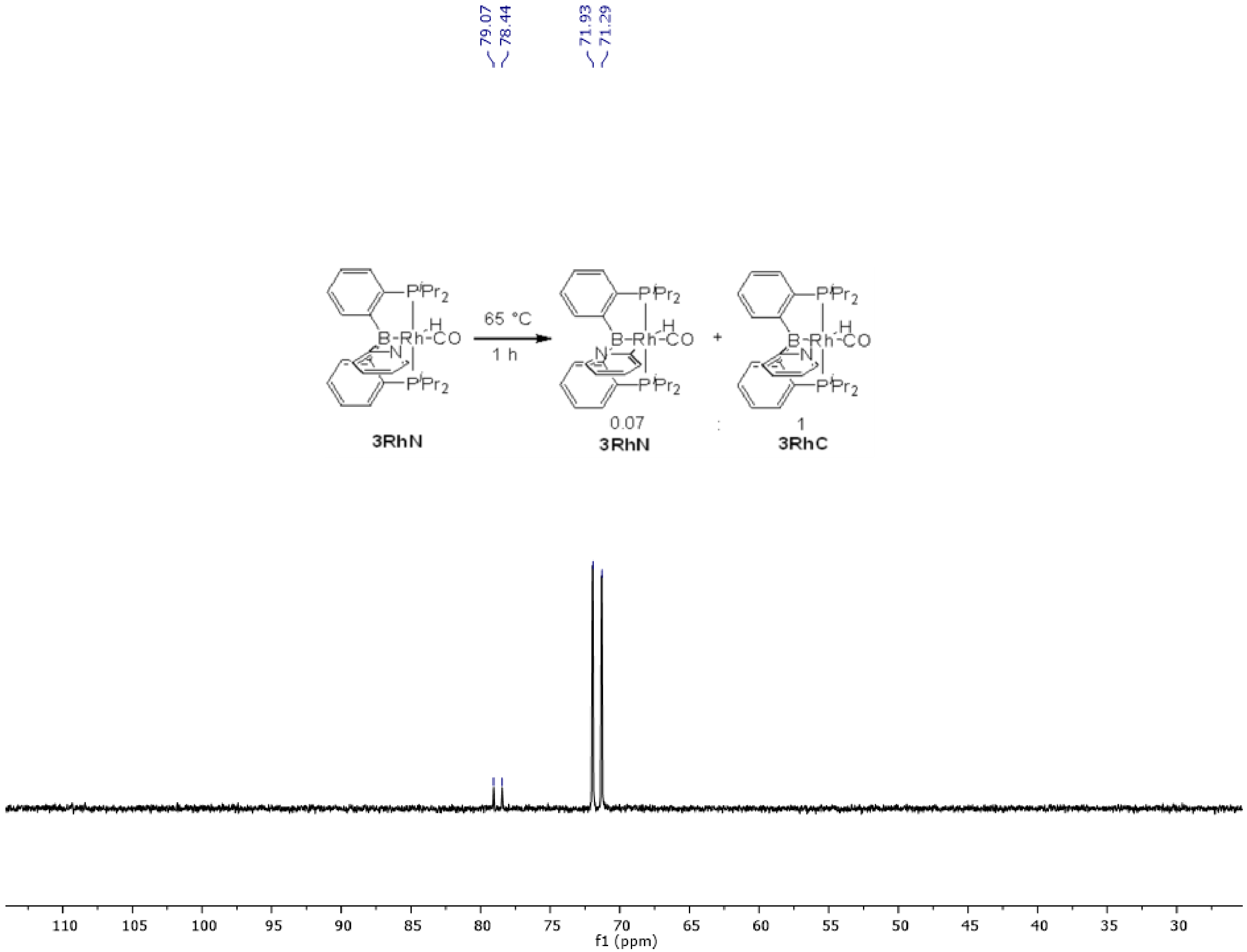


**Figure S32.** $^{31}P\{^1H\}$ NMR spectrum of **3RhN** in $C_6D_6$ measured at room temperature on a 500 MHz Varian NMR after heating at 65 °C for 1 h. The sample contains a mixture of **3RhN**/**3RhC** (1:0.07)

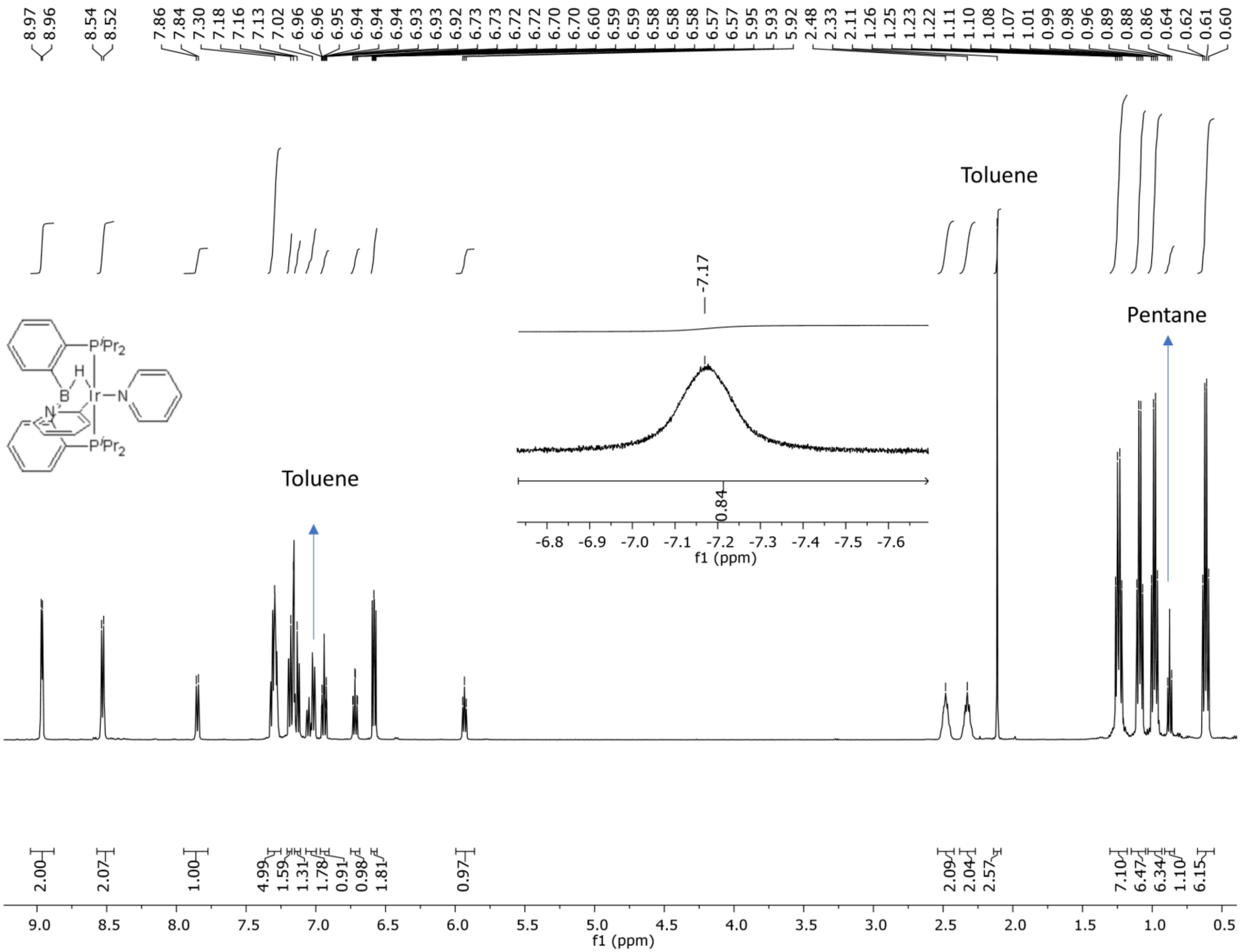


**Figure S33.** $^1$H NMR spectrum of **7** in $C_6D_6$ measured on a 500 MHz Varian NMR

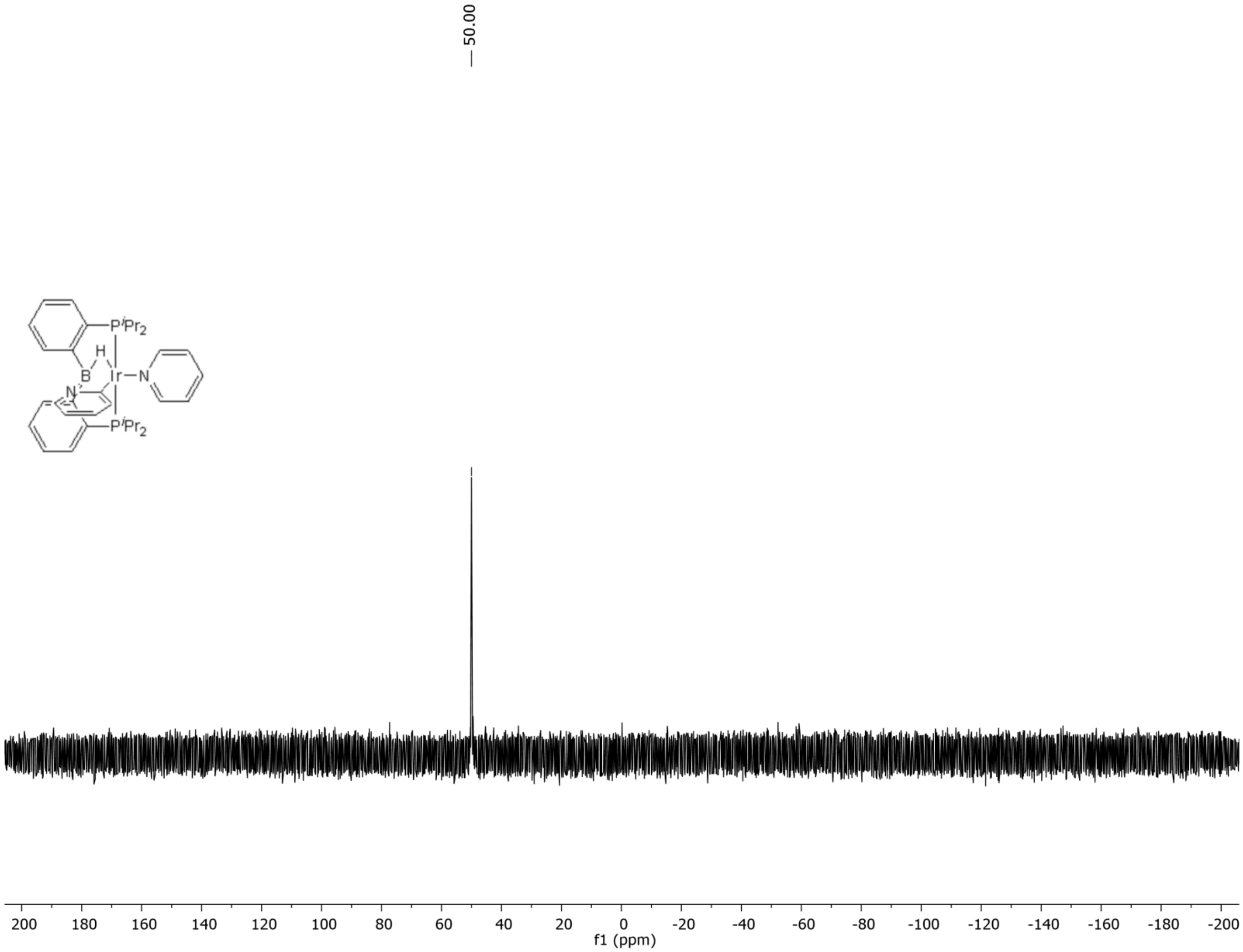


**Figure S34.** $^{31}P\{^1H\}$ NMR spectrum of **7** in $C_6D_6$ measured on a 500 MHz Varian NMR

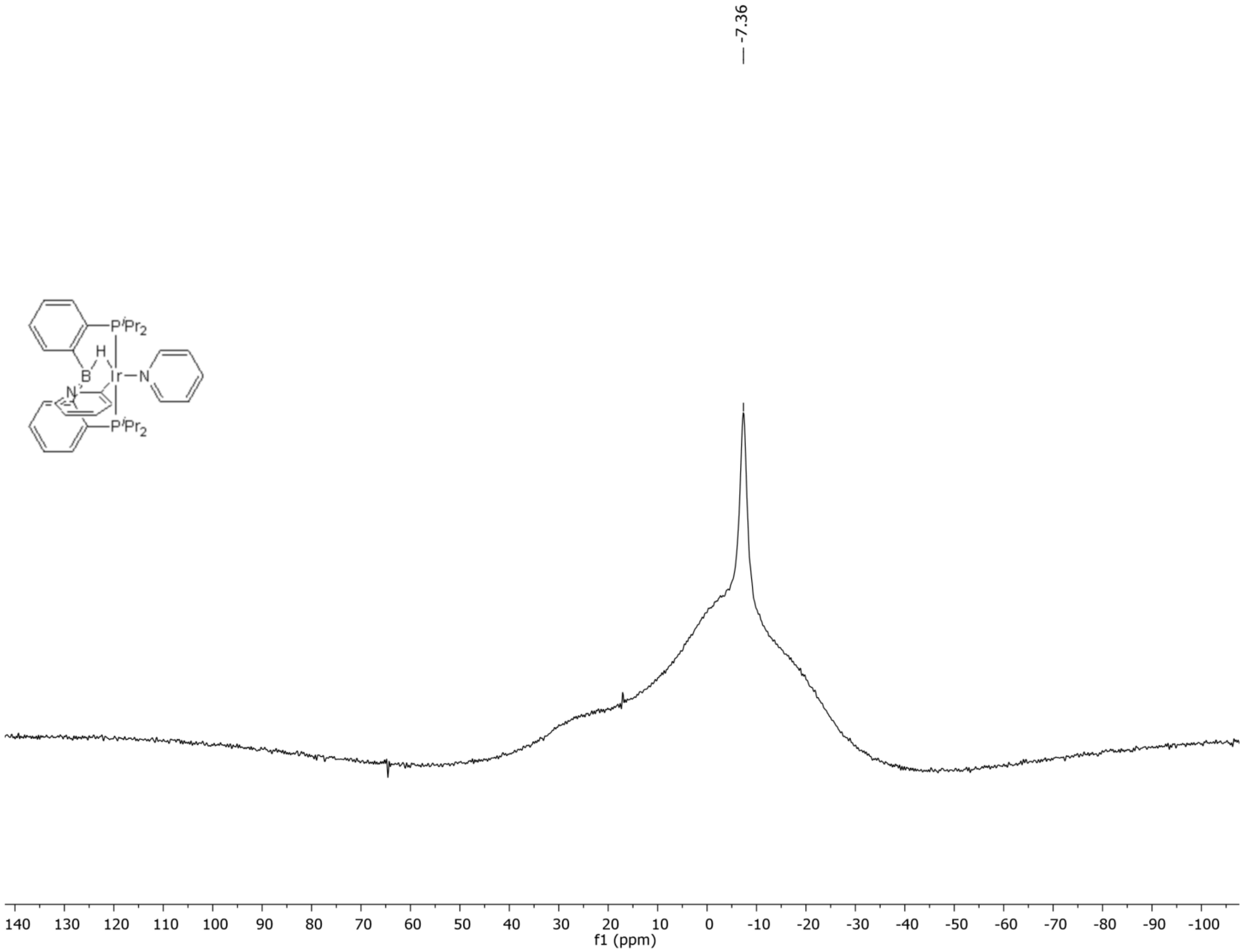


**Figure S35.** $^{11}B\{^{1}H\}$ NMR spectrum of **7** in $C_6D_6$ measured on a 400 MHz Bruker NMR

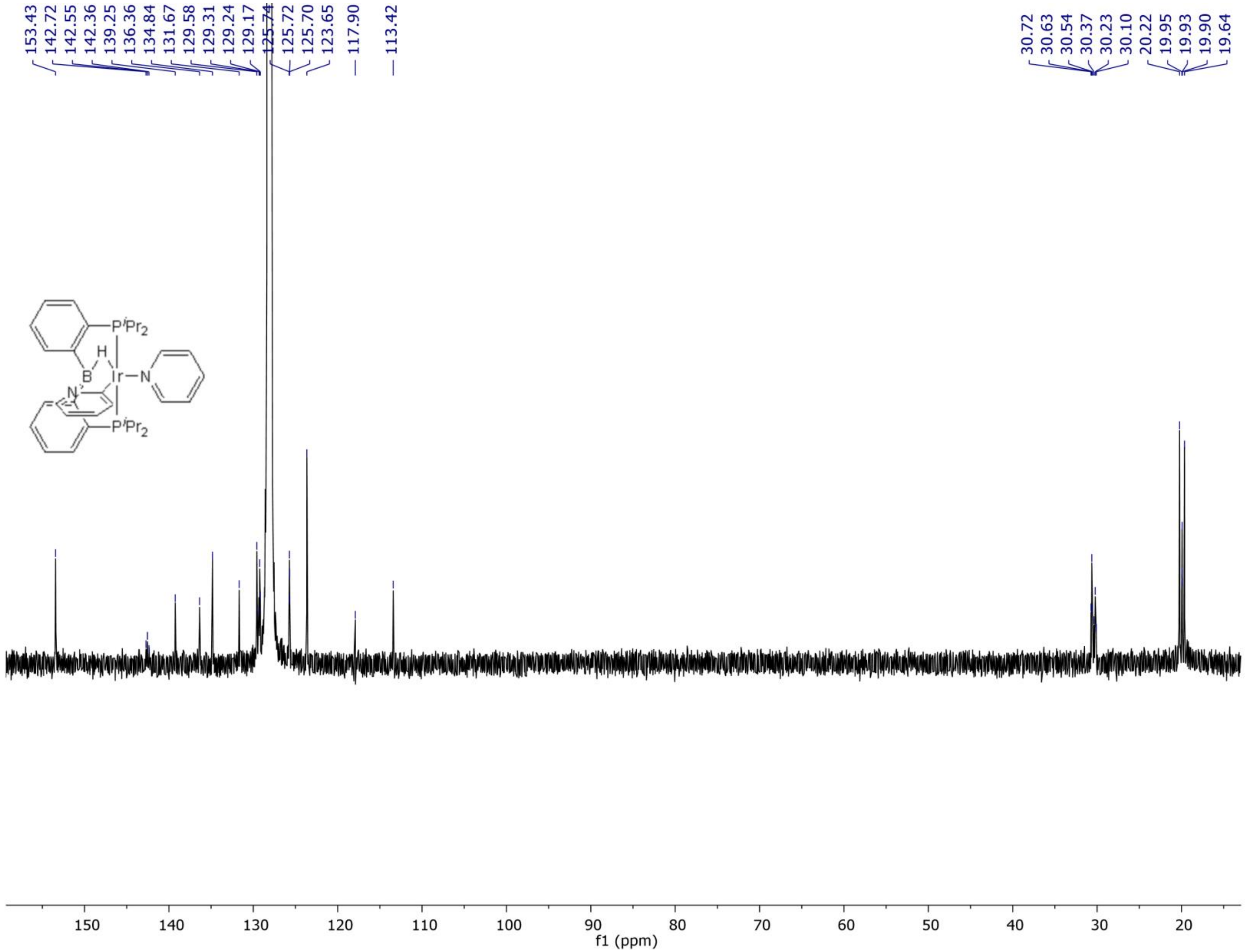


**Figure S36.** $^{13}C\{^1H\}$ NMR spectrum of **7** in $C_6D_6$ measured on a 500 MHz Varian NMR

## 6. SI References.


[1] W.-C. Shih, W. Gu, M. C. MacInnis, S. D. Timpa, N. Bhuvanesh, J. Zhou, and O. V. Ozerov, *J. Am. Chem. Soc.,* 2016, **138**, 2086-2089.

[2] APEX2 *"Version 2 User Manual, M86-E01078"*, BRUKER AXS Inc., 5465 East Cheryl Parkway, Madison, WI 53711-5373 USA

[3] SADABS, Sheldrick, G.M. *"Program for Absorption Correction of Area Detector Frames"*,

BRUKER AXS Inc., 5465 East Cheryl Parkway, Madison, WI 53711-5373 USA.

[4] (a) G. M. Sheldrick, *Acta Cryst.,* 2008, **A64**, 112-122. (b) G. M. Sheldrick, *Acta Cryst.*, 2015, **A71**, 3-8. (c) G. M. Sheldrick, *Acta Cryst.*, 2015, **C71**, 3-8. (d) XT, XS, BRUKER AXS Inc., 5465 East Cheryl Parkway, Madison, WI 53711-5373 USA.

[5] A. L. Spek, *J. Appl. Crystallogr.,* 2003*,* **36***,* 7−13.

[6] APEX2 *"Program for Data Collection on Area Detectors"*, BRUKER AXS Inc., 5465 East Cheryl Parkway, Madison, WI 53711-5373 USA

[7] O. V. Dolomanov, L. J. Bourhis, R. J. Gildea, J. A. K. Howard, and H. Puschmann, H. *J. Appl. Cryst.,* 2009*,* **42**, 339-341.

[8] APEX3 *"Program for Data Collection on Area Detectors"* BRUKER AXS Inc., 5465 East Cheryl Parkway, Madison, WI 53711-5373 USA

[9] L. J. Farrugia, *J. Appl. Cryst.,* 2012, **45**, 849.

[10] POV-Ray Home page, http://www.povray.org/, (accessed December 2, 2020).

[11] M. J. Frisch, G. W. Trucks, H. B. Schlegel, G. E. Scuseria, M. A. Robb, J. R. Cheeseman, G. Scalmani, V. Barone, B. Mennucci, G. A. Petersson, H. Nakatsuji, M. Caricato, X. Li, H. P.

Hratchian, A. F. Izmaylov, J. Bloino, G. Zheng, J. L. Sonnenberg, M. Hada, M. Ehara, K. Toyota, R. Fukuda, J. Hasegawa, M. Ishida, T. Nakajima, Y. Honda, O. Kitao, H. Nakai, T. Vreven, J. A. Montgomery, Jr., J. E. Peralta, F. Ogliaro, M. Bearpark, J. J. Heyd, E. Brothers, K. N. Kudin, V. N. Staroverov, R. Kobayashi, J. Normand, K. Radhavachari, A. Rendell, J. C. Burant, S. S. Iyengar, J. Somasi, M. Cossi, N. Rega, N. J. Millam, M. Klene, J. E. Knox, J. B. Cross, V. Bakken, C. Adamo, J. Jaramillo, R. Gomperts, R. E. Stratmann, O. Yazyev, A. J. Austin, R. Cammi, C. Pomelli, J. W. Ochterski, R. L. Martin, K. Morokuma, V. G. Zakrzewski, G. A. Voth, P. Salvador, J. J. Dannenberg, S. Dapprich, A. D. Daniels, Ö. Farkas, J. B. Foresman, J. V. Ortiz, J. Cioslowski and D. J. Fox, Gaussian 09 (Revision D.01), Gaussian, Inc., Wallingford, CT, 2009.

[12] S. Grimme, S. Ehrlich, and L. Goerigk, L. *J. Comp. Chem.,* 2011, **32**, 1456-1465.

[13] M. P. Mitoraj, A. Michalak, and T. A. Ziegler, *J. Chem. Theory Comput.,* 2009, **5**, 962-975.

[14] F. Neese, *WIREs Comput. Mol. Sci.* 2018, **8**, e1327.

[15] T. Lu, and F. Chen, *J. Comput. Chem.,* 2012, **33**, 580–592.
